# The global, regional and national burden of stomach cancer and its attributable risk factors from 1990 to 2019

**DOI:** 10.1038/s41598-022-15839-7

**Published:** 2022-07-07

**Authors:** Yexun Song, Xiajing Liu, Wenwei Cheng, Heqing Li, Decai Zhang

**Affiliations:** 1 Department of Otolaryngology-Head Neck Surgery, The Third Xiangya Hospital of Central South University, Changsha, 410013 Hunan China; 2 Graduate School of Guilin Medical University, Guilin, 541004 Guangxi China; 3 Xiangya School of Public Health, Central South University, Changsha, 410000 Hunan China; 4 Department of Gastroenterology, The Third Xiangya Hospital of Central South University, Changsha, 410013 Hunan China; 5 Hunan Key Laboratory of Nonresolving Inflammation and Cancer, Changsha, 410013 Hunan China

**Keywords:** Cancer, Computational biology and bioinformatics, Gastroenterology

## Abstract

We aimed to estimate the incidence, mortality, and disability-adjusted life-years (DALYs) of stomach cancer at the global, regional, and national levels. Stomach cancer resulted in 1.3 million (1.2–1.4 million) incident cases, 9.5 hundred thousand (8.7–10.4 hundred thousand) deaths, and 22.2 million (20.3–24.1 million) DALYs in 2019. The age-standardized incidence rate, death rate and DALY rate were 15.6 (14.1–17.2), 11.9 (10.8–12.8), and 268.4 (245.5–290.6) per 100,000 person-years, respectively. Between 1990 and 2019, the global age-standardized incidence rate, death rate, and DALY rate decreased by − 30.5% (− 36.7 to − 22.9), − 41.9% (− 47.2 to − 36.3), and − 45.6% (− 50.8 to − 39.8), respectively. In 2019, most of the global numbers of incidence, death and DALYs were higher among males than females. A considerable burden of stomach cancer was attributable to smoking and a high-sodium diet. Although the global age-standardized incidence and death rates have decreased, continued growth in absolute numbers in some regions, especially in East Asia, poses a major global public health challenge. To address this, public health responses should be tailored to fit each country’s unique situation. Primary and secondary prevention strategies with increased effectiveness are required to reduce the incidence and mortality of stomach cancer, particularly in populations with a high disease burden.

## Introduction

Stomach cancer is a common malignant tumor that poses a serious disease burden worldwide^1^. As the fourth leading cause of cancer-related death and the fifth most commonly diagnosed cancer in the world, stomach cancer accounted for 1.1 million new cases (representing 5.6% of all cancer cases) and 7.6 hundred thousand new deaths (representing 7.7% of all cancer cases) worldwide in 2020^2^. The high incidence, poor prognosis, and cellular and molecular heterogeneity of stomach cancer, among other factors, make this disease a major global health issue^3^.

Although age-standardized incidence and death rates of stomach cancer have declined over the past century, clinicians still expect to see more stomach cancer cases in the future due to aging and population growth^4^. Moreover, the cost associated with stomach cancer management per patient is generally higher than for other cancers^5–7^. The total annual health care cost of stomach cancer estimated in geographic regions in 2017—Asia (China, Japan and Iran), Europe (Germany, France, Spain, the UK and Italy), North America (the US and Canada) and Australia—was 20.6 billion USD^8^. Stomach cancer comprises a substantial fraction of the global cancer burden in absolute terms. Thus, a comparable, consistent and systematic analysis of the global long-term trends and patterns of stomach cancer is essential to guide public policy and can provide benchmarks for decision-makers.

The Global Burden of Diseases, Injuries, and Risk Factors Study (GBD) 2019 is the most comprehensive and systematic dataset assessing the burden of diseases, injuries, and risk factors at global, regional, and national levels. The GBD results for 2019 supersede all previously published GBD estimates, as new data sources were added and estimation methods were improved. The aim of the current study was to describe the impact of geographical location, social-development index (SDI), age, and sex on the global trends of incident cases, deaths, and DALYs of stomach cancer and its attributable risk factors based on data from the GBD 2019 study.

## Results

### Global level

**Figure 1:**
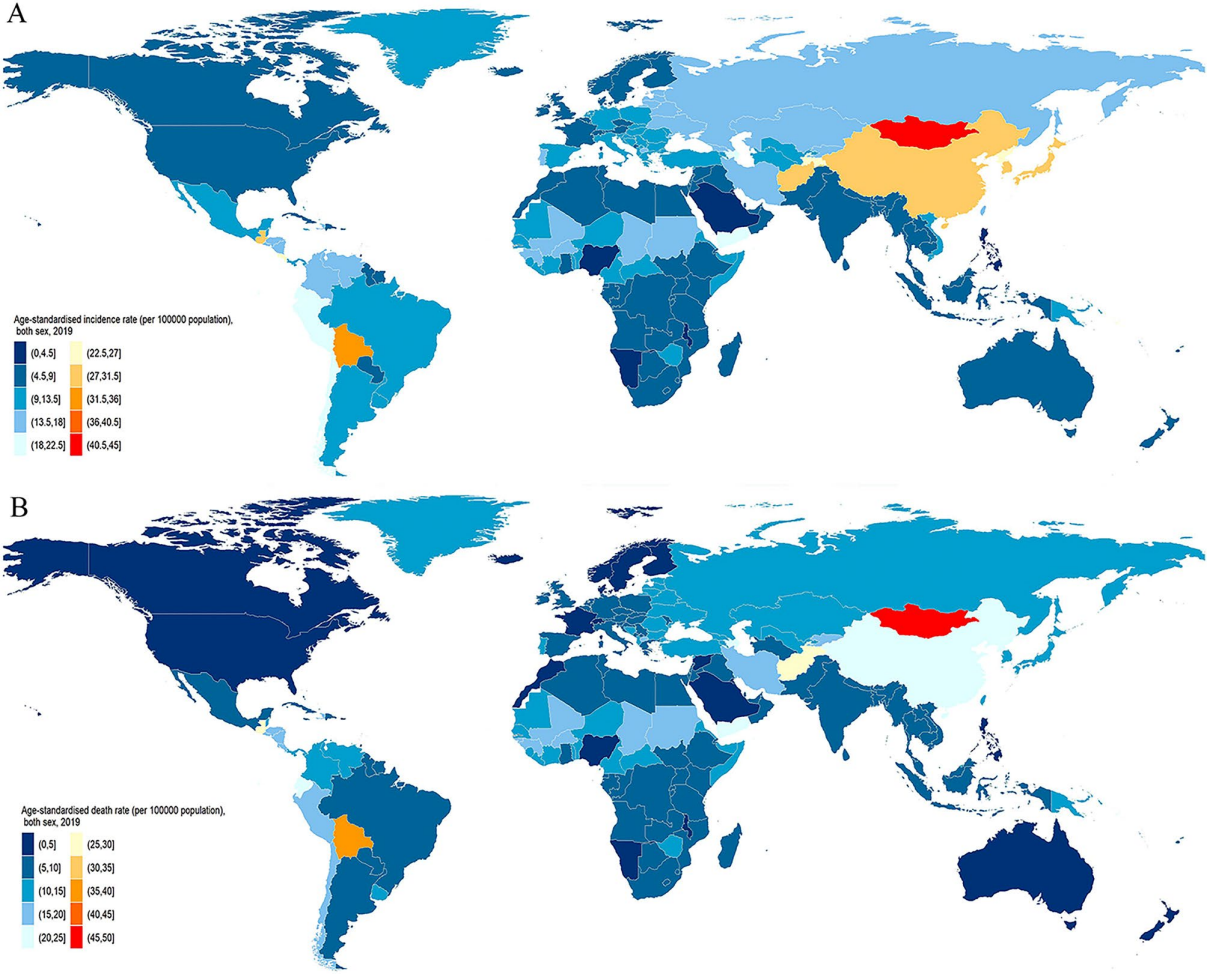
Age-standardized incidence (**A**) and death (**B**) rates of stomach cancer per 100,000 population both sex, 2019. Maps were generated using R software (version 4.0.3) and ggplot2 package. [R Core Team (2019). R: A language and environment for statistical computing. R Foundation for Statistical Computing, Vienna, Austria. URL https://www.R-project.org; and (H. Wickham. ggplot2: Elegant Graphics for Data Analysis. Springer-Verlag New York, 2016. URL https:// https://ggplot2.tidyverse.org)].

In 2019, there were 1.3 million (95% UI 1.2–1.4 million) incident cases of stomach cancer, accounting for 957,185 (870,949 to 1,034,646) deaths globally (Table 1, Supplementary Figs. S1 and S9). The age-standardized incidence rate was 15.6 (14.1 to 17.2) per 100,000 person-years (Fig. 1A), and the age-standardized death rate was 11.9 (10.8 to 12.8) per 100,000 person-years (Fig. 1B). A total of 22.2 million (20.3–24.1 million) DALYs were due to stomach cancer, with ASRs of 268.4 (245.5 to 290.6) per 100,000 person-years.

**Table 1:** The incidence, deaths and DALYs for stomach cancer in 2019 for both sexes, and percentage change of age-standardized rates (ASRs) by Global Burden of Disease regions from 1990 to 2019.

	Incidence (95%Uncertainty Interval)			Death (95%Uncertainty Interval)			DALYs (95%Uncertainty Interval)		
	Counts	ASR per 100,000 population (95%UI)	Percentage change in ASRs per 100,000 population (95%UI) (%)	Counts	ASR per 100,000 population (95%UI)	Percentage change in ASRs per 100,000 population(95%UI) (%)	Numbers	ASR per 100,000 population (95%UI)	Percentage change in ASRs per 100,000 population (95%UI) (%)
Global	1,269,806 (1,150,487 to 1,399,817)	15.6 (14.1 to 17.2)	− 30.5 (− 36.7 to − 22.9)	957,185 (870,949 to 1,034,646)	11.9 (10.8 to 12.8)	− 42 (− 47.2 to − 36.3)	22,220,980 (20,301,494 to 24,071,758)	268.4 (245.5 to 290.6)	− 45.6 (− 50.8 to − 39.8)
Andean Latin America	12,374 (10,070 to 15,032)	22.4 (18.3 to 27.2)	− 24.4 (− 38.2 to − 6.7)	11,791 (9647 to 14,224)	21.5 (17.6 to 25.9)	− 32 (− 44.1 to − 16.8)	263,028 (212,831 to 321,879)	461.2 (373.9 to 562.5)	− 35.6 (− 48.4 to − 20.2)
Australasia	3449 (2788 to 4206)	7 (5.7 to 8.5)	− 31.2 (− 43.9 to − 16.1)	2046 (1837 to 2224)	4 (3.6 to 4.3)	− 47.1 (− 50.3 to − 43.5)	38,906 (35,984 to 41,628)	84.5 (78.7 to 90)	− 47.8 (− 50.8 to − 44.4)
Caribbean	4355 (3761 to 4970)	8.4 (7.3 to 9.6)	− 25.3 (− 35.3 to − 14.7)	4118 (3564 to 4717)	8 (6.9 to 9.1)	− 30.9 (− 40 to − 21.2)	97,702 (82,770 to 113,470)	189.6 (160.4 to 220.1)	− 28.6 (− 38.8 to − 17.2)
Central Asia	12,127 (10,990 to 13,403)	16.4 (15 to 17.9)	− 41.5 (− 46.6 to − 35.5)	11,641 (10,555 to 12,809)	16.3 (14.9 to 17.9)	− 41.9 (− 46.9 to − 36.1)	324,971 (294,104 to 359,728)	400.9 (364 to 442.7)	− 46.4 (− 51.5 to − 40.7)
Central Europe	21,716 (19,111 to 24,411)	10.3 (9 to 11.6)	− 43 (− 49.3 to − 36.2)	20,112 (17,722 to 22,593)	9.4 (8.3 to 10.5)	− 48.4 (− 54.1 to − 42.7)	428,299 (374,968 to 482,886)	215.1 (187.8 to 242.4)	− 49.4 (− 55.6 to − 43.2)
Central Latin America	30,509 (25,952 to 35,821)	13 (11.1 to 15.2)	− 31.6 (− 41.2 to − 19.8)	27,302 (23,371 to 31,869)	11.8 (10.1 to 13.7)	− 40.1 (− 48.5 to − 30)	647,870 (550,777 to 763,649)	268.4 (228.7 to 315.6)	− 38.2 (− 47.2 to − 26.8)
Central Sub-Saharan Africa	4250 (3379 to 5307)	8 (6.5 to 9.8)	− 33.1 (− 47.5 to − 16.2)	4279 (3422 to 5309)	8.5 (6.9 to 10.4)	− 33.4 (− 47.1 to − 18)	125,499 (97,759 to 157,846)	204.1 (163.1 to 253.4)	− 34.9 (− 49.2 to − 18.1)
East Asia	626,489 (526,591 to 741,267)	30.2 (25.5 to 35.5)	− 18.5 (− 33.1 to 0.1)	432,991 (364,163 to 504,145)	21.5 (18.2 to 24.9)	− 42.2 (− 52.3 to − 29.9)	10,102,781 (8,469,057 to 11,888,734)	477.9 (402.5 to 560.4)	− 46.6 (− 56.4 to − 34.5)
Eastern Europe	54,119 (48,785 to 59,786)	16.1 (14.5 to 17.8)	− 48 (− 52.9 to − 42.4)	45,009 (40,630 to 49,867)	13.2 (11.9 to 14.6)	− 54.4 (− 58.6 to − 49.7)	1,081,234 (971,542 to 1,201,164)	330.4 (297.1 to 366.6)	− 56.2 (− 60.6 to − 51.6)
Eastern Sub-Saharan Africa	11,755 (10,230 to 13,419)	7.2 (6.3 to 8.2)	− 32.7 (− 39.9 to − 23.4)	12,082 (10,581 to 13,749)	7.8 (6.9 to 8.8)	− 31.4 (− 38.5 to − 22.5)	345,779 (298,174 to 399,764)	184.4 (160.9 to 210.6)	− 35.6 (− 43.1 to − 25.8)
High-income Asia Pacific	128,168 (108,454 to 147,685)	28.2 (24.2 to 32.3)	− 54.2 (− 60 to − 48)	69,856 (59,749 to 75,611)	14 (12.4 to 15)	− 60.6 (− 63.5 to − 58.5)	1,161,348 (1,046,331 to 1,234,335)	284.7 (262.8 to 300.3)	− 66.1 (− 67.9 to − 64.4)
High− income North America	37,585 (32,974 to 42,693)	6.1 (5.4 to 7)	− 28.1 (− 36.9 to − 18.2)	22,316 (20,680 to 23,321)	3.5 (3.3 to 3.7)	− 41 (− 42.6 to − 39.2)	446,189 (426,861 to 461,362)	77.4 (74.4 to 79.9)	− 40.2 (− 41.8 to − 38.3)
North Africa and Middle East	42,259 (38,242 to 46,713)	10.1 (9.1 to 11.1)	− 26.5 (− 34.3 to − 15.4)	39,606 (35,880 to 43,876)	9.9 (8.9 to 10.9)	− 31.4 (− 38.8 to − 20.6)	1,011,450 (904,537 to 1,132,984)	218.1 (196.9 to 242.4)	− 36.9 (− 44.1 to − 27)
Oceania	937 (715 to 1187)	12.9 (10.1 to 15.9)	− 6.5 (− 22.4 to 14.1)	908 (694 to 1144)	13.4 (10.6 to 16.5)	− 7.5 (− 22.5 to 12)	28,669 (21,580 to 36,725)	335 (255.9 to 422.1)	− 7 (− 24 to 14.7)
South Asia	99,399 (87,310 to 113,633)	7 (6.2 to 8)	− 29.5 (− 39.4 to − 18.1)	99,072 (86,310 to 112,442)	7.2 (6.3 to 8.2)	− 30.9 (− 40.3 to − 19.8)	2,774,513 (2,413,908 to 3,168,197)	182.1 (158.7 to 207.5)	− 31.3 (− 41 to − 20.3)
Southeast Asia	40,055 (35,465 to 44,828)	6.7 (6 to 7.5)	− 38.4 (− 46.4 to − 28.6)	38,207 (34,167 to 42,456)	6.7 (6 to 7.4)	− 41.7 (− 48.3 to − 32.9)	987,321 (874,362 to 1,105,379)	153.5 (136.5 to 171.6)	− 45.3 (− 52.1 to − 36.6)
Southern Latin America	10,694 (8558 to 13,335)	12.8 (10.2 to 16)	− 30.8 (− 44.7 to − 13.7)	9953 (9295 to 10,524)	11.8 (11.1 to 12.5)	− 37.6 (− 41 to − 34)	209,269 (198,034 to 220,237)	256.5 (242.9 to 269.8)	− 39.1 (− 42.4 to − 35.9)
Southern Sub-Saharan Africa	3610 (3282 to 3971)	6.5 (6 to 7.1)	− 25.4 (− 33.2 to − 16.4)	3657 (3349 to 4009)	6.9 (6.3 to 7.5)	− 25.5 (− 33.2 to − 16.6)	95,721 (86,515 to 106,655)	158.4 (144.3 to 175)	− 29 (− 37.1 to − 19.8)
Tropical Latin America	24,537 (23,065 to 25,654)	10.2 (9.6 to 10.7)	− 43.5 (− 46.1 to − 40.6)	23,450 (21,816 to 24,582)	9.8 (9.1 to 10.3)	− 47.9 (− 50.4 to − 45.1)	556,713 (530,256 to 581,239)	225.7 (214.3 to 235.9)	− 47.8 (− 50.2 to − 45.1)
Western Europe	86,455 (75,199 to 97,340)	9.4 (8.2 to 10.7)	− 41.5 (− 48.5 to − 34.2)	63,125 (57,057 to 66,790)	6.5 (6 to 6.8)	− 52.9 (− 54.7 to − 51.1)	1,097,486 (1,030,850 to 1,149,166)	132 (125.3 to 137.7)	− 54.3 (− 56 to − 52.7)
Western Sub-Saharan Africa	14,962 (12,911 to 17,366)	8.7 (7.5 to 9.8)	− 15.1 (− 25.6 to − 3.1)	15,664 (13,590 to 17,950)	9.5 (8.3 to 10.8)	− 14.3 (− 24.6 to − 2.6)	396,231 (337,138 to 463,233)	199.7 (172 to 229.9)	− 19.6 (− 30.9 to − 6.6)

*DALYs* disability adjusted life years, *UI* uncertainty interval.

From 1990 to 2019, the global age-standardized incidence rate decreased by − 30.5% (95% UI − 36.7% to − 22.9%), the mortality rate decreased by − 41.9% (− 47.2% to − 36.3%), and the DALY rate decreased by − 45.6% (− 50.8% to − 39.8%). From 1990 to 2019, the total number of new cases increased by 43.7% (30.6% to 60.1%), from 8.8 hundred thousand (8.3–9.3 hundred thousand) to 1.3 million (95% UI 1.2–1.4 million); the total number of deaths increased by 21.4% (10.1% to 33.6%), from 788,317 (742,787 to 833,999) to 957,185 (870,949 to 1,034,646); and the total DALYs increased by 8.6% (− 1.9% to 20.4%), from 20.4 million (19.2–21.7 million) to 22.2 million (20.3–24.1 million).

### Regional level

The largest numbers of incident stomach cancer in 2019 were in East Asia (626,488, 95% UI 526,591 to 741,267), high-income Asia Pacific (128,168, 95% UI 108,454 to 147,685) and South Asia (99,399, 95% UI 87,309 to 113,633). The lowest incidence numbers were in Oceania (937, 95% UI 714 to 1186), Australasia (3449, 95% UI 2788 to 4205) and southern sub-Saharan Africa (3610, 95% UI 3282 to 3970). The largest number of deaths due to stomach cancer in 2019 were in East Asia (432,991, 95% UI 364,163 to 504,145), South Asia (99,071, 95% UI 96,309 to 112,441) and high-income Asia Pacific (69,855, 95% UI 59,748 to 75,610). The lowest numbers of deaths were in Oceania (908,693 to 1143), Australasia (2046, 95% UI 1836 to 2224) and southern sub-Saharan Africa (3656, 95% UI 348 to 4009). East Asia (10.1 million, 95% UI 8.5–11.9 million), South Asia (2.8 million, 95% UI 2.4–3.2 million) and high-income Asia Pacific (1.2 million, 95% UI 1.1–1.2 million) had the largest number of DALYs attributed to stomach cancer, while these numbers were lowest in Oceania (28,669, 95% UI 21,580 to 36,725), Australasia (38,906, 95% UI 35,984 to 41,627) and southern sub-Saharan Africa (95,721, 95% UI 86,515 to 106,654). In all regions in 2019, the incidences, deaths, and DALYs were higher among males than females.

**Figure 2:**
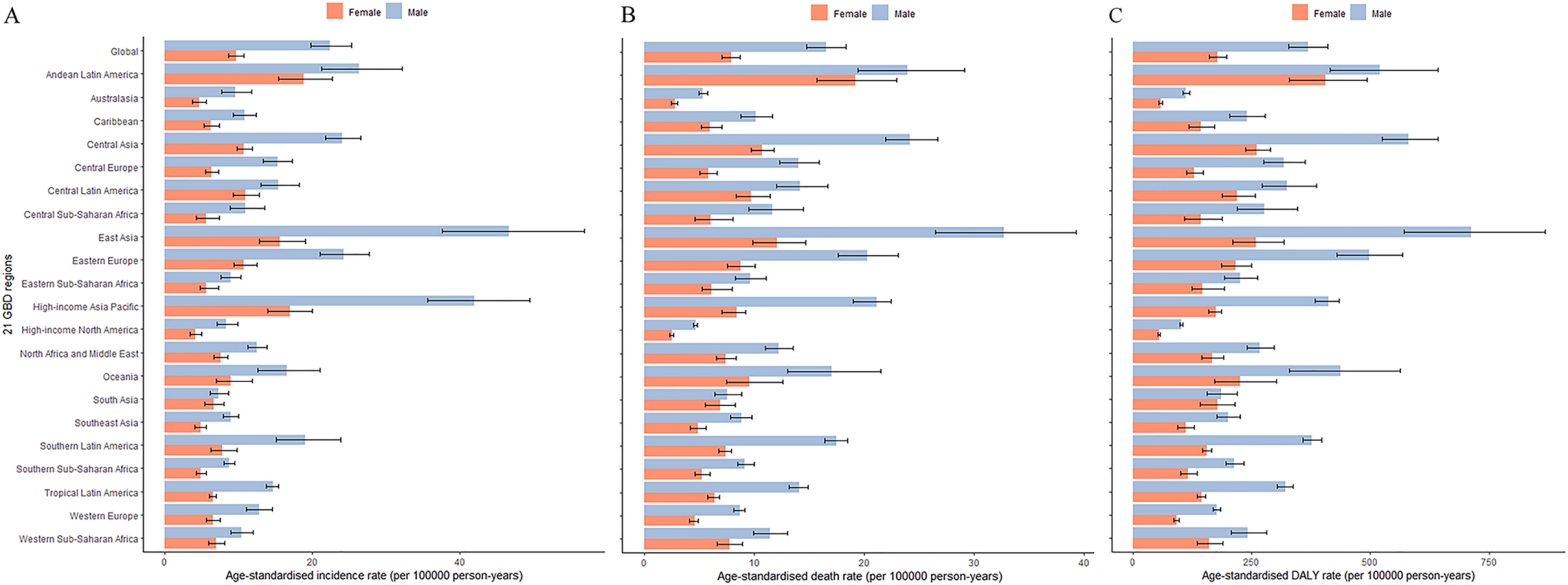
Age-standardized incidence (**A**), death (**B**) and DALY (**C**) rates of stomach cancer per 100,000 population for 21 Global Burden Disease regions by sex, 2019. Error bars indicate the 95% uncertainty intervals (95% UI) for incidence (**A**), death (**B**) and DALYs (**C**). *DALY* disability adjusted life years.

East Asia (30.2, 95% UI 25.5–35.5 per 100,000 person-years), high-income Asia Pacific (28.2, 95% UI 24.2–32.3 per 100,000 person-years) and Andean Latin America (22.4, 95% UI 18.3–27.2 per 100,000 person-years) had the highest age-standardized incidence rates, while high-income North America (6.1, 95% UI 5.4–6.9 per 100,000 person-years), southern sub-Saharan Africa (6.5, 95% UI 5.9–7.1 per 100,000 person-years) and Southeast Asia (6.7, 95% UI 5.9–7.5 per 100,000 person-years) had the lowest rates (Fig. 2A).

East Asia (21.5, 95% UI 18.2–24.9 per 100,000 person-years), Andean Latin America (21.5, 95% UI 17.6–25.9 per 100,000 person-years) and Central Asia (16.3, 95% UI 14.9–17.9 per 100,000 person-years) had the highest age-standardized death rates, while high-income North America (3.5, 95% UI 3.3–3.7 per 100,000 person-years), Australasia (4.0, 95% UI 3.6–4.3 per 100,000 person-years) and Western Europe (6.5, 95% UI 5.9–6.8 per 100,000 person-years) had the lowest rates (Fig. 2B).

East Asia (477.9, 95% UI 402.5–560.4 per 100,000 person-years), Andean Latin America (461.2, 95% UI 373.9–562.5 per 100,000 person-years) and Central Asia (400.9, 95% UI 364.0–442.7 per 100,000 person-years) had the highest age-standardized DALY rates in 2019, while high-income North America (77.4, 95% UI 74.4–79.9 per 100,000 person-years), Australasia (84.5, 95% UI 78.7–89.9 per 100,000 person-years) and Western Europe (131.9, 95% UI 125.3–137.7 per 100,000 person-years) had the lowest rates (Fig. 2C). The age-standardized incidence, death, and DALY rates were higher for males than females in all GBD regions in 2019. East Asia showed the largest gaps between males and females in the age-standardized incidence, death, and DALY rates in the 21 GBD regions in 2019.

From 1990 to 2019, the age-standardized rates of incidence, death and DALYs all decreased in 21 GBD regions. High-income Asia Pacific had the largest decreases in the incidence rate (− 54.2%, 95% UI − 59.9% to − 47.9%), death rate (− 60.6%, 95% UI − 63.5% to − 58.5%) and DALY rate (− 66.1%, 95% UI − 67.9% to − 64.4%) from 1990 to 2019.

**Figure 3:**
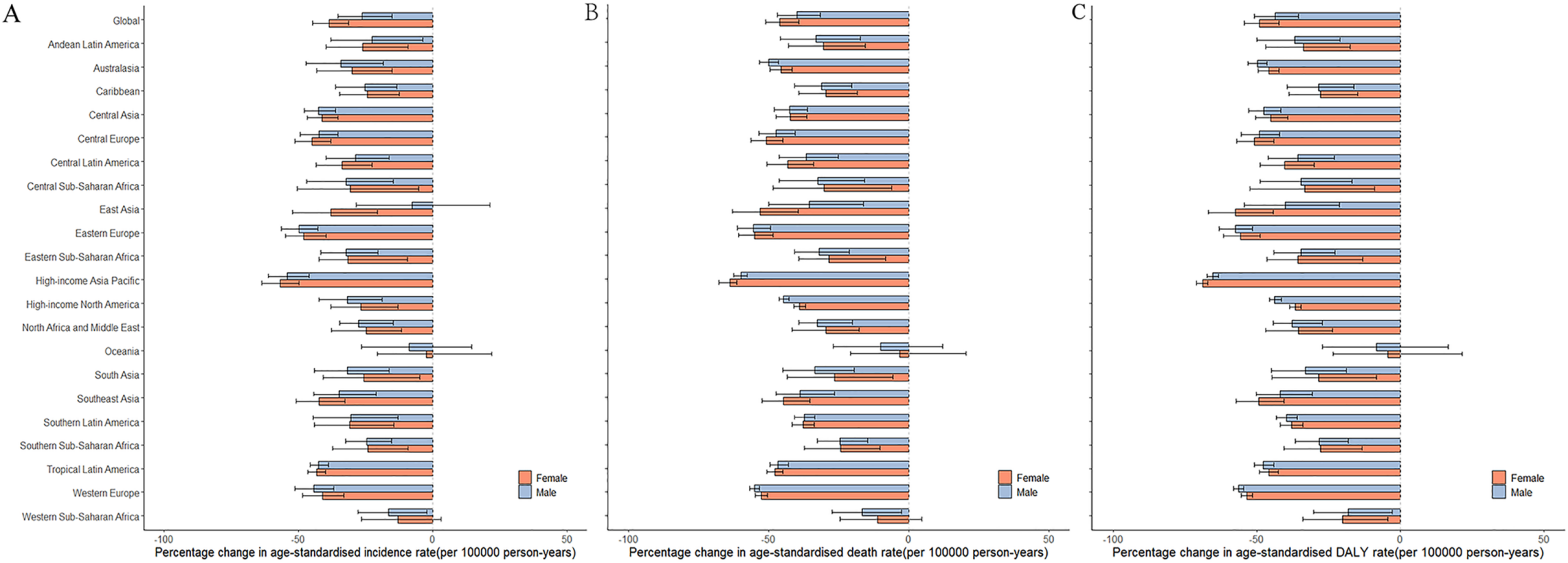
The percentage change in age-standardized incidence (**A**), death (**B**) and DALY (**C**) rates of stomach cancer per 1,000,000 by sex for 21 Global Burden of Disease regions, 1990–2019. Error bars indicate the 95% uncertainty intervals (95% UI) for incidence (**A**), death (**B**) and DALY (**C**). *DALYs* disability adjusted life years.

From 1990 to 2019, in all GBD regions, the age-standardized incidence, death and DALY rates for stomach cancer decreased (Fig. 3).

### National level

At the national level, China had the highest number of incident cases (612,821, 95% UI 512,997 to 728,891), followed by Japan (102,234, 95% UI 83,883 to 120,371) and India (82,315, 95% UI 70,723 to 95,424). China had the highest number of deaths (421,539, 95% UI 353,520 to 493,176), followed by India (81,770, 95% UI 70,139 to 94,684) and Japan (57,162, 95% UI 48,002 to 62,076). China had the highest number of DALYs (9.8 million, 95% UI 8.2–11.6 million), followed by India (2.3 million, 95% UI 2.0–2.7 million) and Japan (8.9 hundred thousand, 95% UI 8.0–9.5 hundred thousand).

In 2019, the highest age-standardized incidence rates were found in Mongolia (43.7, 95% UI 34.3 to 55.1 per 100,000 person-years), Bolivia (plurinational state) (34.0, 95% UI 26.8 to 42.0 per 100,000 person-years) and China (30.1, 95% UI 25.8 to 36.1 per 100,000 person-years). The lowest age-standardized incidence rates were observed in Malawi (3.3, 95% UI 2.7 to 3.9 per 100,000 person-years), Namibia (3.4, 95% UI 2.8 to 4.3 per 100,000 person-years) and Maldives (3.8, 95% UI 3.1 to 4.5 per 100,000 person-years). In 2019, the age-standardized death rates were highest in Mongolia (46.0, 95% UI 36.3 to 57.5 per 100,000 person-years), Bolivia (36.1, 95% UI 28.8 to 44.3 per 100,000 person-years) and Afghanistan (29.3, 95% UI 21.2 to 36.5 per 100,000 person-years). The lowest age-standardized death rates were observed in the United States (3.4, 95% UI 3.1 to 3.5 per 100,000 person-years), Kuwait (3.5, 95% UI 2.9 to 4.2 per 100,000 person-years) and Malawi (3.6, 95% UI 3.0 to 4.3 per 100,000 person-years). In 2019, the highest estimated age-standardized DALY rates were observed in Mongolia (1059.2, 95% UI 816.8 to 1351.3 per 100,000 person-years), Bolivia (749.1, 95% UI 572.7 to 946.3 per 100,000 person-years) and Afghanistan (728.7, 95% UI 505.3 to 939.4 per 100,000 person-years). The lowest age-standardized DALY rates in 2019 were observed in Kuwait (64.9, 95% UI 54.1 to 77.7 per 100,000 person-years), the Maldives (68.1, 95% UI 56.0 to 81.2 per 100,000 person-years), and Sweden (73.5, 95% UI 68.4 to 78.1 per 100,000 person-years) (Supplementary Table 4).

From 1990 to 2019, the percentage change in age-standardized incidence rates differed significantly between countries, with the Dominican Republic (20.0%, 95% UI − 10.1% to 58.8%), Honduras (18.9%, − 5.1% to 51.4%), and Lesotho (16.1%, − 12.9% to 53.9%) showing the largest increases. By contrast, Trinidad and Tobago (− 60.2%, − 70.1% to − 48.3%), Singapore (− 60.1%, − 68.0% to − 50.1%) and Maldives (− 59.3%, − 68.3% to − 45.9%) showed the largest decreases. The percentage change in age-standardized death rates also differed between countries. The largest increases were seen in Honduras (17.1%, − 5.4% to 48.9%), Lesotho (15.7%, − 12.5% to 52.6%), and Dominican Republic (13.0%, − 14.5% to 46.9%). By contrast, the largest decreases were found in Republic of Korea (− 73.1%, − 75.7% to − 69.9%), Singapore (− 72.9%, − 75.4% to − 70.4%) and Austria (− 68.6%, − 70.7% to − 66.4%). In terms of age-standardized DALY rates, the largest increases were observed in Lesotho (20.4%, − 13.1% to 63.4%), Dominican Republic (14.1%, − 16.8% to 54.3%), and Zimbabwe (8.9%, − 18.4% to 41.2%). The largest decreases were found in Republic of Korea (− 78.1%, − 80.2% to − 75.5%), Singapore (− 76.1%, − 78.1% to − 74.0%) and Austria (− 70.3%, − 72.3% to − 68.2%).

### Age and sex patterns

**Figure 4:**
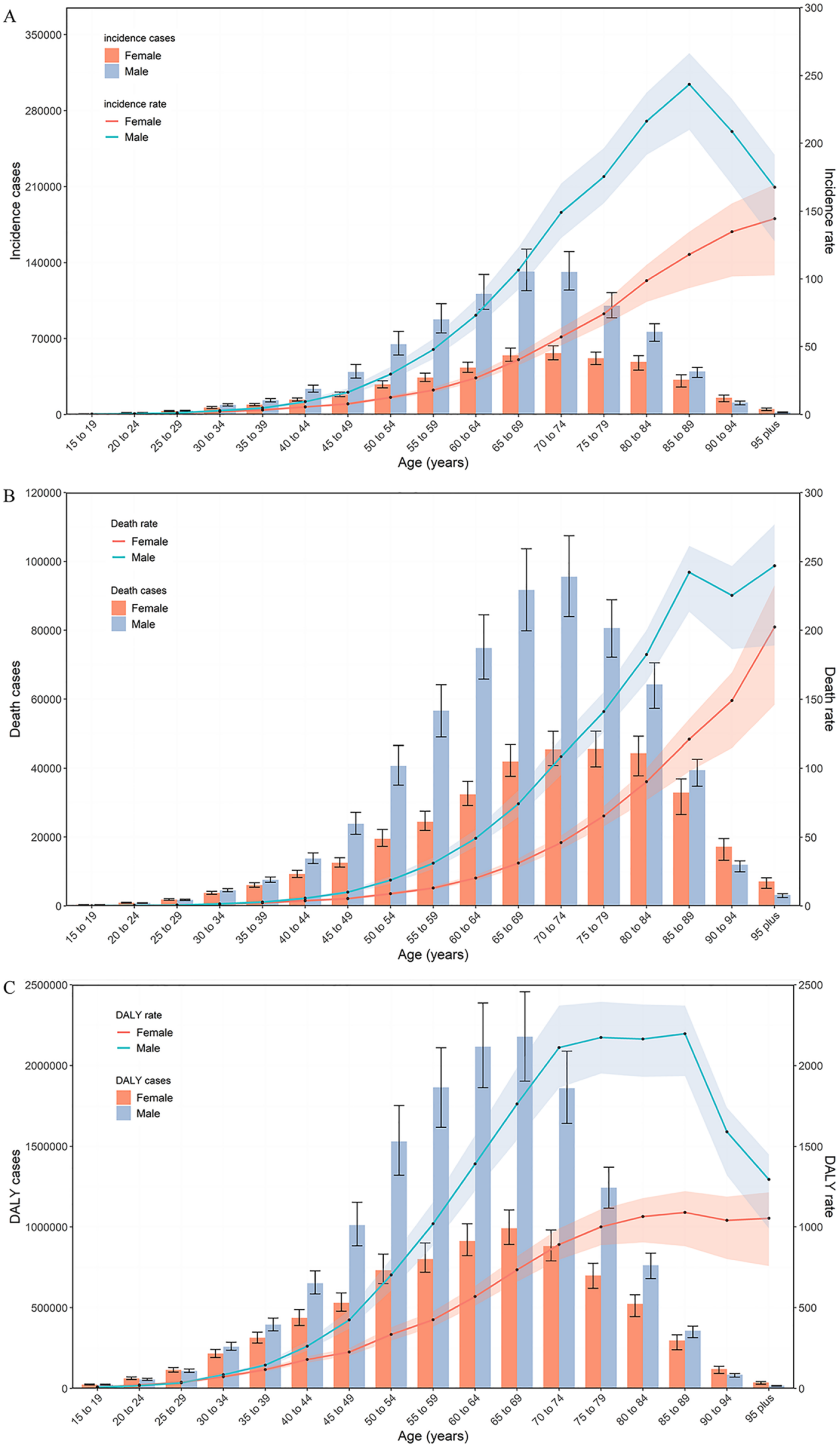
Global counts and age-standardized incidence (**A**), death (**B**) and DALY (**C**) rates of stomach cancer per 100,000 population by age and sex, 2019. Error bars indicate the 95% uncertainty intervals (95% UI) for incidence (**A**), death (**B**) and DALYs (**C**). Shading indicates the upper and lower limits of the 95% UI. *DALY* disability adjusted life years.

In 2019, most of the incidence, death and DALY numbers globally were higher in males than females in all age groups except for the incidence in people older than 90 years, and the death and DALY numbers for those 15–19 years, 20–24 years, 25–29 years, 90–94 years, and 95 plus years. In 2019, most of the global age-standardized incidence, death and DALY rates were higher in males than females, except for the death and DALY rates in those 15–19 years, 20–24 years, and 25–29 years (Fig. 4).

The numbers of global incident cases, deaths and DALYs followed a normal distribution and was similar between females and males but peaked at 65–69 years for incident cases and DALYs and peaked at 70–74 years for deaths.

In females, the global age-standardized rates of incidence, mortality, and DALYs increased linearly with age. In males, the global age-standardized rates of incidence, deaths and DALYs increased nonlinearly, peaking at 85–89 years with increasing age.

### Burden of stomach cancer by sociodemographic index (SDI)

**Figure 5:**
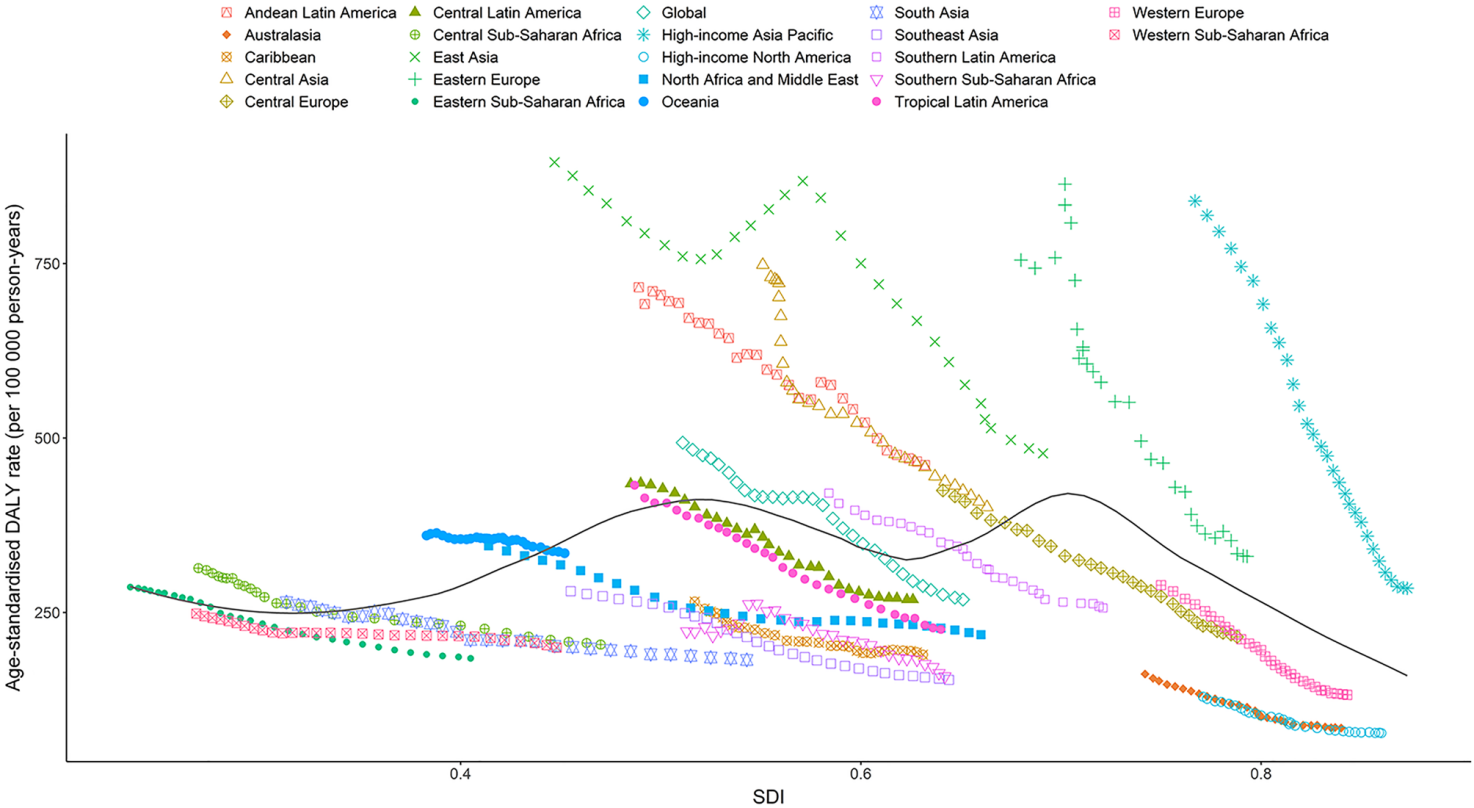
Age-standardized DALY rates for stomach cancer for 21 Global Burden of Disease region by SDI, 1990–2019. Black line represents the expected age-standardized DALY rates of stomach cancer based solely on SDI. For each region, points from the left to right depict estimates from each year from 1990 to 2019. *SDI*  Socio-demographic Index, *DALY* disability adjusted life years.

Figure 5 presents the age-standardized DALY rate trends across SDI by region from 1990 to 2019. The patterns are nonlinear, peaking at an SDI value of approximately 0.72, and then decreasing as the SDI values further increase. Most of the regions showed a decreasing trend in the age-standardized DALY rate in this study period, including high-income Asia Pacific, which had the largest decreases. The observed age-standardized DALY rate for East Asia initially decreased and then increased, after which it decreased again as the SDI value further increased.

**Figure 6:**
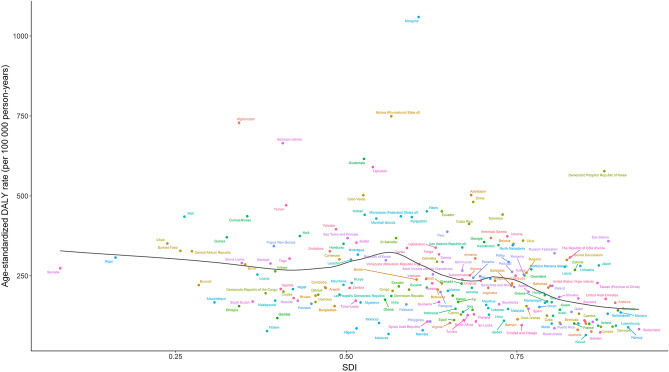
Age-standardized DALY rates for stomach cancer for 204 countries and territories by SDI, 2019. Black line represents the expected age-standardized DALY rates of stomach cancer based solely on SDI. For each region, points from the left to right depict estimates from each year from 1990 to 2019. *SDI* Socio-demographic Index, *DALY* disability adjusted life years.

At the national level, the expected patterns of the age-standardized DALY rates were nonlinear in nature, peaking at an SDI value of approximately 0.6 (Fig. 6). The age-standardized DALY rates of stomach cancer observed in some countries, such as Mongolia, Bolivia, and Afghanistan, were significantly higher than the expected levels, whereas in others, such as Maldives, Switzerland and Saudi Arabia, these rates were lower than the expected levels based on the SDI.

Figure S31 shows that the age-standardized incidence rate was highest in high-middle SDI countries/territories (18.8, 95% UI 16.6–21.0) and lowest in low-SDI countries/territories (8.4, 95% UI 7.6–9.3) in 2019. The age-standardized death rates were highest in middle-SDI countries/territories (14.6, 95% UI 13.0–16.3) and lowest in high-SDI countries/territories (7.2, 95% UI 6.5–7.6) in 2019. The DALY ASRs were highest in middle-SDI countries/territories (323.4, 95% UI 285.2–363.0) and lowest in high-SDI countries/territories (145.3, 95% UI 136.6–151.7) in 2019.

### Attributable risks

At the global level, for both sexes, a substantial proportion of DALYs were attributable to the two risk factors for which GBD estimates were available: 17.1% (95% UI 13.8% to 20.1%) attributable to smoking and 7.8% (95% UI 0.2% to 30.9%) to a high-sodium diet.

**Figure 7:**
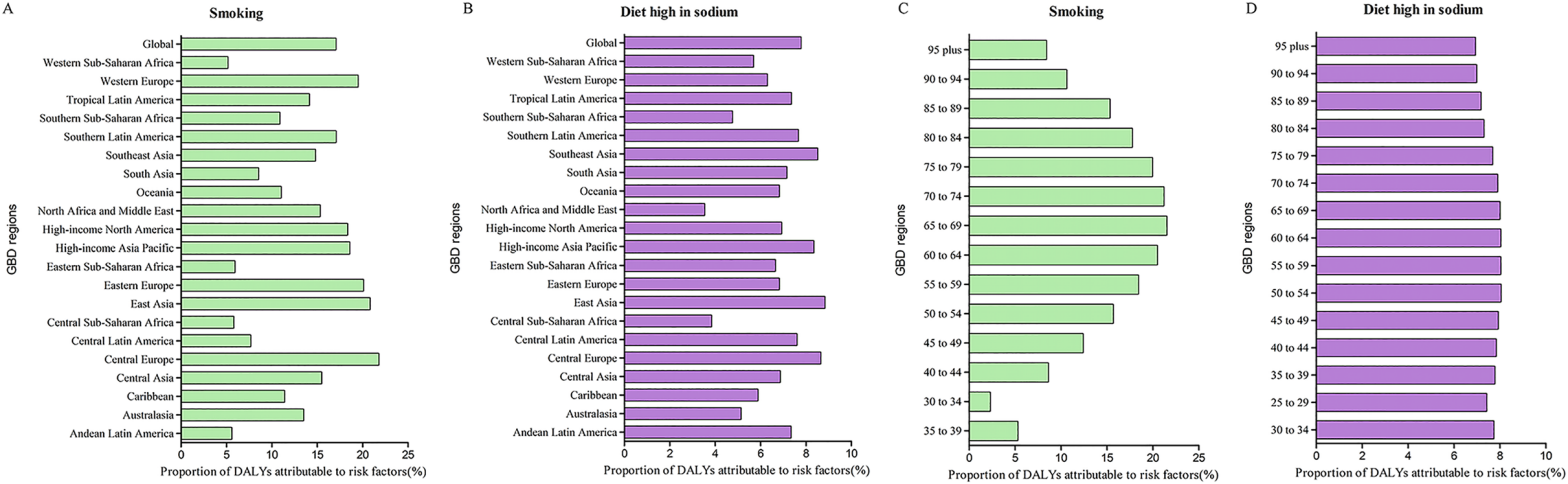
Proportion of stomach cancer DALYs attributable to smoking, diet high in sodium by region (**A**,**B**) and age (**C**,**D**), 2019. *DALY* disability adjusted life years.

The impact of these risk factors varied among regions. For example, the impact of smoking was highest in Central Europe (21.8%, 95% UI 17.7% to 25.7%) and lowest in western sub-Saharan Africa (5.2%, 95% UI 3.4% to 7.0%) (Fig. 7A). The impact of a high-sodium was highest in East Asia (8.8%, 95% UI 0.2% to 32.8%) and lowest in the Middle East and North Africa (3.5%, 95% UI 0.4% to 19.5%) (Fig. 7B).

The global patterns of attributable risk differed by age group. The global percentages of attributable DALYs were highest in the 65–69 year age group for smoking and the 50–54 year age group for a high-sodium diet, and they were the lowest in the 30–34 year age group for smoking and in the 95 and over group for a high-sodium diet (Fig. 7C,D).

## Discussion

To the best of our knowledge, this GBD-based study is the most recent examination of the global trends and patterns of stomach cancer-related incidence, mortality, DALYs and relevant risk factors. The total number of incident cases, deaths and DALYs increased from 1990 to 2019. However, the age-standardized rates of incidence, mortality and DALYs decreased during the study period. This may have been due to changes in the population age structure and improvements in medical conditions and the implementation of public health care systems^9^. Population growth in many regions, especially in East Asia, resulted in the continuous growth in the absolute number of stomach cancer cases and deaths.

The most recent GLOBOCAN 2020 study estimated that there were 1.1 million incident cases of stomach cancer^2^, which is close to our 2019 estimate (1.3 million, 95% UI 1.2–1.4). Similar to the GLOBOCAN report^2^, the highest age-standardized incidence rates were found in East Asia, high-income Asia Pacific and Andean Latin America in 2019. This finding can be explained in part by differences in the distribution of risk factors associated with gastric cancer^10^. *Helicobacter pylori* has been categorized by the World Health Organization as a class I human carcinogen and is the most important etiologic agent for stomach cancer, with almost 90% of new cases of noncardia stomach cancer attributed to this bacterium^11,12^. Regions with a high incidence of stomach cancer are prone to high *H. pylori* seroprevalence rates. A high prevalence of *H. pylori* infection has been observed in regions of East Asia, high-income Asia Pacific and Andean Latin America^13^. Furthermore, it was estimated that 33% to 50% of all stomach cancer cases were attributable to dietary (high-salt diet with few vegetables) and lifestyle factors (alcohol and coffee consumption and smoking), which are more common in these regions^10,14,15^. Similarly, the GLOBOCAN 2020 study estimated 0.8 million deaths of stomach cancer^2^, which are also close to our 2019 estimates of 1.0 million (95% UI 0.9–1.0). In both these two studies, age-standardized death rates were highest in East Asia, Andean Latin America and Central Asia.

We also analyzed heterogeneous trends in the national age-standardized incidence, mortality, and DALY rates from 1990 to 2019. Mongolia, Bolivia and China had the highest age-standardized incidence rates of stomach cancer in 2019, which was different from the GLOBOCAN 2020 study that found Mongolia, Japan and the Republic of Korea to have the highest age-standardized incidence rates^2^. Similarly, in the GBD 2019 study, the highest age-standardized death rates were found in Mongolia, Bolivia and Afghanistan, whereas in the GLOBCAN 2020 study, the highest rates were found in Mongolia, Tajikistan and Bhutan^2^. The differences in country-specific estimations can be attributed to the data sources and estimation methods used in the two studies^16^. Notably, both studies found that Mongolia had the highest age-standardized incidence and age-standardized mortality rate of stomach cancer in the world.

This study found higher morbidity and mortality in less developed countries, as indicated by the SDI. East Asia had the highest absolute incidence, death, DALY numbers and rates in 2019, with China contributing more than 90% of the cases. Despite the lower economic cost compared with other countries, China still has the heaviest stomach cancer burden^5^. China has the largest population of all countries, and it is undergoing population aging^17^. Therefore, China is the country with the highest stomach cancer incidence, deaths and DALYs and has higher rates of these indices than the average level worldwide. It is expected that the increasing trend in the number of deaths will continue^18^. However, morbidity will steadily decrease in the future with the improvement of medical treatments and public health strategies.

As expected, the global incidence, mortality and DALYs of stomach cancer were higher in males than in females. The incidences of several types of cancer, including stomach cancer, liver cancer and colon cancer, are far higher in males than in females^19–21^. It is speculated that lifestyle differences related to diet and smoking lead to these differences. However, there is growing evidence that the underlying biological sex differences are the basis of these differences. Alexander Sheh demonstrated that 17β-estradiol and tamoxifen can prevent *H. pylori* infection-associated stomach cancer through the leukocyte recruitment pathway^22^. Many studies have supported a central role of estrogen receptors in stomach cancer^23–25^. An improved understanding of estrogen in relation to stomach cancer might provide a novel pathophysiology mechanism and therapeutic target for stomach cancer.

There are multiple risk factors involved in the initiation and progression of stomach cancer. However, the risk burden was primarily attributable to smoking and a high-sodium diet. Several epidemiologic studies have supported the hypothesis that smoking increases the risk of incidence and death of stomach cancer^26,27^. A global burden of disease study reported that since 1990, the age-standardized prevalence of daily smoking was 25% for males and 5.4% for females^28^. Although some countries have implemented smoking restrictions to improve public health and mitigate the disease burden, smoking is still the leading risk factor for stomach cancer and other diseases^29^. Therefore, more effective and comprehensive policies should be applied to further reduce the prevenance of smoking. A high-sodium diet is another important dietary risk factor for stomach cancer, and the risk increases with the sodium consumption level^30,31^. Excess dietary sodium intake is a hazard worldwide and can be severely harmful to human health. In 2010, the average global sodium intake was 3.95 g per day, and regional daily intakes ranged from 2.18 to 5.51 g per day^32^. As the consumption of fresh products and lower salt food increases, stomach cancer mortality decreases^33,34^. The mechanism by which a high-sodium diet induces stomach cancer is associated with direct damage of the gastric mucosa, gastric pit epithelium proliferation, and *H. pylori* colonization^35^. Sodium restriction is another effective target to reduce the incidence and mortality rates of stomach cancer.

Although *H. pylori* infection is the strongest risk factor for stomach and treatment options are available, infection was not estimated in this study because the data were unavailable. However, with the improvement of socioeconomic status, the wide application of antibiotics, and the standardization of treatment, the rate of *H. pylori* infection globally is gradually decreasing, which greatly reduces the burden of stomach cancer^36^. The prevalence of *H. pylori* infection is still high worldwide, especially in developing countries, which may correlate with living conditions. A meta-analysis of 62 countries showed that approximately 4.4 billion individuals had an *H. pylori* infection in 2015^37^. *H. pylori* infection contributes to approximately 89% of stomach cancers. Several studies have reported treating this infection results in a reduction in stomach cancer incidence^38,39^. The research and development of effective medications and enhancing the socioeconomic status of people would help to reduce *H. pylori* infection rates, which is crucial for successfully reducing the burden of stomach cancer.

A major limitation to the study was our inability to distinguish cardia and noncardia forms of stomach cancer. The descriptive epidemiology and risk factor characteristics of cardia and noncardia stomach cancers differ^40^. Cardia cancer is more common in developed countries, in Caucasian populations, and in people with high socioeconomic status, while noncardia cancer is more common in developing countries, in Black populations, and in people with lower socioeconomic status^41^. *H. pylori* infection, smoking tobacco, and dietary factors can all increase the occurrence and development of noncardia cancer, while the main risk factors for cardia cancer include gastroesophageal reflux disease, obesity and, possibly, smoking tobacco^15^. In recent decades, unlike the decreasing incidence trends of noncardia cancer, cardia cancer rates have remained stable or have increased around the world^42^. However, changes in the global and regional trends and the burden of cardia versus noncardia tumors are difficult to investigate because the definition of gastric cardia has evolved over time. Furthermore, cardia cancers and esophageal adenocarcinomas are difficult to distinguish because tumors often overgrow the gastroesophageal junction^43^. There were several other limitations to our study. The accuracy of our results highly depended on the quantity and quality of the GBD data that was acquired, but this could be partially compensated for by applying statistical methods. Nevertheless, in regions with scarce data, especially underdeveloped regions, the estimates had to rely on predictive covariates or data from a single country, which is less representative. High-quality studies that are well-matched in location and population should be performed in future rounds of the GBD study. In addition, the influence of race was not taken into account in the GBD datasets; however, risk factors associated with race could be a consideration for stomach cancer. In addition, as discussed above, the burden of stomach cancer cannot be solely attributed to *H. pylori* infection, and the lack of data on other risk factors (e.g., excess body weight, medication use, and Epstein–Barr virus infection) limited our risk factor analysis.

Stomach cancer will continue to be a major public health burden because the majority of cases are diagnosed at an advanced stage when treatment options are limited and prognosis is poor. Thus, early screening for stomach cancer is urgently needed. Primary preventive strategies aimed at reducing risk factors and promoting protective factors will lead to a decrease in the incidence of stomach cancer. *H. pylori* eradication is recommended as the best primary prevention strategy^44^. The decrease in *H. pylori* infections in Japan is believed to have contributed to a decline in stomach cancer cases^45^. The role of other risk factors, such as diet, lifestyle, and drug use, in the primary prevention of stomach cancer is still under debate^46^. Neoplastic transformation of the gastric mucosa is a multistep process, and early diagnosis and appropriate management of preneoplastic conditions can reduce stomach cancer-related mortality. Therefore, several screening strategies have been proposed to detect neoplastic lesions at the early stage. At present, white light endoscopy with mapping biopsy remains the gold standard for stomach cancer diagnosis, and it is used for stomach cancer screening in high-risk areas (e.g., Japan, Korea, and Venezuela) due to its high detection rate^47^. By combining magnifying endoscopy and image-enhanced endoscopy, irregularities in the surface structures can be evaluated and highlighted, leading to improvements in the diagnostic accuracy of early-stage stomach cancer^48^. As a noninvasive test, the combination of pepsinogen, gastrin and anti-*H. pylori* antibody serological assays have been used for screening individuals and populations with a higher risk for gastric preneoplastic lesions^49,50^. Population screening is recommended in areas with a high incidence of stomach cancer, while individual screening is recommended for high-risk people who live in areas with a low incidence^51^. In Japan and South Korea, national population screening programs have been implemented and have been shown to be effective in reducing stomach cancer mortality^52,53^. A recent systematic review and meta-analysis confirmed the validity of endoscopic screening in Asian countries, reporting a 40% RR reduction in stomach cancer mortality^54^. To reduce stomach cancer incidence and mortality, primary and secondary prevention strategies with increased effectiveness are needed.

This study provides an updated estimate on the global stomach cancer burden and can be useful for defining new strategies for the early detection, treatment, and management of stomach cancer. Improved economic conditions that decrease the *H. pylori* infection rate and targeting risk factors, such as smoking and sodium intake, are expected to decrease stomach cancer incidence and mortality.

## Methods

### Study data

The GBD 2019 study, conducted by the Institute of Health Metrics and Evaluation (IHME), is the largest and most comprehensive effort to epidemiologically assess disease burdens and trends globally. GBD 2019 estimated the burden of 369 diseases and injuries; 286 causes of death; and 87 behavioral, environmental, occupational, and metabolic risk factors by region, sex, country, and age in 204 countries and territories, 7 super regions, and 21 regions from 1990 to 2019. In the current study, data on the disease burden of stomach cancer were obtained through an online query tool from the Institute for IHME website (http://ghdx.healthdata.org/). The general methodology of GBD 2019 has been explained in previous publications^55–58^. Detailed information on the estimation process of fatal and non-fatal outcomes in stomach cancer is described in the Supplementary methods.

The institutional review board of the Third Xiangya Hospital of Central South University determined that the study did not need approval because all data used in this study were publicly available. This study followed the Guidelines for Accurate and Transparent Health Estimates Reporting (GATHER) reporting guidelines for cross-sectional studies^59^.

### Case definition

Stomach cancers are diagnosed by endoscopy, imaging, and biopsy conducted in patients with relevant clinical signs and symptoms.

Stomach cancer was coded as 151–151.9, 209.23, and v10.04 in the 9th revision of the International Classification of Disease and Injuries (ICD-9) or C16-C16.9, z12.0, and z85.02-z85.028 in the ICD-10.

### Risk factors

The GBD 2019 used the comparative risk assessment framework used in the GBD since 2002 to quantify associations between disease and risk factors. Risk factors were divided into 3 categories: behavioral, environmental/occupational, and metabolic. Among the 87 risk factors assessed by GBD 2019, two major risk factors for stomach cancer were confirmed: smoking and a high-sodium diet.

### Uncertainty

We captured and propagated uncertainty through all calculations by sampling 1000 values (called draws) for each prevalence, death, YLL, YLD, or DALY estimate and summing draws across age, cause, and location for all intermediate calculations. The 95% uncertainty intervals (UIs) were defined by the ordinal 25th and 975th draw values.

### Statistical analysis

We used counts and age-standardized rates (ASRs) per 100,000 population, with 95% uncertainty intervals (UIs), to estimate the burden of stomach cancer. Incidence, prevalence, deaths, YLDs, YLLs, and DALYs were the metrics used to measure the burden of stomach cancer and were analyzed by age, sex, year, location, and SDI. We also calculated the percentage change in counts and ASRs between 1990 and 2019. The GBD 2019 used the comparative risk assessment framework to quantify associations between 87 risk factors and diseases; the risk factors were categorized as behavioral, environmental/occupational, and metabolic risks^60,61^. All statistical calculations were performed using the R statistical software program (version 4.0.3) or SPSS 25.0 (IBM Corporation, New York, USA). A *P* value < 0.05 was considered statistically significant.

## Supplementary Information

Supplementary Information 1.

Supplementary Figures.

Supplementary Tables.
