# Supplementary material for: The global, regional and national burden of stomach cancer and its attributable risk factors from 1990 to 2019
Source: Sci Rep. 2022 Jul 7;12:11542. doi: 10.1038/s41598-022-15839-7 (PMC9262989; doi:10.1038/s41598-022-15839-7)
Supplement: Supplementary file 1 — Supplementary Information 1. [file 41598_2022_15839_MOESM1_ESM.docx]

The global, regional and national burden of stomach cancer and its attributable risk factors from 1990 to 2019

Yexun Song^1^, Xiajing Liu^2^, Wenwei Cheng^1,3^, Heqing Li^1^, Decai Zhang^4, 5^

1 Department of Otolaryngology-Head Neck Surgery, The Third Xiangya Hospital of Central South University, Changsha, 410013, Hunan Province, China.

2 Graduate School of Guilin Medical University, Guilin, 541004, Guangxi Province, China.

3 Xiangya School of Public Health, Central South University, Changsha, 410000, Hunan Province, China.

4 Department of Gastroenterology, The Third Xiangya Hospital of Central South University, Changsha 410013, Hunan Province, China.

5 Hunan Key Laboratory of Nonresolving Inflammation and Cancer, Changsha 410013, Hunan Province, China

Correspondence: Decai Zhang, Department of Gastroenterology, The Third Xiangya Hospital of Central South University, Changsha 410013, Hunan Province, China. Email: decaizhang@csu.edu.cn

**Supplementary methods**

**GBD overview**

GBD 2019 produced estimates for 204 countries and territories that were grouped into 21 regions and seven super-regions. For GBD 2019, nine countries and territories (Cook Islands, Monaco, San Marino, Nauru, Niue, Palau, Saint Kitts and Nevis, Tokelau, and Tuvalu) were added, such that the GBD location hierarchy now includes all WHO member states.

For GBD 2019, we have also defined locations as standard locations and non-standard locations. Standard GBD locations are defined as the set of all subnationals belonging to countries where data quality is high and with populations over 200 million, in addition to all other countries. Standard locations include the subnationals for China, India, the United States, and Brazil, but not Indonesia; China, India, the United States, and Brazil are also included at the country level. All other countries with subnational estimates are defined as non-standard locations.

A complete set of risk-specific exposures, relative risks (RRs), theoretical minimum-risk exposure levels (TMRELs), and population attributable fractions (PAFs) were computed for the years 1990-2019.

**Data sources**

In the current study, the data extracted from the GBD 2019 were estimated from vital registration (VR) systems, sample vital registration, verbal autopsy^1^. Information about the data sources used for each location in this study can be found on the GBD 2019 Data Input Sources Tool website.

**Age groups**

Following GBD 2019 studies, mortality and population are estimated for 23 age groups: early neonatal (0-6 days), late neonatal (7-27 days), post-neonatal (28-365 days), 1-4 years, 5-9 years, every 5-year age group up to 95 years, and 95 years and older. Age-specific fertility is estimated for 5-year age groups between ages 10 years and 54 years.

**Mortality Estimates**

We derived mortality estimates from the data source described above and, when necessary, registry incidence data were multiplied by the corresponding, independently modelled, mortality-to-incidence ratios (MIRs) to produce mortality estimates. MIRs were modelled using locations where same-year cancer mortality and incidence data were available. These MIRs model started with a linear-step mixed-effects model with logit link functions, with Healthcare Access and Quality Index, age, and sex as covariates. The resulting estimates were them smoothed over space and time, and adjusted with spatiotemporal Gaussian process regression^2^. The Stomach cancer estimated mortality was combined with observed mortality (from vital registration and verbal autopsy) and entered into the Cause of Death Ensemble Model (CODEm)^3^. The CoDCorrect algorithm was used to adjust estimated single causes of mortality to ensure that all single causes sum to the all-cause mortality estimation^1,4,5^.

**Nonfatal Estimates**

The Stomach cancer survival data were collected and correlated with MIRs to generate prevalence. To estimate the YLDs, 10-year cancer prevalence was classified into four sequelae and multiplied the prevalence by corresponding disability weights (DWs): diagnosis and treatment, remission, disseminated and metastatic, and terminal phase. The durations of the four prevalence phases for Stomach cancer were 5.3 months of diagnosis and treatment^6^, 3.88 months of disseminated and metastatic disease^7^, and 1 month of terminal phase. Remission durations were calculated on the basis of the remainder of time after attributing other sequelae. Disability Weights are measured on a scale from 0 to 1, where 0 is a state of full health and 1 is death. Diagnosis and primary therapy phase of Stomach cancer has a disability weight of 0.288 (95% UI 0.193-0.399); Metastatic phase of Stomach cancer has a disability weight of 0.451 (95% UI 0.307-0.6); Terminal phase of Stomach cancer has a disability weight of 0.54 (95% UI 0.377-0.687); Controlled phase of Stomach cancer has a disability weight of 0.049 (95% UI 0.031-0.072)^8^. YLLs were computed by multiplying the age-specific estimates of number of deaths with a standard life expectancy at corresponding age. DALYs were the sum of YLDs and YLLs. The contributions of YLDs and YLLs to Stomach cancer DALYs were 3% and 97%, respectively. One DALY can be interpreted as 1 year of “healthy life” lose^9^.

The age-standardised rates and estimated annual percentage change were used to quantify the Stomach cancer burden trends. It is necessary to standardize the data when comparing several population groups with different age classes or for the same population over time in which the age profiles change.

**Case definition**

The Stomach cancers are diagnosed by endoscopy, imaging studies, and biopsy in a patient with relevant clinical signs and symptoms.

The Stomach cancer coded as 151-151.9, 209.23, v10.04 in the 9th revision of the International Classification of Disease and Injuries (ICD-9) or C16-C16.9, z12.0, z85.02-z85.028 in the ICD-10.

**Social demographic index**

Socio-demographic index (SDI) is a composite indicator that includes income per capita, average educational years and total fertility rate among individuals aged over 15 years. The calculation of SDI score in the GBD study was elaborated in the previous study^10^.

According to Socio-demographic index (SDI) quintiles, 204 countries and territories were categorized into five groups: low-SDI, low-middle-SDI, middle-SDI, high-middle-SDI, and high-SDI quintiles^8^.

We used the SDI to determine the relationship between the development level of a region or country and stomach cancer YLD, YLL, DALY. It is the geometric mean of 0 to 1 indices of total fertility rate under the age of 25 (TFU25), mean education for those ages 15 and older (EDU15+), and lag distributed income (LDI) per capita. As a composite, a location with an SDI of 0 would have a theoretical minimum level of development relevant to health, while a location with an SDI of 1 would have a theoretical maximum level.

**Risk Factors**

The GBD 2019 used the comparative risk-assessment framework, used in GBD since 2002, to quantify associations between disease and risk factors^11^. Risk factors were divided into 3 categories: behavioral, environmental/occupational, and metabolic^12^. Among the 87 risk factor assess by GBD 2019, two major risk factors for Stomach cancer were confirmed: Smoking, Diet high in sodium^13^.

**Uncertainty**

We captured and propagated uncertainty through all calculations by sampling 1000 values (called draws) for each prevalence, death, YLL, YLD, or DALY estimate and summing draws across age, cause, and location for all intermediate calculations. 95% uncertainty intervals (UIs) were defined by the ordinal 25th and 975th draw values.

**References**

1 Collaborators, G. B. D. D. Global age-sex-specific fertility, mortality, healthy life expectancy (HALE), and population estimates in 204 countries and territories, 1950-2019: a comprehensive demographic analysis for the Global Burden of Disease Study 2019. *Lancet* **396**, 1160-1203 (2020).

2 Engholm, G. *et al.* NORDCAN--a Nordic tool for cancer information, planning, quality control and research. *Acta. Oncol.* **49**, 725-736 (2010).

3 Foreman, K. J., Lozano, R., Lopez, A. D. & Murray, C. J. Modeling causes of death: an integrated approach using CODEm. *Popul. Health. Metr.* **10**, 1 (2012).

4 Disease, G. B. D., Injury, I. & Prevalence, C. Global, regional, and national incidence, prevalence, and years lived with disability for 354 diseases and injuries for 195 countries and territories, 1990-2017: a systematic analysis for the Global Burden of Disease Study 2017. *Lancet* **392**, 1789-1858 (2018).

5 Global Burden of Disease Cancer, C. *et al.* Global, Regional, and National Cancer Incidence, Mortality, Years of Life Lost, Years Lived With Disability, and Disability-Adjusted Life-years for 32 Cancer Groups, 1990 to 2015: A Systematic Analysis for the Global Burden of Disease Study. *JAMA. Oncol.* **3**, 524-548 (2017).

6 Neal, R. D. *et al.* Comparison of cancer diagnostic intervals before and after implementation of NICE guidelines: analysis of data from the UK General Practice Research Database. *Br. J. Cancer.* **110**, 584-592 (2014).

7 Noone AM, H. N., Krapcho M, et al. *SEER Cancer Statistics Review, 1975-2015, National. Cancer. Institute.*, (2018).

8 Diseases, G. B. D. & Injuries, C. Global burden of 369 diseases and injuries in 204 countries and territories, 1990-2019: a systematic analysis for the Global Burden of Disease Study 2019. *Lancet* **396**, 1204-1222, (2020).

9 DALYs, G. B. D. & Collaborators, H. Global, regional, and national disability-adjusted life-years (DALYs) for 359 diseases and injuries and healthy life expectancy (HALE) for 195 countries and territories, 1990-2017: a systematic analysis for the Global Burden of Disease Study 2017. *Lancet* **392**, 1859-1922, (2018).

10 Alcohol, G. B. D. & Drug Use, C. The global burden of disease attributable to alcohol and drug use in 195 countries and territories, 1990-2016: a systematic analysis for the Global Burden of Disease Study 2016. *Lancet. Psychiatry.* **5**, 987-1012, (2018).

11 Murray, C. J. & Lopez, A. D. Global mortality, disability, and the contribution of risk factors: Global Burden of Disease Study. *Lancet* **349**, 1436-1442, (1997).

12 Murray, C. J., Ezzati, M., Lopez, A. D., Rodgers, A. & Vander Hoorn, S. Comparative quantification of health risks conceptual framework and methodological issues. *Popul. Health. Metr.* **1**, 1, (2003).

13 Collaborators, G. B. D. R. F. Global burden of 87 risk factors in 204 countries and territories, 1990-2019: a systematic analysis for the Global Burden of Disease Study 2019. *Lancet* **396**, 1223-1249, (2020).
