# Supplementary material for: The global, regional and national burden of stomach cancer and its attributable risk factors from 1990 to 2019
Source: Sci Rep. 2022 Jul 7;12:11542. doi: 10.1038/s41598-022-15839-7 (PMC9262989; doi:10.1038/s41598-022-15839-7)
Supplement: Supplementary file 2 — Supplementary Figures. [file 41598_2022_15839_MOESM2_ESM.docx]

The global, regional and national burden of stomach cancer and its attributable risk factors from 1990 to 2019

Yexun Song^1^, Xiajing Liu^2^, Wenwei Cheng^1,3^, Heqing Li^1^, Decai Zhang^4, 5^

1 Department of Otolaryngology-Head Neck Surgery, The Third Xiangya Hospital of Central South University, Changsha, 410013, Hunan Province, China.

2 Graduate School of Guilin Medical University, Guilin, 541004, Guangxi Province, China.

3 Xiangya School of Public Health, Central South University, Changsha, 410000, Hunan Province, China.

4 Department of Gastroenterology, The Third Xiangya Hospital of Central South University, Changsha 410013, Hunan Province, China.

5 Hunan Key Laboratory of Nonresolving Inflammation and Cancer, Changsha 410013, Hunan Province, China

Correspondence: Decai Zhang, Department of Gastroenterology, The Third Xiangya Hospital of Central South University, Changsha 410013, Hunan Province, China. Email: decaizhang@csu.edu.cn

**Fig. S1. Incidence counts of stomach cancer for both sexes, in 2019.**


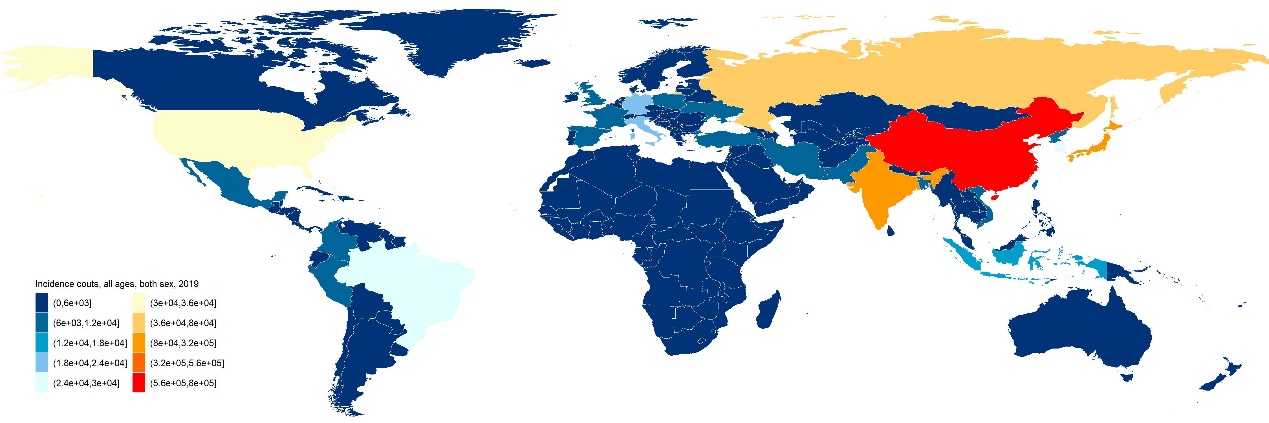


Maps were generated using R software (version 4.0.3) and ggplot2 package. [R Core Team (2019). R: A language and environment for statistical computing. R Foundation for Statistical Computing, Vienna, Austria. URL https://www.R-project.org; and (H. Wickham. ggplot2: Elegant Graphics for Data Analysis. Springer-Verlag New York, 2016. URL https:// https://ggplot2.tidyverse.org)].

**Fig. S2. Incidence counts for stomach cancer for 21 Global Burden Disease regions by sex, 2019.**


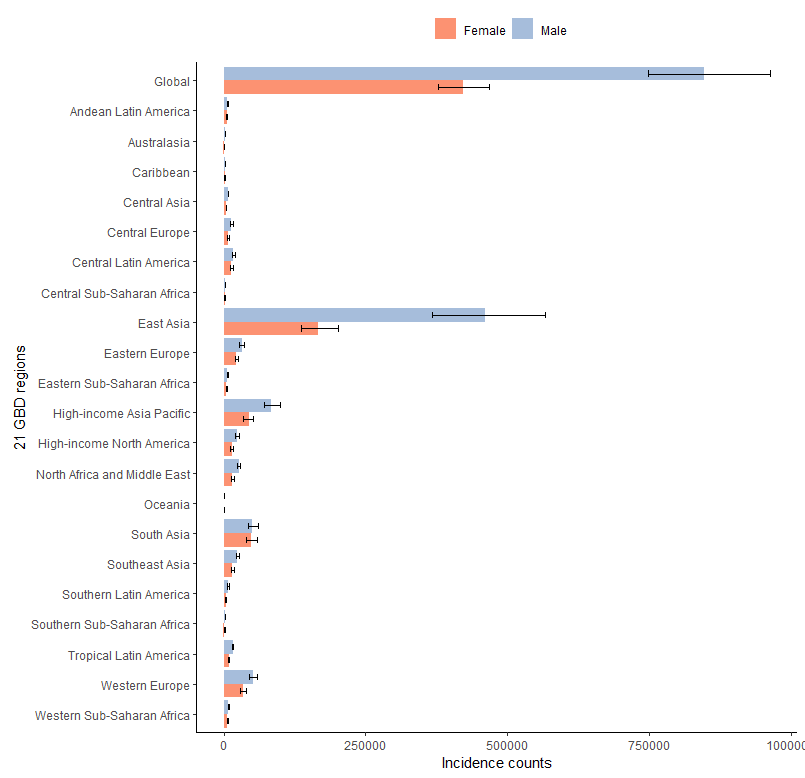


Error bars indicate the 95% uncertainty intervals (95% UI).

**Fig. S3. Age-standardized prevalence rate of stomach cancer per 100,000 person-years for both sexes, in 2019.**


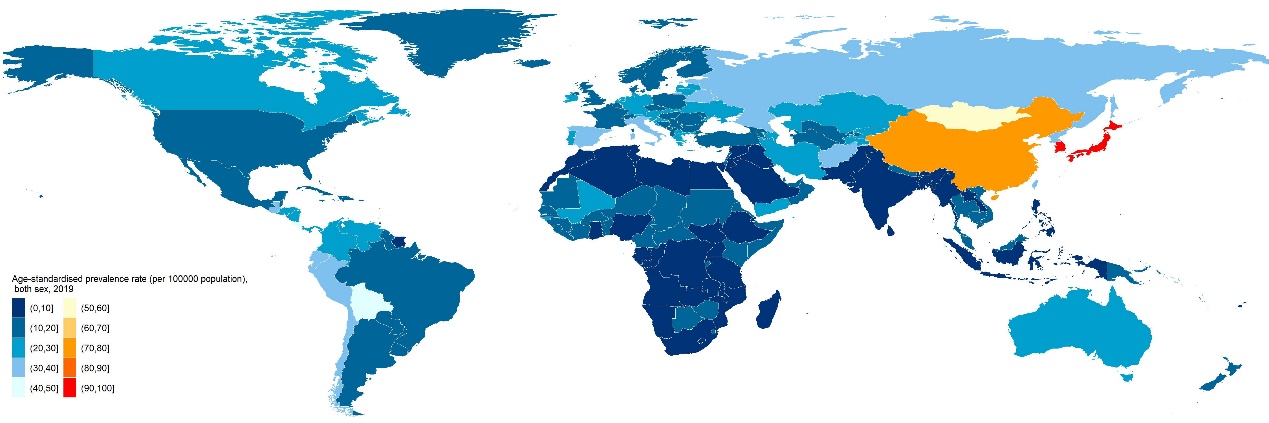


Maps were generated using R software (version 4.0.3) and ggplot2 package. [R Core Team (2019). R: A language and environment for statistical computing. R Foundation for Statistical Computing, Vienna, Austria. URL https://www.R-project.org; and (H. Wickham. ggplot2: Elegant Graphics for Data Analysis. Springer-Verlag New York, 2016. URL https:// https://ggplot2.tidyverse.org)].

**Fig. S4. Prevalence counts of stomach cancer for both sexes, in 2019.**


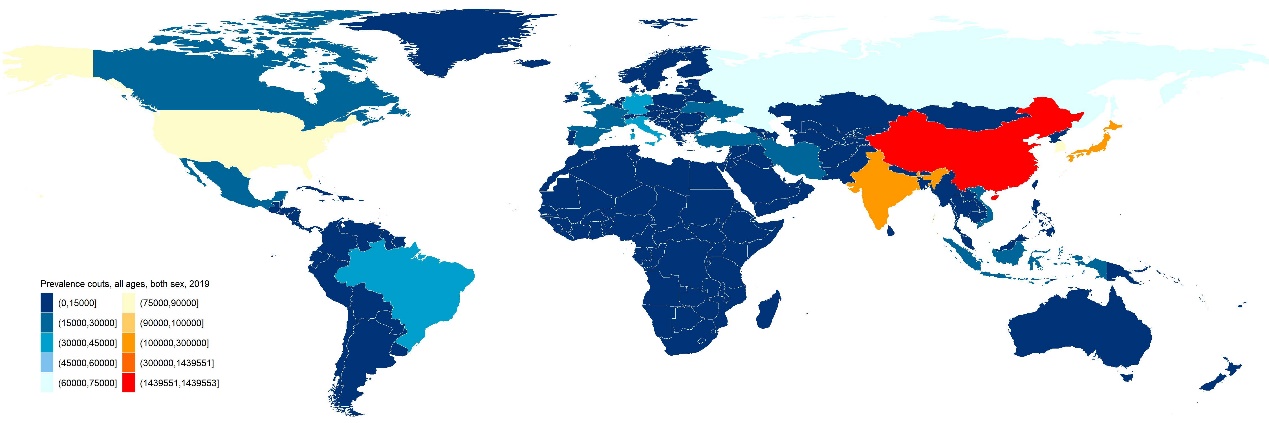


Maps were generated using R software (version 4.0.3) and ggplot2 package. [R Core Team (2019). R: A language and environment for statistical computing. R Foundation for Statistical Computing, Vienna, Austria. URL https://www.R-project.org; and (H. Wickham. ggplot2: Elegant Graphics for Data Analysis. Springer-Verlag New York, 2016. URL https:// https://ggplot2.tidyverse.org)].

**Fig. S5. Age-standardized prevalence rates of stomach cancer per 100,000 person-years for 21 Global Burden Disease regions by sex, 2019.**


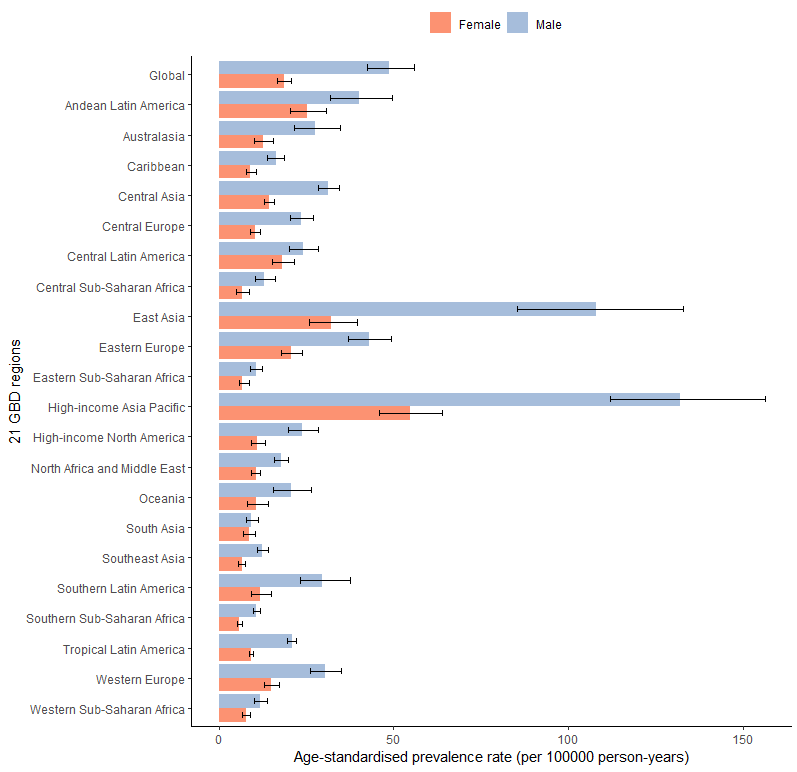


Error bars indicate the 95% uncertainty intervals (95% UI).

**Fig. S6. Prevalence counts for stomach cancer for 21 Global Burden Disease regions by sex, 2019.**


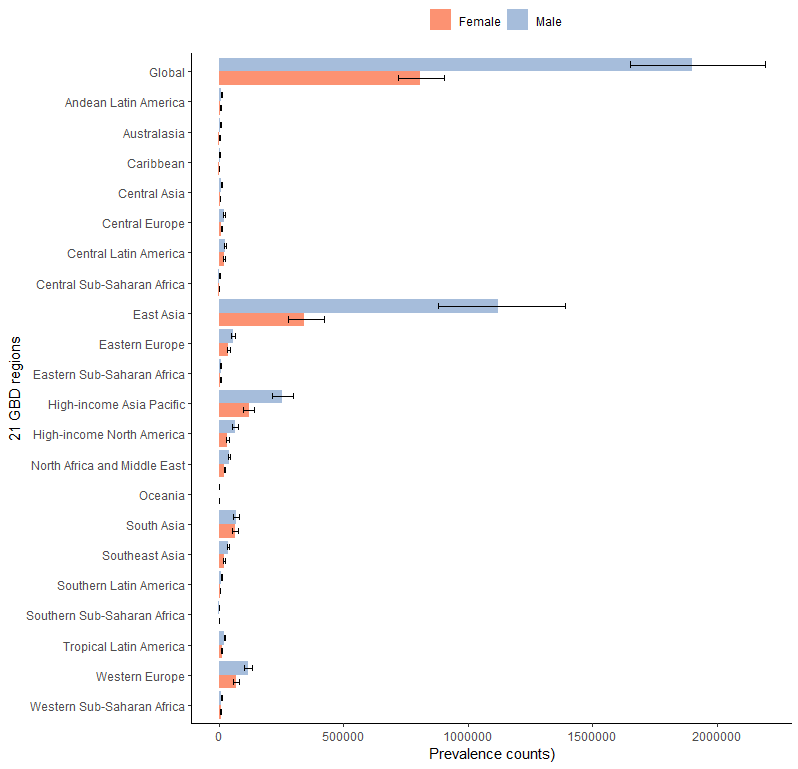


**Fig. S7. Global counts and age-standardized prevalence rates of stomach cancer per 100,000 person-years by age and sex, 2019**


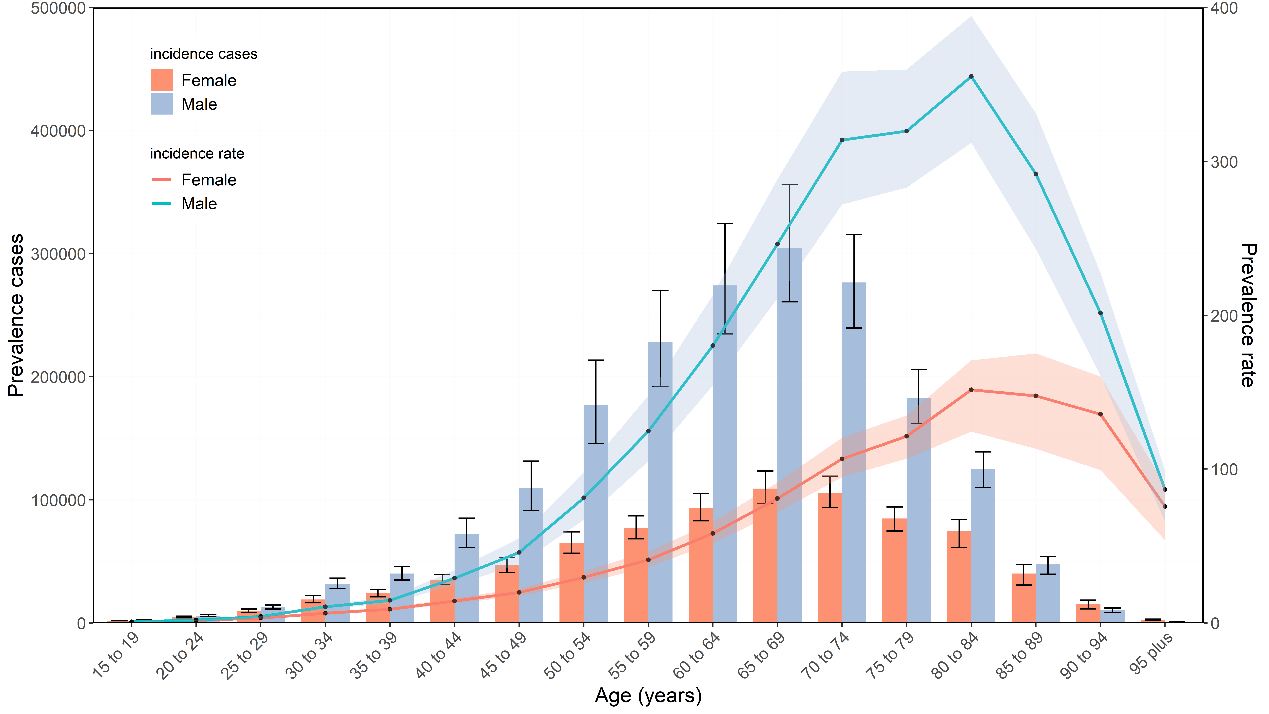


Error bars indicate the 95% uncertainty intervals (95% UI). Shading indicates the upper and lower limits of the 95% UI.

**Fig. S8. The percentage change in age-standardized prevalence rate of stomach cancer per 100,000 person-years by sex for 21 Global Burden of Disease regions, 1990-2019.**


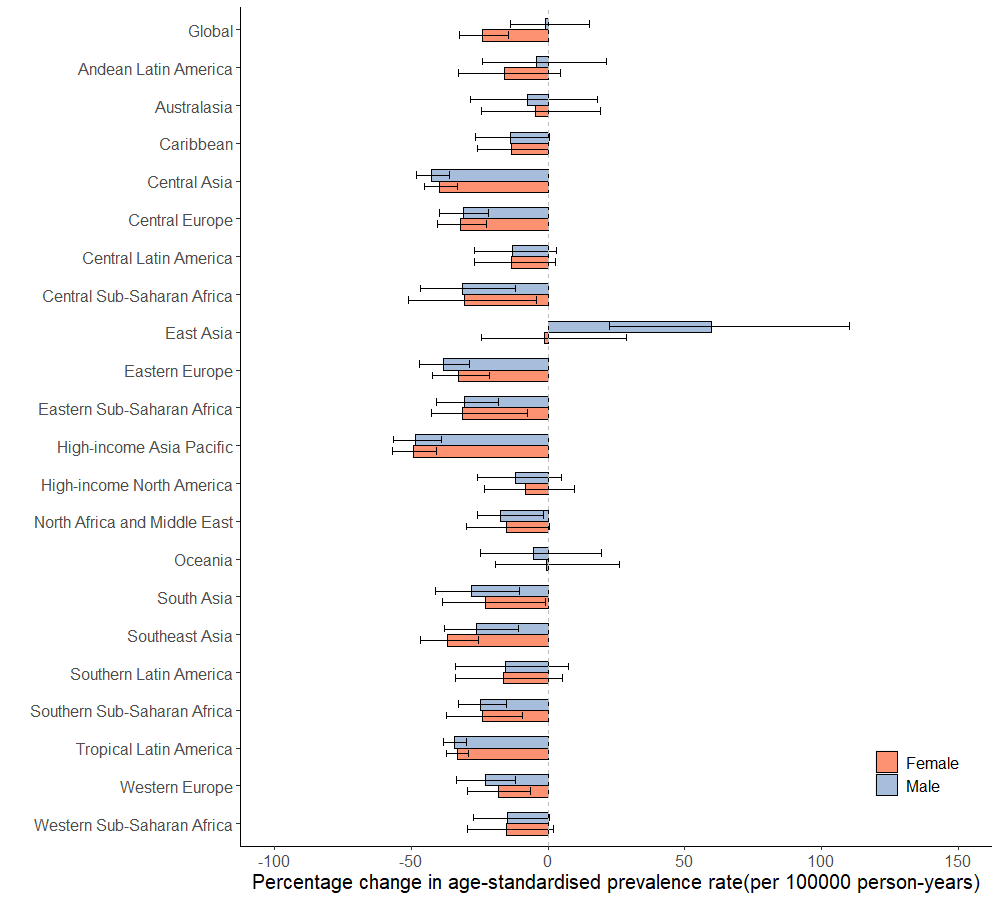


**Fig. S9. Death counts of stomach cancer for both sexes, in 2019.**


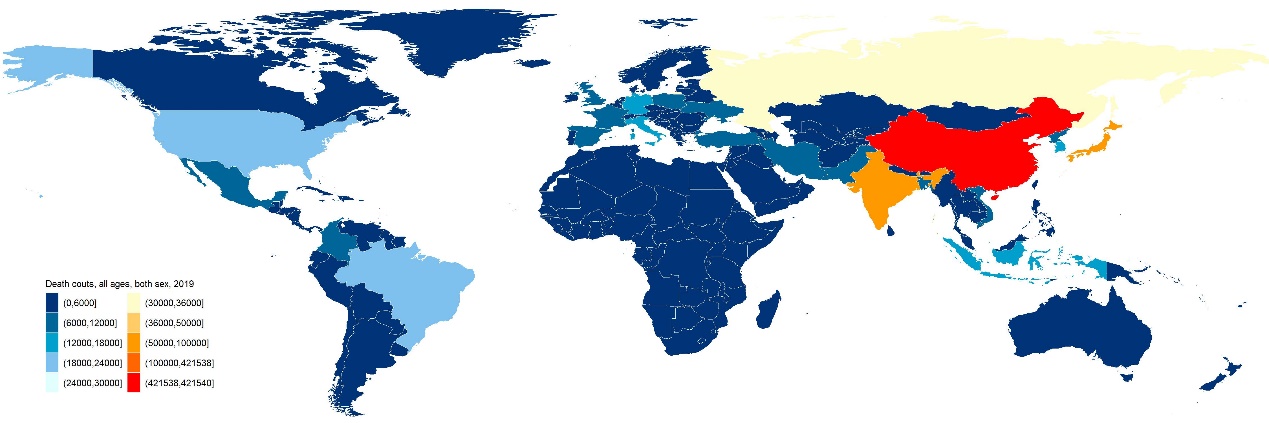


Maps were generated using R software (version 4.0.3) and ggplot2 package. [R Core Team (2019). R: A language and environment for statistical computing. R Foundation for Statistical Computing, Vienna, Austria. URL https://www.R-project.org; and (H. Wickham. ggplot2: Elegant Graphics for Data Analysis. Springer-Verlag New York, 2016. URL https:// https://ggplot2.tidyverse.org)].

**Fig. S10. Death counts of stomach cancer for 21 Global Burden Disease regions by sex, 2019.**


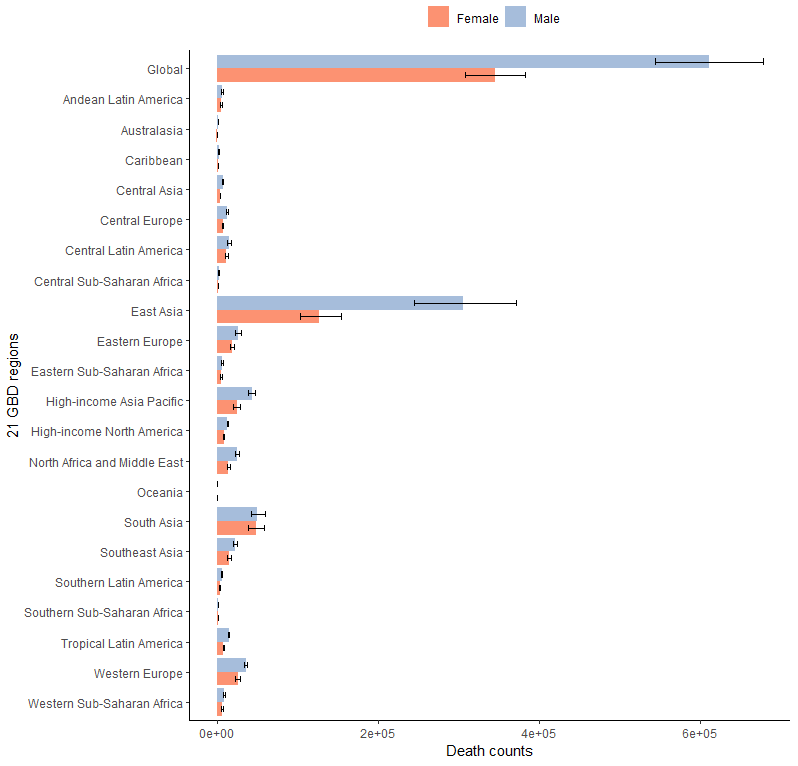


Error bars indicate the 95% uncertainty intervals (95% UI).

**Fig. S11. YLD counts of stomach cancer for both sexes, in 2019.**


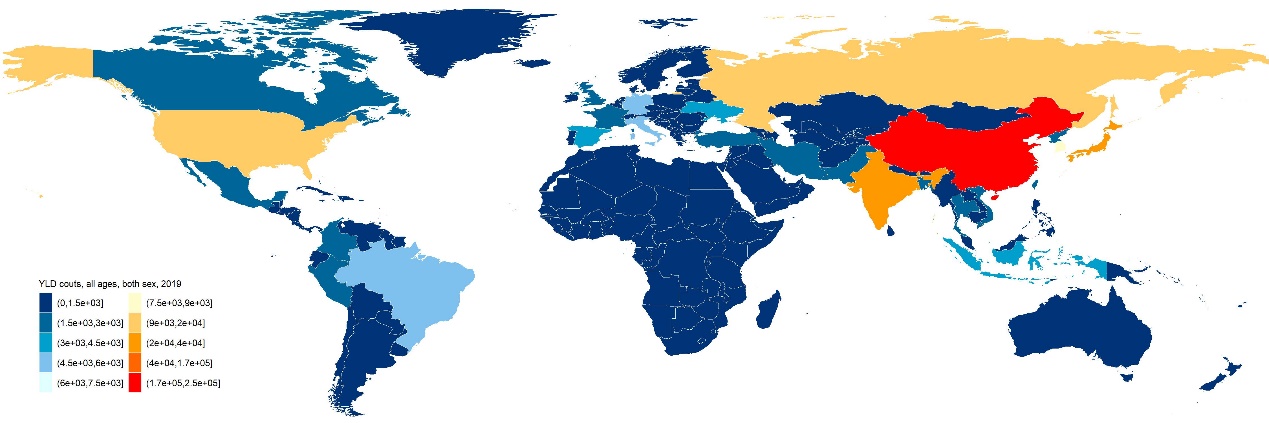


Maps were generated using R software (version 4.0.3) and ggplot2 package. [R Core Team (2019). R: A language and environment for statistical computing. R Foundation for Statistical Computing, Vienna, Austria. URL https://www.R-project.org; and (H. Wickham. ggplot2: Elegant Graphics for Data Analysis. Springer-Verlag New York, 2016. URL https:// https://ggplot2.tidyverse.org)].

**Fig. S12. Age-standardized YLD rate of stomach cancer per 100,000 person-years for both sexes, in 2019.**


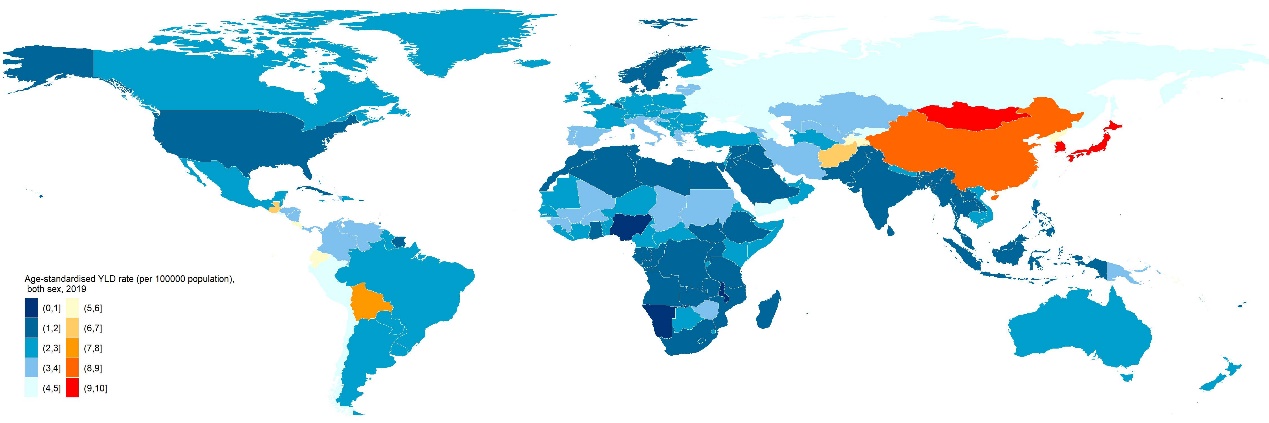


Maps were generated using R software (version 4.0.3) and ggplot2 package. [R Core Team (2019). R: A language and environment for statistical computing. R Foundation for Statistical Computing, Vienna, Austria. URL https://www.R-project.org; and (H. Wickham. ggplot2: Elegant Graphics for Data Analysis. Springer-Verlag New York, 2016. URL https:// https://ggplot2.tidyverse.org)].

**Fig. S13. Age-standardized YLD rates of stomach cancer per 100,000 person-years for 21 Global Burden Disease regions by sex, 2019.**


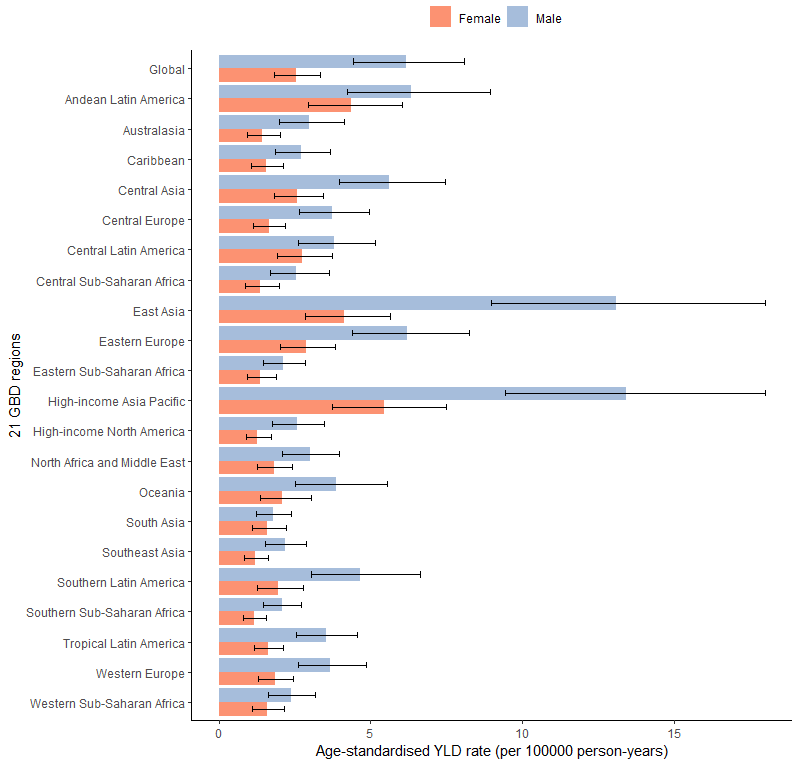


Error bars indicate the 95% uncertainty intervals (95% UI) for YLD. YLD=years lived with disability.

**Fig. S14. YLD counts for stomach cancer for 21 Global Burden Disease regions by sex, 2019.**


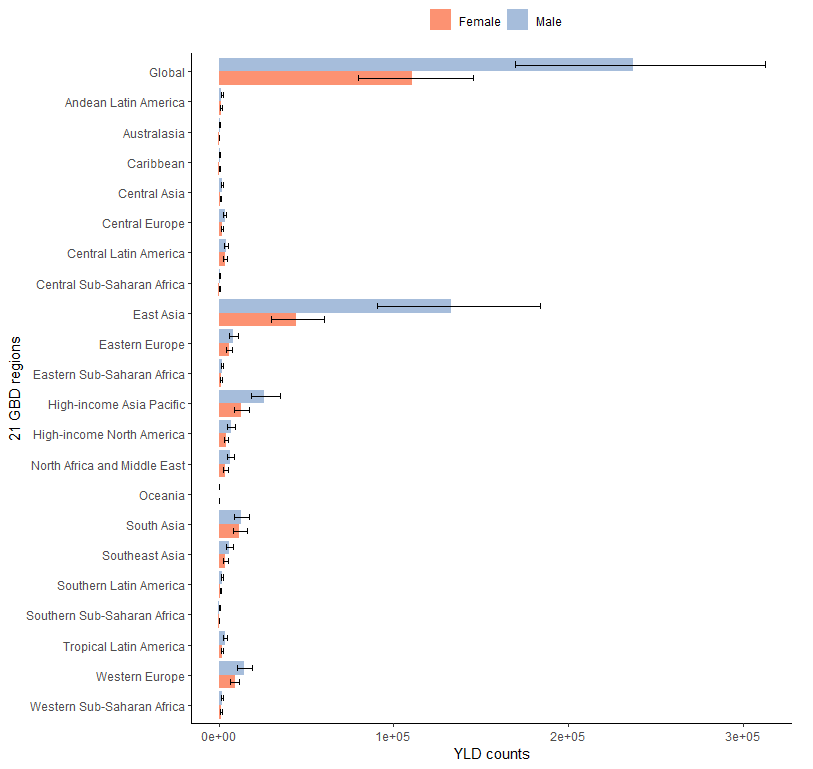


Error bars indicate the 95% uncertainty intervals (95% UI).

**Fig. S15. Global counts and age-standardized YLD rates of stomach cancer per 100,000 person-years by age and sex, 2019.**


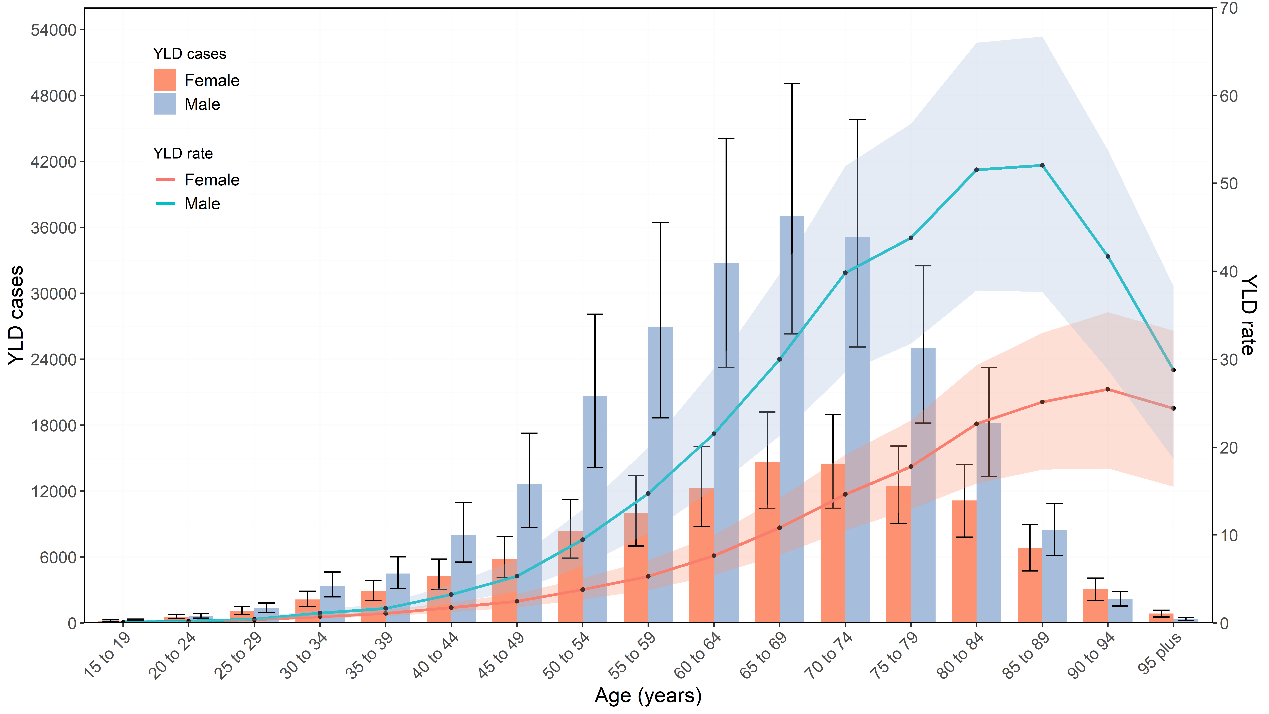


Error bars indicate the 95% uncertainty intervals (95% UI). Shading indicates the upper and lower limits of the 95% UI. YLDs=years lived with disability.

**Fig. S16. The percentage change in age-standardized YLD rates of stomach cancer per 100,000 person-years by sex for 21 Global Burden of Disease regions, 1990-2019.**

**
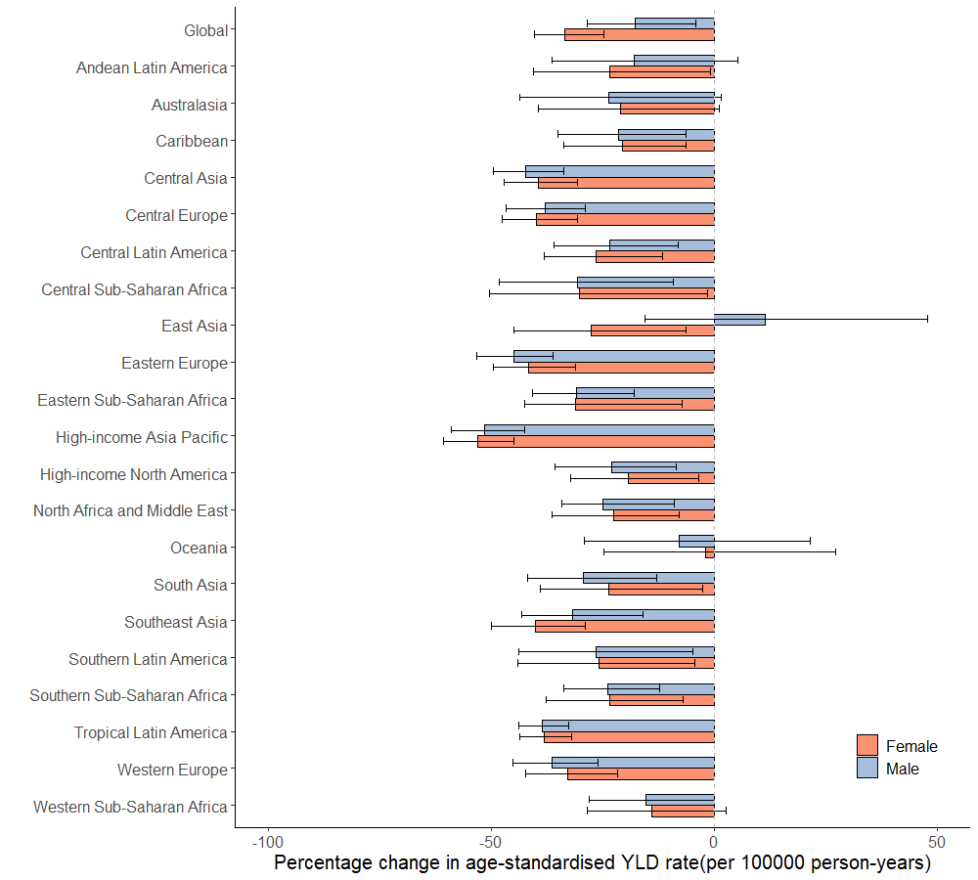
**

YLDs=years lived with disability.

**Fig. S17. Age-standardi**z**ed YLD rates for stomach cancer per 100,000 person-years for 204 countries and territories by SDI, 2019.**

**
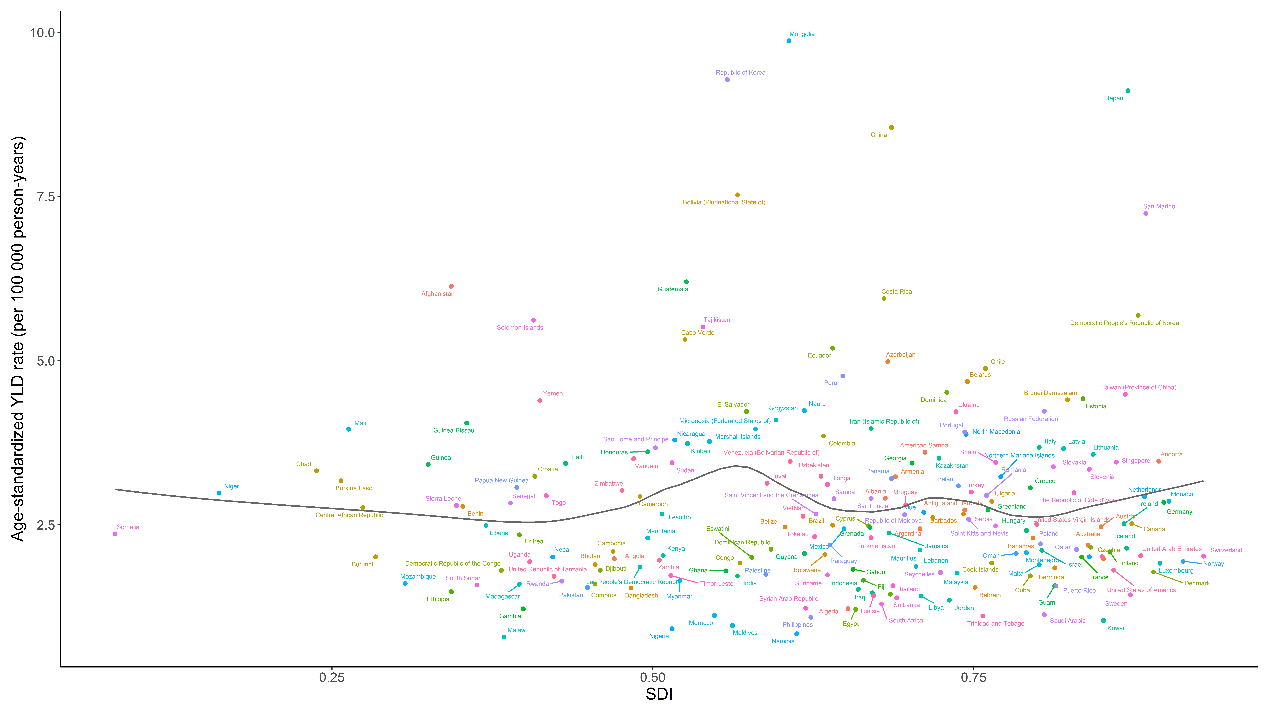
**

Black line represents the expected age-standardized YLD rates of stomach cancer based solely on SDI. SDI=Socio-demographic Index. YLD=years lived with disability

**Fig. S18. Age-standardized YLD rates for stomach cancer per 100,000 person-years for 21Global Burden of Disease region by SDI, 1990-2019.**


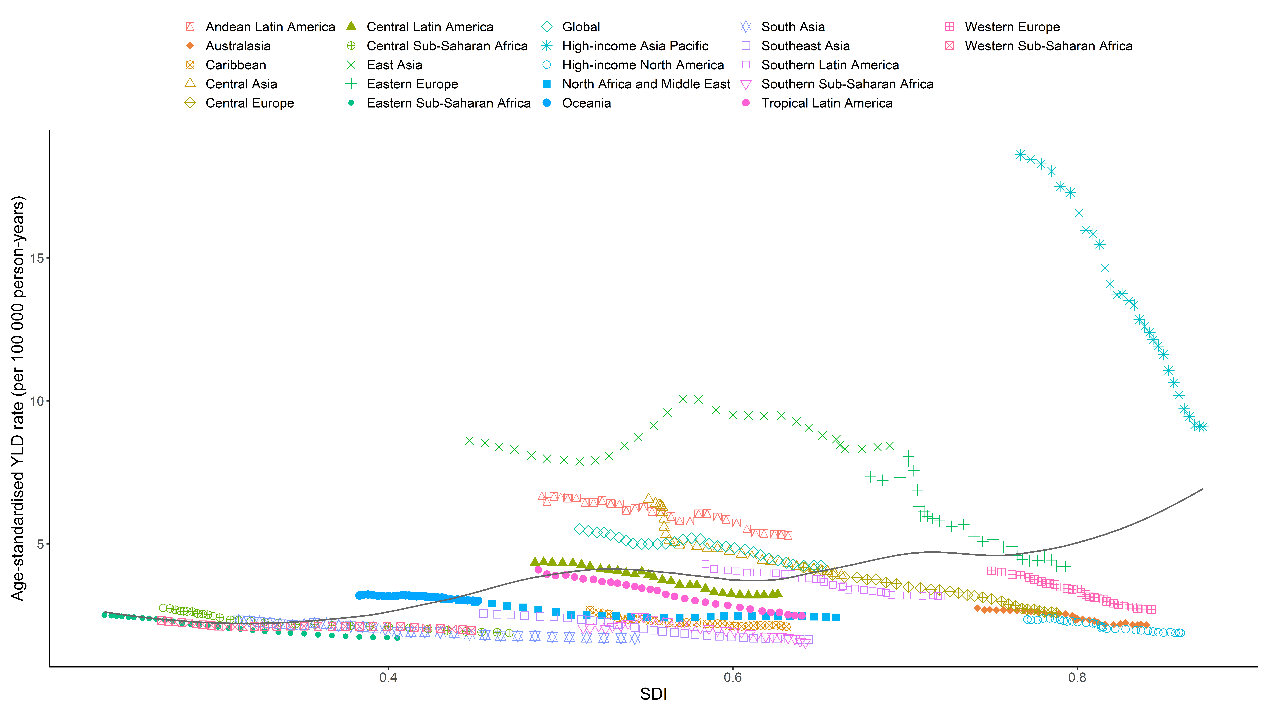


Black line represents the expected age-standardized YLDs rates of stomach cancer based solely on SDI. For each region, points from the left to right depict estimates from each year from 1990 to 2019. SDI=Socio-demographic Index. YLDs=years lived with disability

**Fig. S19. YLL counts of stomach cancer for both sexes, in 2019.**


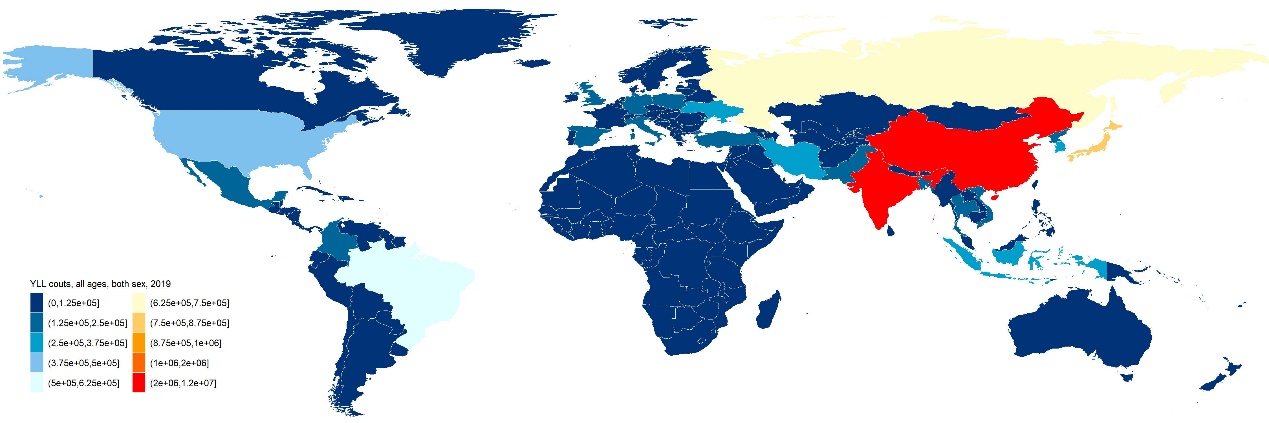


Maps were generated using R software (version 4.0.3) and ggplot2 package. [R Core Team (2019). R: A language and environment for statistical computing. R Foundation for Statistical Computing, Vienna, Austria. URL https://www.R-project.org; and (H. Wickham. ggplot2: Elegant Graphics for Data Analysis. Springer-Verlag New York, 2016. URL https:// https://ggplot2.tidyverse.org)].

**Fig. S20. Age-standardized YLL rate of stomach cancer per 100,000 person-years for both sexes, in 2019.**


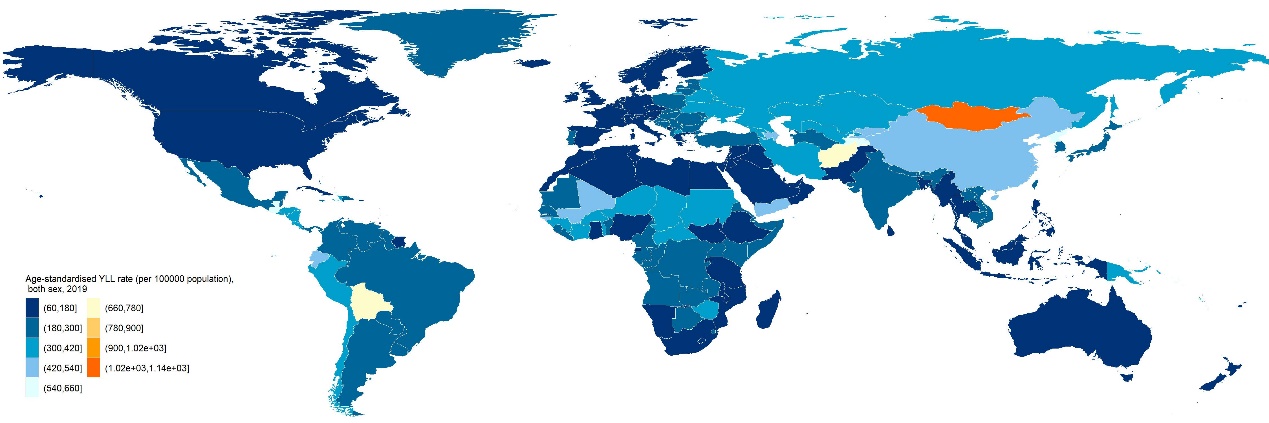


Maps were generated using R software (version 4.0.3) and ggplot2 package. [R Core Team (2019). R: A language and environment for statistical computing. R Foundation for Statistical Computing, Vienna, Austria. URL https://www.R-project.org; and (H. Wickham. ggplot2: Elegant Graphics for Data Analysis. Springer-Verlag New York, 2016. URL https:// https://ggplot2.tidyverse.org)].

**Fig. S21. Age-standardized YLL rates of stomach cancer per 100,000 person-years for 21 Global Burden Disease regions by sex, 2019.**


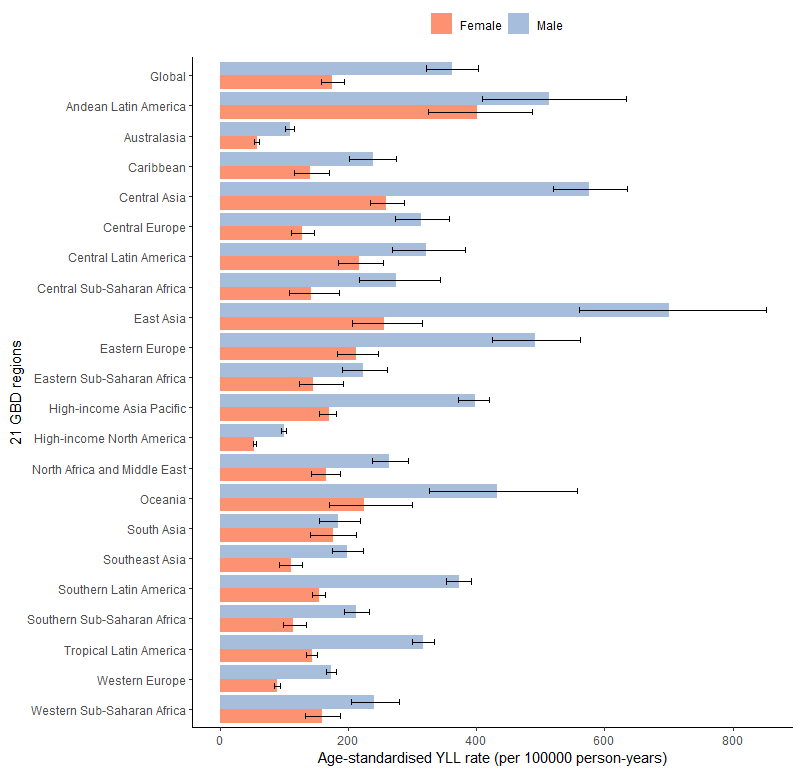


Error bars indicate the 95% uncertainty intervals (95% UI) for YLL. YLL=years of life lost.

**Fig. S22. YLL counts for stomach cancer for 21 Global Burden Disease regions by sex, 2019.**


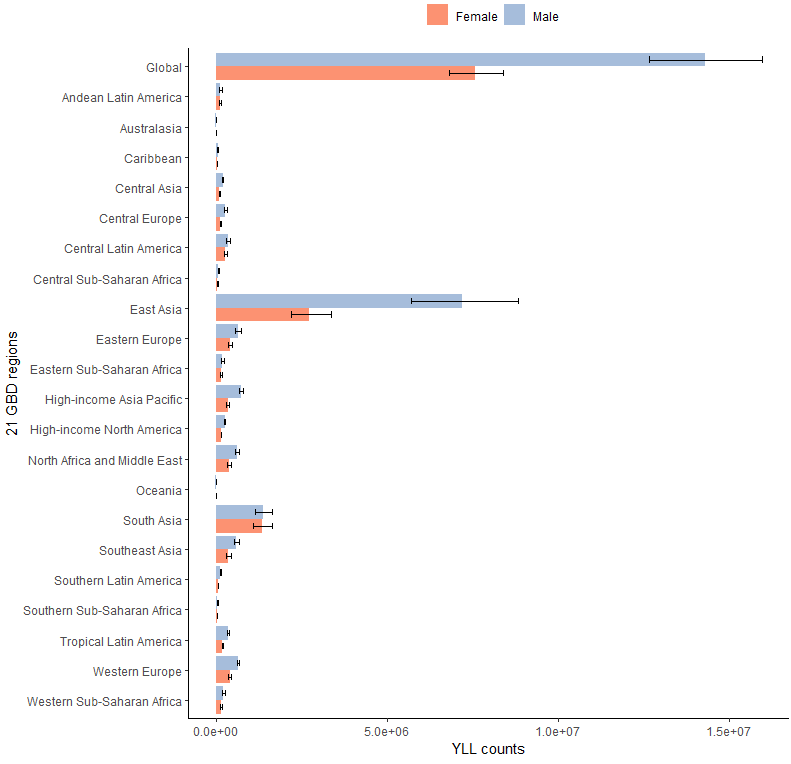


Error bars indicate the 95% uncertainty intervals (95% UI).

**Fig. S23. The percentage change in age-standardized YLL rates of stomach cancer per 100,000 person-years by sex for 21 Global Burden of Disease regions,1990-2019.**

**
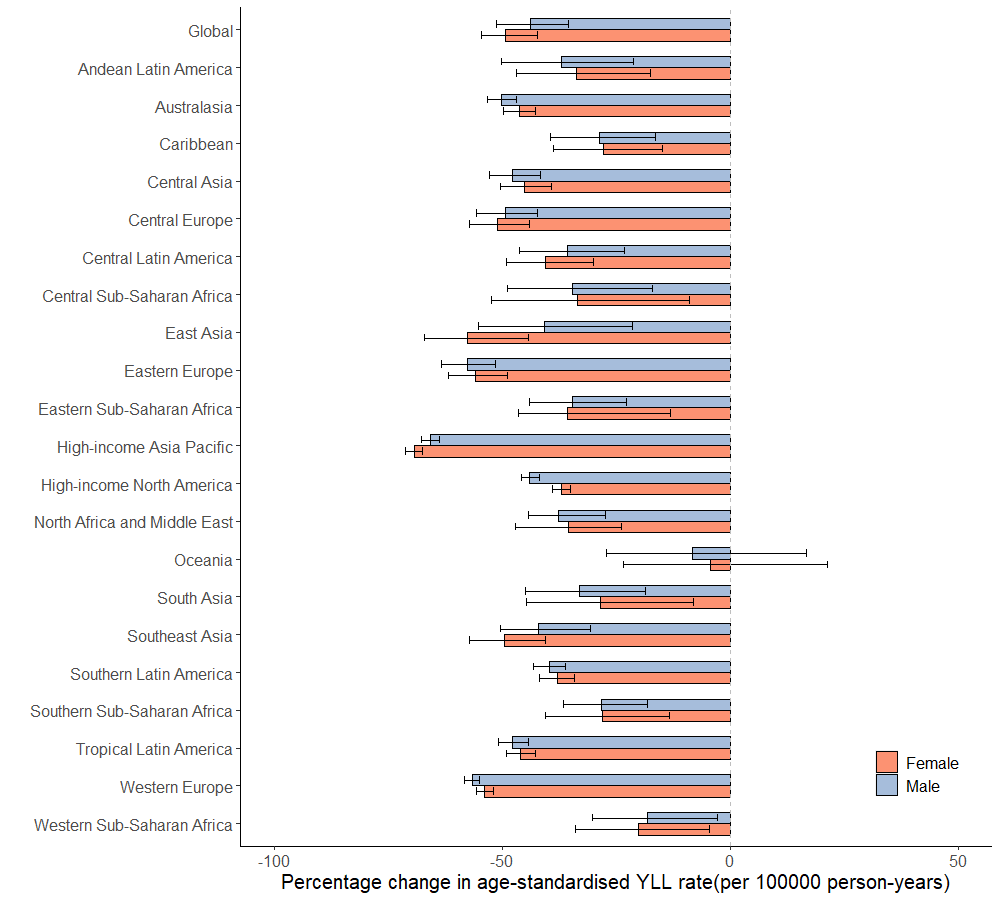
**

YLLs=years of life lost.

**Fig. S24. Age-standardized YLL rates for stomach cancer per 100,000 person-years for 204 countries and territories by SDI, 2019.**


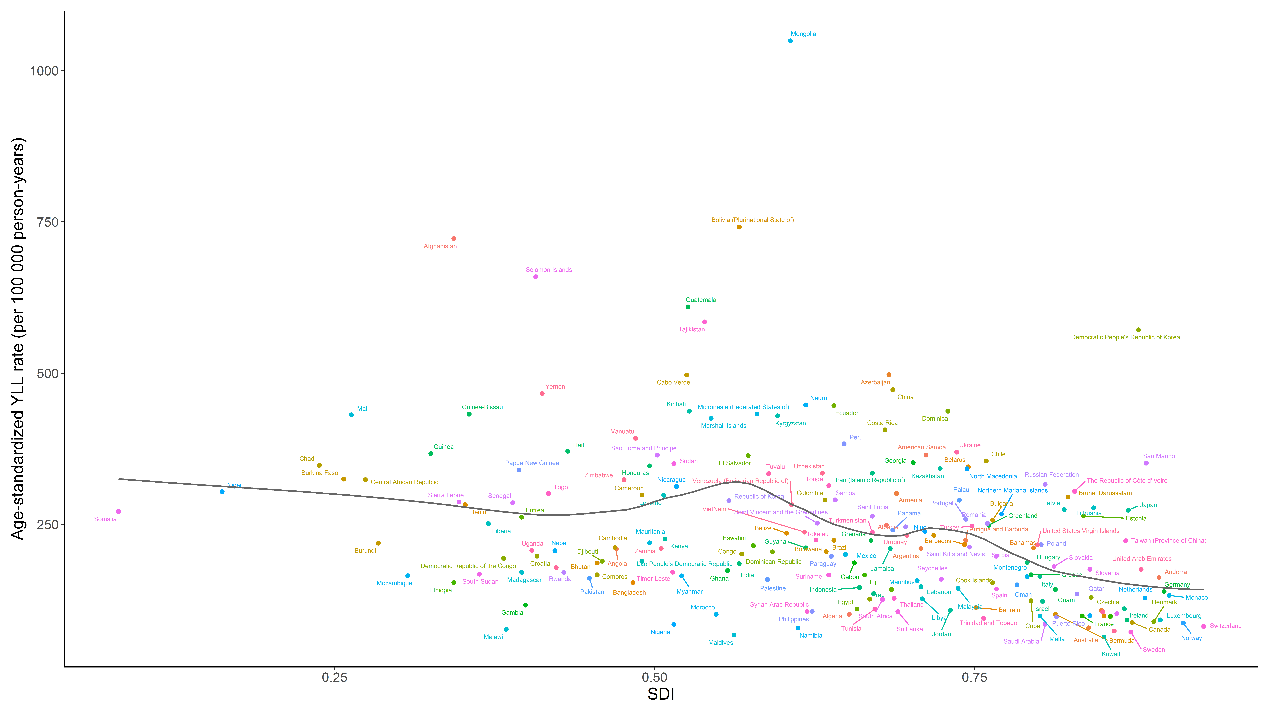


Black line represents the expected age-standardized YLL rates of stomach cancer based solely on SDI. SDI=Socio-demographic Index. YLL=years of life lost.

**Fig. S25. Age-standardized YLL rates for stomach cancer per 100,000 person-years for 21Global Burden of Disease regions by SDI, 1990-2019.**


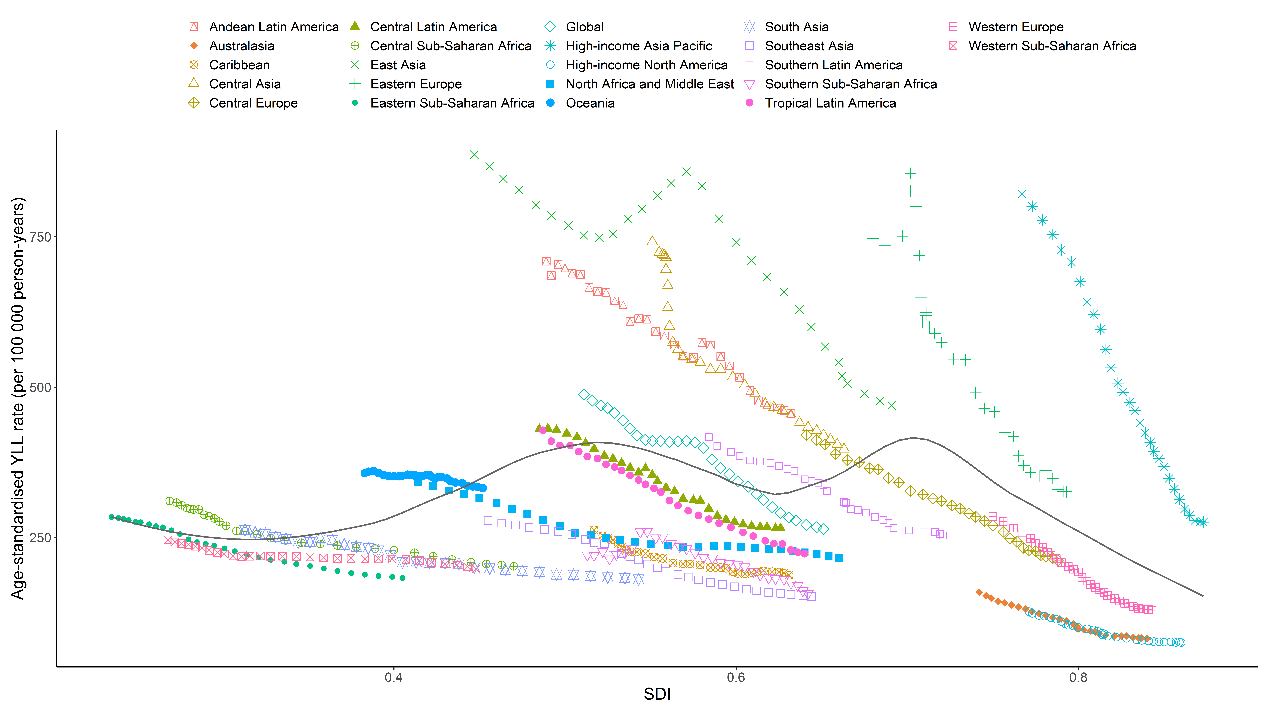


Black line represents the expected age-standardized YLL rates of stomach cancer based solely on SDI. For each region, points from the left to right depict estimates from each year from 1990 to 2019. SDI=Socio-demographic Index. YLL= years of life lost.

**Fig. S26. DALY counts of stomach cancer for both sexes, in 2019.**


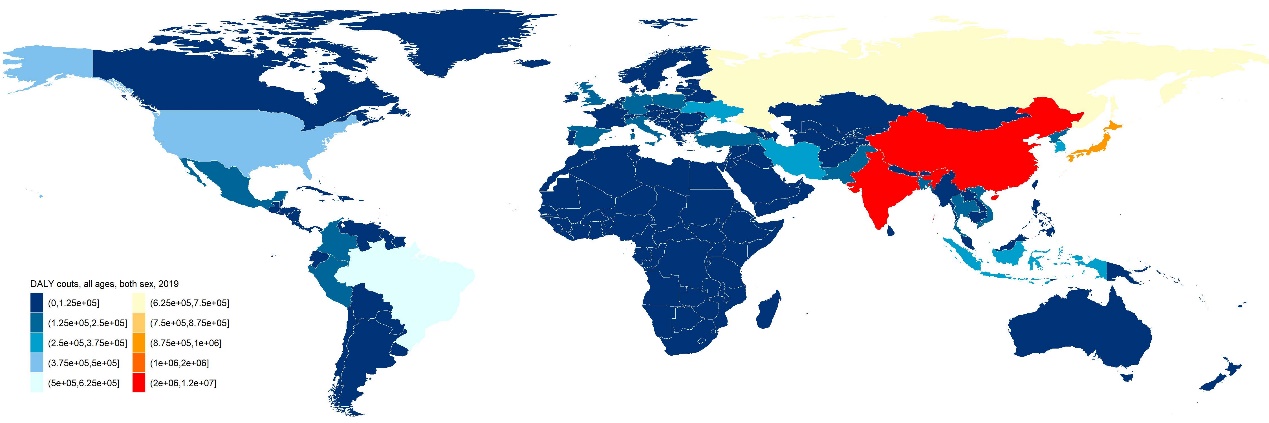


Maps were generated using R software (version 4.0.3) and ggplot2 package. [R Core Team (2019). R: A language and environment for statistical computing. R Foundation for Statistical Computing, Vienna, Austria. URL https://www.R-project.org; and (H. Wickham. ggplot2: Elegant Graphics for Data Analysis. Springer-Verlag New York, 2016. URL https:// https://ggplot2.tidyverse.org)].

**Fig. S27. Age-standardized DALY rate of stomach cancer per 100,000 person-years for both sexes, in 2019.**


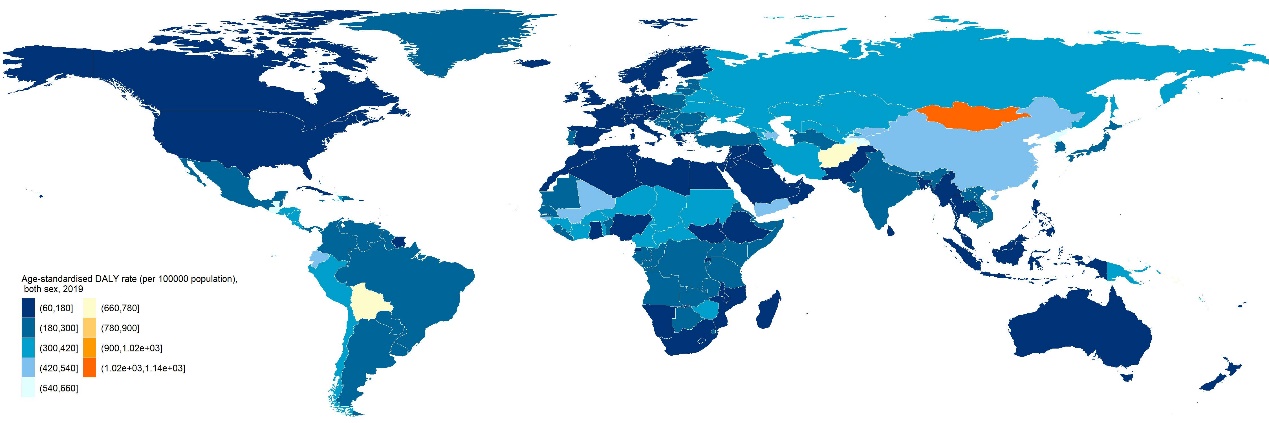


Maps were generated using R software (version 4.0.3) and ggplot2 package. [R Core Team (2019). R: A language and environment for statistical computing. R Foundation for Statistical Computing, Vienna, Austria. URL https://www.R-project.org; and (H. Wickham. ggplot2: Elegant Graphics for Data Analysis. Springer-Verlag New York, 2016. URL https:// https://ggplot2.tidyverse.org)].

**Fig. S28. The 204 countries and territories distribution by SDI quintile, 2019.**


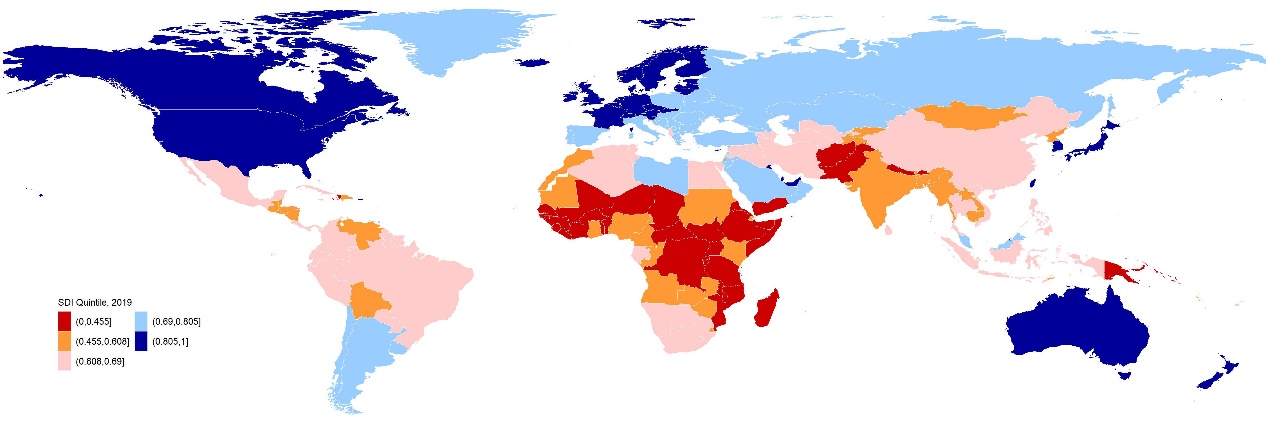


SDI= Socio-Demographic Index. Maps were generated using R software (version 4.0.3) and ggplot2 package. [R Core Team (2019). R: A language and environment for statistical computing. R Foundation for Statistical Computing, Vienna, Austria. URL https://www.R-project.org; and (H. Wickham. ggplot2: Elegant Graphics for Data Analysis. Springer-Verlag New York, 2016. URL https:// https://ggplot2.tidyverse.org)].

**Fig. S29. DALY counts for stomach cancer for 21 Global Burden Disease regions by sex, 2019.**


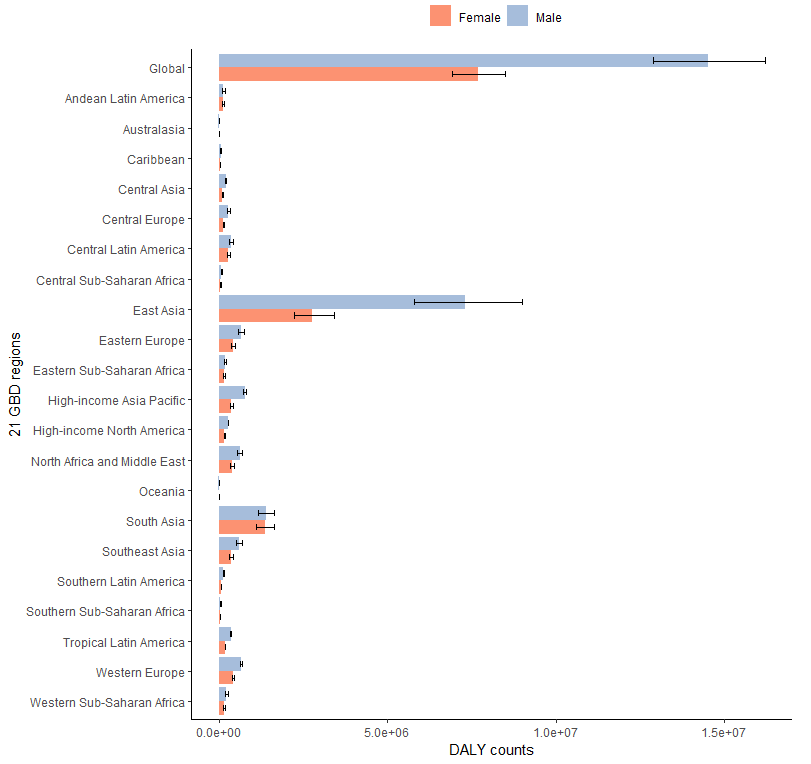


Error bars indicate the 95% uncertainty intervals (95% UI).

**Fig. S30. Proportion of stomach cancer DALYs attributable to smoking (A, female, B, male), diet high in sodium (C, female, D, male) by sex, 2019.**


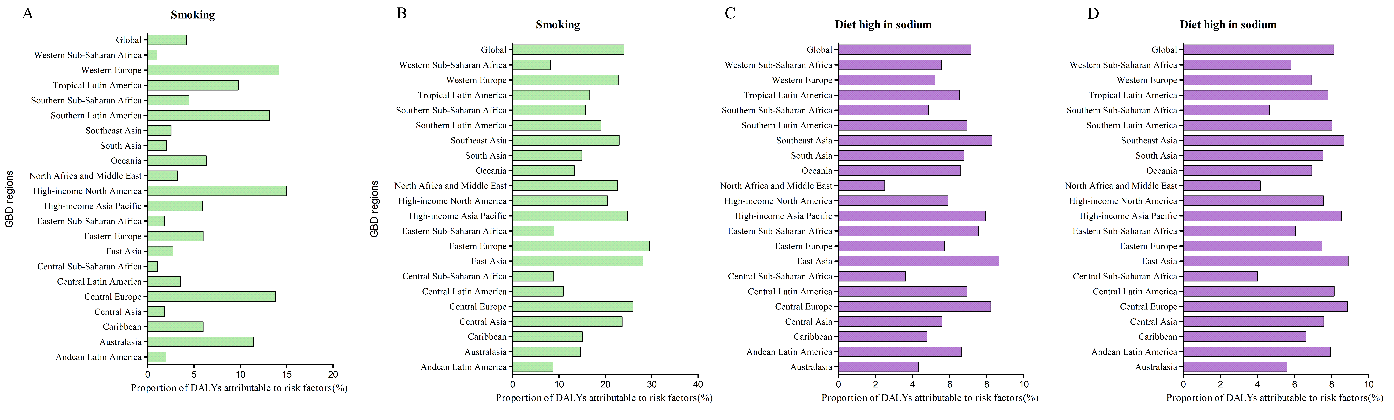


DALYs=disability adjusted life years.

**Fig. S31. The age-standardized rate of incidence, death and DALY in different levels of SDI countries,both sexes, 2019.**
