# Supplementary material for: The global, regional and national burden of stomach cancer and its attributable risk factors from 1990 to 2019
Source: Sci Rep. 2022 Jul 7;12:11542. doi: 10.1038/s41598-022-15839-7 (PMC9262989; doi:10.1038/s41598-022-15839-7)
Supplement: Supplementary file 3 — Supplementary Tables. [file 41598_2022_15839_MOESM3_ESM.docx]

The global, regional and national burden of stomach cancer and its attributable risk factors from 1990 to 2019

Yexun Song^1^, Xiajing Liu^2^, Wenwei Cheng^1,3^, Heqing Li^1^, Decai Zhang^4, 5^

1 Department of Otolaryngology-Head Neck Surgery, The Third Xiangya Hospital of Central South University, Changsha, 410013, Hunan Province, China.

2 Graduate School of Guilin Medical University, Guilin, 541004, Guangxi Province, China.

3 Xiangya School of Public Health, Central South University, Changsha, 410000, Hunan Province, China.

4 Department of Gastroenterology, The Third Xiangya Hospital of Central South University, Changsha 410013, Hunan Province, China.

5 Hunan Key Laboratory of Nonresolving Inflammation and Cancer, Changsha 410013, Hunan Province, China

Correspondence: Decai Zhang, Department of Gastroenterology, The Third Xiangya Hospital of Central South University, Changsha 410013, Hunan Province, China. Email: decaizhang@csu.edu.cn

Supplementary Table 1 The prevalence, YLLs and YLDs for stomach cancer in 2019 for both sexes, and percentage change of age-standardised rates (ASRs) by Global Disease Burden region, 1990-2019

|  | YLLs (95%Uncertainty Interval) | | | YLDs (95%Uncertainty Interval) | | |
| --- | --- | --- | --- | --- | --- | --- |
|  | Counts | ASR per 100,000 population (95%UI) | Percentage change in ASRs per 100,000 population(95%UI) | Numbers | ASR per 100,000 population (95%UI) | Percentage change in ASRs per 100,000 population (95%UI) |
| Global | 21872432 (19972705 to 23712517) | 264.2 (241.5 to 286) | -45.9 (-51.1 to -40) | 348549 (252331 to 457637) | 4.2 (3.1 to 5.6) | -23.1 (-30.6 to -14) |
| Andean Latin America | 260082 (210229 to 317158) | 455.9 (369.5 to 555.5) | -35.7 (-48.6 to -20.4) | 2946 (1984 to 4123) | 5.3 (3.6 to 7.4) | -20.7 (-36.7 to -0.6) |
| Australasia | 37869 (35074 to 40395) | 82.3 (76.8 to 87.6) | -48.2 (-51.1 to -45) | 1037 (699 to 1444) | 2.2 (1.4 to 3) | -21.3 (-39.2 to 0.5) |
| Caribbean | 96615 (81981 to 111939) | 187.4 (158.8 to 217.1) | -28.7 (-38.9 to -17.2) | 1087 (745 to 1477) | 2.1 (1.4 to 2.9) | -21.5 (-33.3 to -8.4) |
| Central Asia | 322002 (290988 to 356927) | 397 (360.5 to 438.3) | -46.5 (-51.6 to -40.7) | 2969 (2084 to 3967) | 3.9 (2.8 to 5.2) | -40.8 (-47.4 to -33.5) |
| Central Europe | 422961 (370371 to 476359) | 212.5 (185.3 to 240.1) | -49.5 (-55.7 to -43.3) | 5338 (3802 to 7013) | 2.6 (1.8 to 3.4) | -38.3 (-46.2 to -29.8) |
| Central Latin America | 640178 (545363 to 754108) | 265.1 (226 to 311.8) | -38.3 (-47.3 to -26.9) | 7692 (5353 to 10290) | 3.2 (2.2 to 4.3) | -25.4 (-36.4 to -11.2) |
| Central Sub-Saharan Africa | 124446 (97010 to 156541) | 202.2 (161.6 to 251.2) | -34.9 (-49.2 to -18.1) | 1053 (699 to 1512) | 1.9 (1.3 to 2.7) | -31.8 (-48.6 to -12.3) |
| East Asia | 9925742 (8292104 to 11718757) | 469.5 (393.8 to 551.7) | -47 (-56.9 to -34.8) | 177039 (124502 to 238367) | 8.4 (6 to 11.4) | -2 (-20.7 to 22.8) |
| Eastern Europe | 1067264 (959819 to 1188288) | 326.2 (293.1 to 362.6) | -56.4 (-60.8 to -51.7) | 13969 (10074 to 18091) | 4.2 (3 to 5.5) | -42.7 (-49 to -35.7) |
| Eastern Sub-Saharan Africa | 342865 (295499 to 396596) | 182.7 (159.4 to 208.8) | -35.7 (-43.1 to -25.9) | 2913 (2041 to 3908) | 1.7 (1.2 to 2.3) | -31.7 (-39.4 to -22.1) |
| High-income Asia Pacific | 1122336 (1010283 to 1191689) | 275.6 (255.5 to 290) | -66.4 (-68.2 to -64.8) | 39013 (27739 to 51477) | 9.1 (6.5 to 12.1) | -51.1 (-57.4 to -44.3) |
| High-income North America | 434862 (416067 to 449225) | 75.5 (72.8 to 77.9) | -40.5 (-42.2 to -38.7) | 11327 (7974 to 14790) | 1.9 (1.3 to 2.5) | -20 (-30.2 to -7.9) |
| North Africa and Middle East | 1000919 (895990 to 1120114) | 215.7 (194.6 to 239.8) | -37 (-44.2 to -27.2) | 10531 (7297 to 13915) | 2.4 (1.7 to 3.2) | -23.9 (-32.7 to -10.5) |
| Oceania | 28437 (21413 to 36409) | 332 (253 to 418.7) | -7 (-24.1 to 14.6) | 232 (150 to 336) | 3 (2 to 4.3) | -5.8 (-24.3 to 18.8) |
| South Asia | 2749980 (2392190 to 3140356) | 180.4 (157 to 205.7) | -31.3 (-41.1 to -20.3) | 24533 (17249 to 32668) | 1.7 (1.2 to 2.2) | -27.2 (-38 to -14.5) |
| Southeast Asia | 977155 (866737 to 1093819) | 151.9 (135.2 to 169.8) | -45.4 (-52.2 to -36.7) | 10166 (7173 to 13496) | 1.7 (1.2 to 2.2) | -35.5 (-44.6 to -24) |
| Southern Latin America | 206655 (195773 to 217357) | 253.3 (240.5 to 266.2) | -39.2 (-42.4 to -36.1) | 2614 (1724 to 3683) | 3.2 (2.1 to 4.5) | -26.4 (-42.9 to -6.1) |
| Southern Sub-Saharan Africa | 94838 (85733 to 105614) | 156.9 (142.9 to 173.2) | -29 (-37.1 to -19.8) | 883 (630 to 1171) | 1.6 (1.1 to 2) | -24.7 (-33.6 to -14.7) |
| Tropical Latin America | 550675 (524152 to 575267) | 223.2 (212.1 to 233.2) | -47.9 (-50.3 to -45.1) | 6038 (4342 to 7764) | 2.5 (1.8 to 3.2) | -39.3 (-43.5 to -34.8) |
| Western Europe | 1073884 (1005119 to 1123720) | 129.3 (122.8 to 134.9) | -54.6 (-56.2 to -53.1) | 23602 (16809 to 30877) | 2.7 (1.9 to 3.6) | -33.3 (-41.9 to -22.8) |
| Western Sub-Saharan Africa | 392666 (334035 to 459223) | 197.7 (169.9 to 227.5) | -19.6 (-30.9 to -6.7) | 3565 (2454 to 4827) | 2 (1.4 to 2.6) | -15.1 (-26.7 to -2) |

YLLs=years of life lost, YLDs=years lived with disability.

Supplementary Table 2 The incidence, death and YLDs for stomach cancer for both sexes by Global Disease Burden region in 1990

|  | Incidence (95%Uncertainty Interval) | | Death (95%Uncertainty Interval) | | YLDs (95%Uncertainty Interval) | |
| --- | --- | --- | --- | --- | --- | --- |
|  | Counts | ASR per 100,000 population (95%UI) | Counts | ASR per 100,000 population (95%UI) | Counts | ASR per 100,000 population (95%UI) |
| Global | 883396 (834237 to 929174) | 22.4 (21.2 to 23.6) | 788317 (742787 to 833999) | 20.5 (19.2 to 21.6) | 221339 (161074 to 283086) | 5.5 (4 to 7) |
| Andean Latin America | 5976 (5439 to 6539) | 29.7 (26.9 to 32.4) | 6202 (5649 to 6788) | 31.7 (28.9 to 34.6) | 1382 (959 to 1809) | 6.7 (4.7 to 8.7) |
| Australasia | 2382 (2277 to 2476) | 10.2 (9.7 to 10.6) | 1762 (1680 to 1824) | 7.6 (7.2 to 7.9) | 643 (468 to 835) | 2.7 (2 to 3.6) |
| Caribbean | 2924 (2668 to 3104) | 11.3 (10.3 to 12) | 2933 (2671 to 3109) | 11.5 (10.5 to 12.2) | 702 (510 to 902) | 2.7 (1.9 to 3.4) |
| Central Asia | 13392 (12977 to 13762) | 28 (27.1 to 28.8) | 13183 (12771 to 13540) | 28.1 (27.2 to 28.9) | 3189 (2277 to 4066) | 6.6 (4.7 to 8.4) |
| Central Europe | 26457 (25809 to 26921) | 18.1 (17.6 to 18.4) | 26330 (25633 to 26805) | 18.2 (17.6 to 18.5) | 6144 (4455 to 7849) | 4.2 (3 to 5.3) |
| Central Latin America | 15496 (14958 to 15890) | 19 (18.2 to 19.6) | 15448 (14844 to 15861) | 19.7 (18.7 to 20.3) | 3663 (2696 to 4710) | 4.3 (3.2 to 5.6) |
| Central Sub-Saharan Africa | 2725 (2212 to 3295) | 11.9 (9.9 to 14.1) | 2754 (2296 to 3258) | 12.7 (10.8 to 14.9) | 660 (443 to 926) | 2.8 (1.9 to 3.8) |
| East Asia | 325714 (285493 to 367285) | 37.1 (32.7 to 41.7) | 313103 (274773 to 353489) | 37.2 (32.8 to 41.7) | 77888 (55530 to 103753) | 8.6 (6.1 to 11.4) |
| Eastern Europe | 87033 (84187 to 88711) | 30.9 (29.8 to 31.5) | 80930 (78368 to 82540) | 28.9 (27.9 to 29.5) | 20674 (14990 to 26243) | 7.3 (5.3 to 9.3) |
| Eastern Sub-Saharan Africa | 8234 (7145 to 9172) | 10.7 (9.3 to 11.8) | 8336 (7252 to 9302) | 11.3 (9.9 to 12.6) | 2005 (1400 to 2645) | 2.5 (1.7 to 3.3) |
| High-income Asia Pacific | 123732 (119612 to 126500) | 61.5 (59.3 to 63) | 69647 (66944 to 71223) | 35.5 (34 to 36.4) | 37860 (28094 to 48657) | 18.6 (13.8 to 23.9) |
| High-income North America | 30319 (29085 to 31116) | 8.5 (8.2 to 8.7) | 21405 (20332 to 22008) | 6 (5.7 to 6.1) | 8282 (6045 to 10537) | 2.4 (1.7 to 3) |
| North Africa and Middle East | 23485 (20470 to 25671) | 13.7 (11.9 to 15) | 23545 (20398 to 25760) | 14.4 (12.5 to 15.8) | 5652 (4019 to 7312) | 3.2 (2.3 to 4.1) |
| Oceania | 420 (327 to 512) | 13.8 (11 to 16.6) | 410 (321 to 500) | 14.5 (11.6 to 17.4) | 103 (68 to 145) | 3.2 (2.2 to 4.4) |
| South Asia | 57574 (51984 to 63221) | 9.9 (8.9 to 11) | 57248 (51441 to 63010) | 10.5 (9.4 to 11.5) | 14057 (9872 to 18330) | 2.3 (1.6 to 3) |
| Southeast Asia | 28069 (24305 to 31091) | 10.9 (9.5 to 12.1) | 28206 (24571 to 31057) | 11.5 (10 to 12.6) | 6843 (4886 to 8982) | 2.6 (1.8 to 3.4) |
| Southern Latin America | 8456 (8190 to 8698) | 18.5 (17.9 to 19.1) | 8520 (8222 to 8766) | 19 (18.2 to 19.5) | 1973 (1418 to 2537) | 4.3 (3.1 to 5.5) |
| Southern Sub-Saharan Africa | 2419 (2198 to 2631) | 8.7 (7.9 to 9.5) | 2452 (2230 to 2665) | 9.2 (8.3 to 10.1) | 588 (414 to 777) | 2.1 (1.5 to 2.7) |
| Tropical Latin America | 16110 (15503 to 16604) | 18.1 (17.2 to 18.7) | 16203 (15490 to 16720) | 18.9 (17.9 to 19.5) | 3772 (2717 to 4834) | 4.1 (3 to 5.2) |
| Western Europe | 93952 (90016 to 96137) | 16.1 (15.5 to 16.5) | 80783 (77139 to 82719) | 13.7 (13.1 to 14.1) | 23243 (17071 to 29522) | 4.1 (3 to 5.2) |
| Western Sub-Saharan Africa | 8528 (7401 to 9620) | 10.2 (8.9 to 11.4) | 8917 (7789 to 10038) | 11.1 (9.7 to 12.5) | 2014 (1401 to 2690) | 2.3 (1.6 to 3.1) |

DALYs=disability adjusted life years, SDI=Socio-demographic Index.

Supplementary Table 3 The prevalence, YLLs and DALYs for stomach cancer for both sexes by Global Disease Burden region in 1990

|  | YLLs (95%Uncertainty Interval) | | DALYs (95%Uncertainty Interval) | |
| --- | --- | --- | --- | --- |
|  | Counts | ASR per 100,000 population (95%UI) | Counts | ASR per 100,000 population (95%UI) |
| Global | 20241688 (19030222 to 21513162) | 487.9 (459 to 518) | 20463027 (19234882 to 21737495) | 493.4 (463.7 to 523.7) |
| Andean Latin America | 155094 (141188 to 170105) | 709.3 (645.5 to 775.6) | 156476 (142491 to 171497) | 716 (650.8 to 783.7) |
| Australasia | 36903 (35609 to 37989) | 159 (153.4 to 163.7) | 37546 (36252 to 38675) | 161.8 (156.2 to 166.6) |
| Caribbean | 70471 (63208 to 75432) | 263 (236.6 to 281.4) | 71173 (63860 to 76225) | 265.6 (239 to 284.4) |
| Central Asia | 371155 (360048 to 381495) | 741.5 (718.9 to 762.3) | 374345 (362963 to 384623) | 748.1 (724.8 to 769.2) |
| Central Europe | 621237 (608071 to 631185) | 420.7 (411.4 to 427.4) | 627381 (613872 to 637669) | 424.8 (415.5 to 431.7) |
| Central Latin America | 384730 (373672 to 393293) | 430 (416.6 to 440.4) | 388393 (376945 to 397123) | 434.3 (420.6 to 444.8) |
| Central Sub-Saharan Africa | 80725 (66089 to 97079) | 310.8 (259.2 to 368.1) | 81385 (66714 to 97903) | 313.6 (261.1 to 371.4) |
| East Asia | 8388981 (7310840 to 9495407) | 886.4 (775.4 to 1000.8) | 8466869 (7396546 to 9577275) | 895 (784.8 to 1009.8) |
| Eastern Europe | 2102662 (2026422 to 2144963) | 747.6 (719 to 762.8) | 2123336 (2045469 to 2165630) | 755 (725.8 to 770.4) |
| Eastern Sub-Saharan Africa | 245209 (210558 to 275285) | 284 (246.8 to 317.5) | 247214 (212393 to 277389) | 286.5 (249.2 to 320.4) |
| High-income Asia Pacific | 1674580 (1630712 to 1704883) | 821.1 (798.6 to 836.3) | 1712439 (1668468 to 1744699) | 839.7 (816.8 to 856) |
| High-income North America | 433089 (420399 to 441314) | 126.9 (123.4 to 129.3) | 441372 (427965 to 450276) | 129.3 (125.6 to 131.8) |
| North Africa and Middle East | 648017 (562249 to 711493) | 342.3 (295.8 to 375.6) | 653668 (567326 to 717359) | 345.5 (298.3 to 379.5) |
| Oceania | 12752 (9854 to 15678) | 357.1 (279.8 to 436) | 12855 (9948 to 15831) | 360.3 (282 to 441.1) |
| South Asia | 1731712 (1550139 to 1903443) | 262.6 (235.4 to 288.9) | 1745769 (1561472 to 1915358) | 264.9 (237.5 to 291.5) |
| Southeast Asia | 793125 (691149 to 872059) | 277.9 (242.4 to 305.6) | 799968 (696908 to 879257) | 280.5 (244.7 to 308.6) |
| Southern Latin America | 193912 (188633 to 199236) | 416.8 (405.3 to 428) | 195885 (190337 to 201197) | 421.1 (409.3 to 432.4) |
| Southern Sub-Saharan Africa | 67336 (61704 to 72836) | 221 (202.4 to 239.7) | 67924 (62300 to 73461) | 223 (204.3 to 241.6) |
| Tropical Latin America | 417282 (401874 to 429930) | 428.3 (411 to 441.6) | 421054 (405347 to 433822) | 432.4 (414.9 to 446.1) |
| Western Europe | 1583882 (1535974 to 1610382) | 285 (277.3 to 289.6) | 1607126 (1557221 to 1636772) | 289.1 (280.9 to 294.2) |
| Western Sub-Saharan Africa | 228833 (197855 to 261112) | 246.1 (215 to 279.1) | 230847 (199588 to 263341) | 248.4 (217.2 to 281.7) |

YLDs=years lived with disability, YLLs=years of life lost, SDI=Socio-demographic Index.

Supplementary Table 4 The incidence, death and DALYs for stomach cancer for both sex in 2019, and percentage change of age-standardised rates (ASRs) by 204 countries, 1990-2019

|  | Incidence (95% Uncertainty Interval) | | | Death (95% Uncertainty Interval) | | | DALYs (95% Uncertainty Interval) | | |
| --- | --- | --- | --- | --- | --- | --- | --- | --- | --- |
|  | Counts | ASR per 100,000 population (95%UI) | Percentage change in ASRs per 100,000 population (95%UI) | Counts | ASR per 100,000 population (95%UI) | Percentage change in ASRs per 100,000 population(95%UI) | Numbers | ASR per 100,000 population (95%UI) | Percentage change in ASRs per 100,000 population (95%UI) |
| Afghanistan | 3672 (2527 to 4816) | 27.7 (19.9 to 34.9) | -8.9 (-30.5 to 16.4) | 3642 (2527 to 4721) | 29.3 (21.2 to 36.5) | -8.4 (-29.5 to 16) | 115419 (75037 to 157492) | 728.7 (505.3 to 939.4) | -12 (-34.1 to 15.5) |
| Albania | 489 (370 to 648) | 11.6 (8.7 to 15.4) | -26.2 (-44.8 to -2.7) | 453 (343 to 599) | 10.7 (8.1 to 14.1) | -33.8 (-50.1 to -13.1) | 10156 (7505 to 13546) | 251.6 (186.4 to 335.2) | -35 (-52.3 to -13.9) |
| Algeria | 1554 (1251 to 1917) | 5 (4.1 to 6.1) | -43.1 (-57.4 to -27.6) | 1497 (1216 to 1831) | 5.1 (4.2 to 6.2) | -47 (-59.5 to -32.9) | 35590 (28234 to 44101) | 102.6 (82.5 to 126.7) | -48.1 (-61.8 to -32.6) |
| American Samoa | 7 (6 to 8) | 15.5 (13 to 18.3) | -18 (-33.5 to 0.7) | 7 (6 to 8) | 15.8 (13.4 to 18.6) | -20.3 (-34.6 to -2.8) | 181 (149 to 221) | 368.3 (305.3 to 447.8) | -20.7 (-37 to -0.1) |
| Andorra | 17 (13 to 22) | 12.1 (9.1 to 15.7) | -18.8 (-44.1 to 11.3) | 12 (9 to 15) | 8.3 (6.3 to 10.7) | -33.8 (-54 to -10.3) | 230 (173 to 301) | 165.9 (125.1 to 217) | -34.2 (-55.7 to -7.7) |
| Angola | 930 (725 to 1189) | 8.4 (6.8 to 10.6) | -37.8 (-52.9 to -18.5) | 932 (732 to 1187) | 9 (7.3 to 11.2) | -37.5 (-52.3 to -17.9) | 27564 (21051 to 35860) | 211.9 (166.5 to 269.7) | -41.9 (-56.6 to -21.5) |
| Antigua and Barbuda | 11 (9 to 13) | 11.4 (9.7 to 13.1) | -29.8 (-40.7 to -17.7) | 10 (9 to 12) | 11.1 (9.5 to 12.7) | -32.9 (-43.1 to -21.6) | 232 (196 to 271) | 226.4 (192.5 to 263.6) | -39 (-48.9 to -27.6) |
| Argentina | 5314 (4231 to 6676) | 9.9 (7.9 to 12.4) | -29.9 (-44.2 to -12.3) | 5120 (4755 to 5510) | 9.4 (8.8 to 10.1) | -35.5 (-40.1 to -30.5) | 111640 (104450 to 119103) | 212.7 (199.5 to 227.1) | -35 (-39.6 to -30) |
| Armenia | 552 (461 to 648) | 13.4 (11.2 to 15.7) | -41.4 (-51.1 to -31.3) | 534 (447 to 624) | 13 (10.9 to 15.2) | -42.9 (-52.3 to -33.1) | 12433 (10292 to 14697) | 304.5 (252.9 to 360.3) | -49.8 (-58.3 to -40.1) |
| Australia | 2917 (2282 to 3675) | 7 (5.5 to 8.8) | -30 (-45.4 to -11.8) | 1679 (1503 to 1842) | 3.9 (3.5 to 4.2) | -46.7 (-50.3 to -42.4) | 31687 (29070 to 34126) | 81.4 (75 to 87.7) | -47.2 (-51 to -43.2) |
| Austria | 1524 (1240 to 1848) | 8.4 (6.8 to 10.2) | -59.1 (-67.1 to -50.4) | 1027 (931 to 1118) | 5.4 (5 to 5.9) | -68.6 (-70.7 to -66.4) | 18333 (16939 to 19702) | 110.4 (102.6 to 118.4) | -70.4 (-72.3 to -68.2) |
| Azerbaijan | 1926 (1595 to 2345) | 21.7 (18.1 to 26.4) | -27.1 (-40.2 to -10.2) | 1878 (1556 to 2275) | 22.5 (18.8 to 27.3) | -25.7 (-38.6 to -8.2) | 50182 (41415 to 61450) | 502.5 (418.7 to 608.4) | -37.3 (-48.6 to -21.9) |
| Bahamas | 36 (29 to 44) | 9.3 (7.6 to 11.4) | -33.5 (-46.8 to -17.3) | 34 (28 to 42) | 9.2 (7.5 to 11.1) | -35.8 (-48.2 to -20.8) | 870 (699 to 1079) | 213.4 (172 to 263.3) | -37.5 (-50.8 to -21.4) |
| Bahrain | 49 (39 to 62) | 6.5 (5.2 to 7.8) | -52.7 (-62.3 to -39.5) | 42 (33 to 52) | 6.3 (5.1 to 7.6) | -57.2 (-65.9 to -45.9) | 1149 (900 to 1456) | 114.1 (91.7 to 140) | -61.6 (-69.9 to -50.4) |
| Bangladesh | 8146 (6205 to 10830) | 6.3 (4.8 to 8.3) | -42.7 (-55.9 to -22.6) | 8269 (6313 to 10906) | 6.6 (5.1 to 8.6) | -43 (-56 to -24.1) | 212857 (160909 to 287987) | 155.4 (117.7 to 208.6) | -46.7 (-60.1 to -26.8) |
| Barbados | 53 (44 to 63) | 10.9 (9 to 12.8) | -31.9 (-44.3 to -18.7) | 51 (42 to 60) | 10.4 (8.6 to 12.2) | -36.5 (-47.8 to -24.5) | 1038 (850 to 1242) | 219.7 (180 to 264) | -38.6 (-50.3 to -25.9) |
| Belarus | 2774 (2189 to 3577) | 17.9 (14.1 to 23) | -49.5 (-60.1 to -34.6) | 2225 (1759 to 2823) | 14.1 (11.2 to 17.9) | -57.3 (-66 to -45.4) | 52887 (41324 to 68471) | 349.9 (273.7 to 454.6) | -60.6 (-69.2 to -48.8) |
| Belgium | 1647 (1312 to 2055) | 7.1 (5.6 to 8.8) | -46 (-57.1 to -32.2) | 1278 (1145 to 1395) | 5.1 (4.7 to 5.5) | -57.3 (-60.4 to -54.1) | 21481 (19733 to 23055) | 100.8 (93.2 to 107.6) | -55.7 (-58.9 to -52.4) |
| Belize | 28 (24 to 32) | 10.1 (8.7 to 11.7) | -17 (-31 to -1.7) | 27 (24 to 31) | 10.2 (8.8 to 11.7) | -21.3 (-34 to -7.5) | 720 (620 to 828) | 238.1 (204.9 to 273.9) | -16.1 (-30.2 to -0.3) |
| Benin | 559 (436 to 715) | 12.4 (10 to 15.4) | -22 (-37.8 to -3.5) | 588 (462 to 743) | 13.7 (11.1 to 16.8) | -21.4 (-36.7 to -3.4) | 14658 (11073 to 19342) | 285.5 (221.8 to 367.2) | -25.6 (-42.2 to -5.8) |
| Bermuda | 9 (7 to 10) | 6.7 (5.6 to 8) | -48.9 (-57.4 to -38) | 7 (6 to 8) | 5.1 (4.2 to 6) | -60.4 (-66.9 to -52.3) | 128 (107 to 154) | 103.5 (86.7 to 124.9) | -62.5 (-69.4 to -54.1) |
| Bhutan | 44 (32 to 58) | 8 (5.8 to 10.4) | -14.3 (-37.3 to 20.3) | 45 (33 to 59) | 8.4 (6.2 to 10.9) | -14.2 (-36.4 to 18.4) | 1120 (783 to 1521) | 188.2 (134.1 to 253.3) | -24.3 (-45.7 to 10.1) |
| Bolivia (Plurinational State of) | 2859 (2223 to 3563) | 34 (26.8 to 42) | -24.9 (-40.9 to -3.5) | 2954 (2319 to 3652) | 36.1 (28.8 to 44.3) | -26.4 (-41.7 to -6.3) | 67501 (51158 to 86065) | 749.1 (572.7 to 946.4) | -32.9 (-48.7 to -11.7) |
| Bosnia and Herzegovina | 634 (502 to 801) | 10.8 (8.5 to 13.6) | -20.9 (-37.2 to 0.4) | 607 (483 to 766) | 10.3 (8.2 to 13) | -25.8 (-41 to -5.9) | 13346 (10457 to 17045) | 234.6 (183.9 to 298.8) | -30.1 (-45.5 to -9.8) |
| Botswana | 115 (84 to 150) | 8.7 (6.6 to 11) | -22.2 (-44.1 to 6.1) | 114 (84 to 146) | 9 (7 to 11.4) | -24.1 (-44.9 to 2.1) | 3180 (2248 to 4224) | 207.1 (151.8 to 267.9) | -25.9 (-49 to 3.9) |
| Brazil | 24047 (22646 to 25164) | 10.2 (9.6 to 10.7) | -44 (-46.6 to -41.1) | 22978 (21379 to 24099) | 9.9 (9.1 to 10.4) | -48.3 (-50.8 to -45.5) | 545219 (519168 to 568478) | 226.3 (214.8 to 236.1) | -48.2 (-50.7 to -45.5) |
| Brunei Darussalam | 49 (42 to 56) | 17.3 (15.1 to 19.5) | -51.3 (-59.6 to -42.1) | 36 (31 to 40) | 14.1 (12.4 to 15.9) | -56.9 (-63.9 to -49.3) | 1007 (867 to 1155) | 299.7 (261.9 to 338) | -59.9 (-66.7 to -52.4) |
| Bulgaria | 1634 (1308 to 2037) | 11.6 (9.2 to 14.5) | -40 (-52.6 to -25) | 1549 (1248 to 1923) | 10.8 (8.7 to 13.5) | -44.4 (-55.5 to -30.9) | 33703 (26681 to 42310) | 259.8 (204.4 to 325.4) | -44.1 (-56.2 to -29.2) |
| Burkina Faso | 1186 (961 to 1442) | 14 (11.5 to 16.8) | -16.3 (-32.4 to 2.2) | 1237 (1010 to 1495) | 15.4 (12.7 to 18.3) | -16.1 (-32.1 to 1.7) | 31725 (25258 to 39018) | 328.1 (265.6 to 399.5) | -18.5 (-35.4 to 1.4) |
| Burundi | 395 (302 to 512) | 8.4 (6.6 to 10.7) | -33.7 (-49 to -12.7) | 398 (308 to 513) | 9 (7.1 to 11.4) | -33.2 (-49 to -12) | 11959 (9050 to 15727) | 221 (170 to 284.9) | -36.5 (-52.2 to -14) |
| Cabo Verde | 99 (85 to 113) | 23.8 (20.7 to 27) | -21.2 (-33.1 to -7.4) | 106 (92 to 119) | 25.7 (22.5 to 29) | -21.5 (-32.9 to -7.8) | 2152 (1821 to 2529) | 502.3 (428.4 to 581.9) | -30 (-41.6 to -16.2) |
| Cambodia | 1027 (820 to 1249) | 8.9 (7.1 to 10.6) | -40 (-54 to -22.1) | 1032 (822 to 1243) | 9.3 (7.5 to 11.1) | -40.2 (-54 to -23.2) | 27130 (21399 to 34019) | 213.8 (169.4 to 261.6) | -46 (-59.6 to -28.9) |
| Cameroon | 1428 (1083 to 1872) | 13 (10.1 to 16.6) | -17.3 (-36.6 to 8.1) | 1477 (1130 to 1918) | 14.2 (11.2 to 18) | -17.4 (-36.4 to 6.8) | 38268 (28002 to 51546) | 301.6 (228.4 to 394.7) | -20.9 (-41.2 to 6) |
| Canada | 5428 (4237 to 6802) | 8 (6.2 to 10) | -24 (-41 to -4) | 3135 (2810 to 3403) | 4.4 (4 to 4.8) | -42.1 (-45.7 to -37.9) | 58219 (53837 to 62724) | 90.4 (84.2 to 97.3) | -43.8 (-47.7 to -39.5) |
| Central African Republic | 273 (202 to 360) | 11.8 (8.9 to 15.3) | -27.2 (-44.2 to -6) | 273 (202 to 359) | 12.5 (9.5 to 16) | -27.4 (-43.8 to -7.1) | 8725 (6343 to 11640) | 326.8 (242.9 to 429.2) | -28 (-45.5 to -5.6) |
| Chad | 777 (609 to 980) | 14.9 (11.8 to 18.4) | -1.4 (-21.5 to 22.9) | 816 (641 to 1019) | 16.4 (13 to 20.2) | -0.7 (-20.6 to 22.4) | 20771 (15896 to 26536) | 351.1 (274 to 445.4) | -4.9 (-26.1 to 20.7) |
| Chile | 4769 (3791 to 5963) | 19.9 (15.8 to 24.8) | -40.8 (-53.2 to -25.5) | 4237 (3890 to 4560) | 17.6 (16.2 to 19) | -48.2 (-52 to -44.2) | 85929 (79993 to 91647) | 360 (335.4 to 383.8) | -51 (-54.6 to -47.3) |
| China | 612821 (512997 to 728891) | 30.6 (25.8 to 36.1) | -18.4 (-33.3 to 0.8) | 421539 (353520 to 493176) | 21.7 (18.3 to 25.3) | -42.4 (-52.7 to -30) | 9824993 (8191724 to 11632856) | 481.1 (403.2 to 567.4) | -46.9 (-56.9 to -34.6) |
| Colombia | 7905 (6168 to 10026) | 15 (11.7 to 19) | -49 (-60.7 to -34.8) | 6769 (5285 to 8561) | 12.7 (9.9 to 16.1) | -57.7 (-67 to -46.1) | 154457 (118383 to 196992) | 294.2 (225.4 to 375.6) | -56.1 (-66.7 to -43.5) |
| Comoros | 32 (25 to 41) | 6.7 (5.3 to 8.4) | -28.4 (-46.5 to 22.7) | 34 (26 to 42) | 7.2 (5.8 to 8.9) | -28.6 (-45.7 to 16) | 875 (646 to 1136) | 168.4 (127.2 to 216.3) | -30.3 (-50.1 to 35.6) |
| Congo | 209 (163 to 269) | 8.1 (6.6 to 10) | -44 (-56.5 to -28.1) | 211 (166 to 269) | 8.7 (7.1 to 10.7) | -43.5 (-55.5 to -27.9) | 6103 (4593 to 7977) | 203.1 (159.5 to 260.1) | -48.1 (-61.3 to -31.6) |
| Cook Islands | 2 (1 to 2) | 7.5 (6.2 to 9.1) | -32.4 (-46.4 to -13.6) | 2 (1 to 2) | 6.7 (5.7 to 8.2) | -39.2 (-50.9 to -23) | 36 (29 to 44) | 155.6 (125.2 to 193.8) | -40.6 (-54.8 to -20.8) |
| Costa Rica | 1186 (927 to 1498) | 23.3 (18.2 to 29.4) | -41 (-54.1 to -24.9) | 970 (754 to 1220) | 19 (14.8 to 23.9) | -49.2 (-60.1 to -36.1) | 21288 (16538 to 27201) | 412.3 (319.8 to 525.2) | -49.6 (-61.1 to -35) |
| Croatia | 1077 (860 to 1330) | 12.2 (9.7 to 15.2) | -49.5 (-60 to -36.6) | 845 (677 to 1041) | 9.4 (7.5 to 11.6) | -57.7 (-66.3 to -47) | 16423 (12995 to 20492) | 200.7 (157.8 to 251.7) | -60.1 (-69 to -49.3) |
| Cuba | 1254 (1022 to 1530) | 6.6 (5.4 to 8.1) | -15.8 (-31.3 to 3.8) | 1092 (890 to 1324) | 5.7 (4.6 to 6.9) | -26.5 (-39.8 to -9.8) | 23119 (18549 to 28465) | 125.5 (100.9 to 154.5) | -27 (-41.4 to -9.7) |
| Cyprus | 172 (150 to 197) | 9 (7.8 to 10.3) | -0.1 (-16.4 to 23.6) | 125 (109 to 143) | 6.7 (5.9 to 7.6) | -26.8 (-38.4 to -11.2) | 2450 (2140 to 2808) | 129 (113 to 147.8) | -27 (-38.8 to -10.9) |
| Czechia | 1662 (1360 to 2018) | 8 (6.5 to 9.7) | -56.3 (-64.6 to -46.9) | 1289 (1056 to 1554) | 6.1 (5 to 7.3) | -65.4 (-71.8 to -58.3) | 25683 (20837 to 31217) | 131.6 (106.3 to 160.1) | -65.8 (-72.4 to -58.3) |
| Democratic People's Republic of Korea | 7584 (5987 to 9409) | 23.4 (18.6 to 28.9) | -24.8 (-42.5 to -2) | 7204 (5694 to 8907) | 22.5 (17.9 to 27.6) | -27 (-43.1 to -6.2) | 190397 (147059 to 242071) | 577.2 (449 to 732) | -28.9 (-47 to -4.5) |
| Democratic Republic of the Congo | 2730 (2078 to 3509) | 7.6 (5.9 to 9.6) | -30.6 (-47.6 to -10.4) | 2755 (2105 to 3517) | 8.1 (6.3 to 10.2) | -31.3 (-48.1 to -11.7) | 80182 (59971 to 103652) | 195.5 (149.1 to 250.5) | -31.2 (-49.1 to -10) |
| Denmark | 695 (542 to 878) | 6.2 (4.8 to 7.8) | -44.3 (-56.4 to -29.2) | 507 (460 to 554) | 4.3 (3.9 to 4.7) | -54.2 (-57.9 to -50) | 9609 (8745 to 10458) | 91.2 (83.2 to 99) | -56.8 (-60.6 to -52.9) |
| Djibouti | 45 (33 to 64) | 7.6 (5.8 to 10.1) | -19.1 (-39 to 11.5) | 46 (33 to 63) | 8.2 (6.4 to 10.7) | -18.9 (-37.8 to 10.5) | 1352 (927 to 1961) | 191.1 (140.3 to 265.1) | -21.4 (-43 to 12) |
| Dominica | 18 (15 to 22) | 19.8 (16.2 to 24) | -28.5 (-42.5 to -11.7) | 19 (15 to 22) | 20.5 (16.9 to 24.7) | -28.9 (-42.4 to -12.9) | 388 (314 to 476) | 441.8 (356 to 545) | -31.2 (-45.8 to -13.5) |
| Dominican Republic | 803 (604 to 1061) | 8.6 (6.5 to 11.4) | 20 (-10.2 to 58.8) | 794 (602 to 1039) | 8.7 (6.7 to 11.3) | 13 (-14.5 to 47) | 20051 (14804 to 26924) | 207 (153.3 to 276.8) | 14.1 (-16.8 to 54.3) |
| Ecuador | 3243 (2569 to 4117) | 22.3 (17.8 to 28.2) | -25.7 (-40.5 to -6.8) | 3115 (2497 to 3938) | 21.9 (17.6 to 27.5) | -32.5 (-45.5 to -15.6) | 69342 (54292 to 89319) | 451.5 (354.5 to 580.5) | -35.6 (-49.2 to -18.1) |
| Egypt | 2940 (2195 to 3892) | 5 (3.8 to 6.5) | -11 (-32.2 to 17.6) | 2873 (2166 to 3775) | 5.2 (4 to 6.8) | -14.8 (-35 to 11.8) | 74335 (54850 to 99752) | 111.4 (83.7 to 147.4) | -17.3 (-38.6 to 12) |
| El Salvador | 1044 (802 to 1335) | 17.5 (13.4 to 22.4) | 6.8 (-18.8 to 38.4) | 984 (759 to 1245) | 16.2 (12.5 to 20.6) | -3.8 (-26.8 to 23.3) | 21720 (16424 to 28275) | 368 (277.5 to 479.9) | -9.6 (-32.4 to 19.2) |
| Equatorial Guinea | 28 (20 to 40) | 6.1 (4.5 to 8.4) | -54.9 (-68.5 to -34.1) | 29 (21 to 40) | 6.6 (4.9 to 9) | -54 (-67.3 to -34.1) | 778 (525 to 1158) | 143.8 (101.5 to 203.6) | -61.2 (-74 to -40.9) |
| Eritrea | 280 (207 to 372) | 9.9 (7.6 to 12.7) | -14.7 (-35.9 to 13.7) | 277 (206 to 365) | 10.4 (8.1 to 13.3) | -14.1 (-35.1 to 14.3) | 8784 (6424 to 11963) | 264.4 (197.4 to 346) | -19.6 (-40.2 to 8.5) |
| Estonia | 392 (309 to 497) | 15.8 (12.3 to 20.3) | -45.4 (-57.6 to -30) | 300 (238 to 378) | 11.4 (8.9 to 14.5) | -57.7 (-66.7 to -46.2) | 6180 (4818 to 7904) | 268.4 (208 to 346.1) | -60.6 (-69.6 to -49) |
| Eswatini | 49 (36 to 64) | 8.6 (6.5 to 11.1) | -17.2 (-39.1 to 14.8) | 49 (37 to 65) | 9.2 (7 to 11.8) | -17.6 (-39 to 13.3) | 1397 (998 to 1890) | 217.1 (159.8 to 287.9) | -16.5 (-40.6 to 19) |
| Ethiopia | 2577 (2102 to 3227) | 6.2 (5.1 to 7.7) | -56.2 (-64.8 to -44.4) | 2643 (2157 to 3365) | 6.6 (5.4 to 8.3) | -54.7 (-63.7 to -42.5) | 73965 (59216 to 95576) | 155.4 (126.2 to 196.8) | -61 (-69.2 to -49.1) |
| Fiji | 49 (38 to 62) | 7 (5.6 to 8.8) | -16 (-36.3 to 13) | 48 (38 to 60) | 7.4 (6 to 9.2) | -16.9 (-36.5 to 10.4) | 1322 (1021 to 1693) | 168.1 (132 to 213.8) | -19.6 (-40.2 to 10.2) |
| Finland | 863 (679 to 1078) | 7.2 (5.6 to 9) | -56 (-65.6 to -44.3) | 607 (545 to 665) | 4.8 (4.3 to 5.2) | -64.2 (-67 to -61.1) | 10857 (9941 to 11845) | 100 (92.1 to 109.1) | -65.4 (-68.4 to -62.3) |
| France | 9454 (7387 to 11838) | 6.9 (5.4 to 8.8) | -37.4 (-51.1 to -20.8) | 7238 (6298 to 7953) | 4.9 (4.4 to 5.3) | -51.8 (-55.3 to -48.2) | 123241 (112336 to 133648) | 100.9 (92.7 to 108.8) | -50.9 (-54.4 to -47.1) |
| Gabon | 79 (60 to 98) | 7.7 (6 to 9.4) | -38.4 (-54 to -20.8) | 80 (62 to 100) | 8.2 (6.4 to 10) | -38.3 (-53.5 to -21.4) | 2148 (1611 to 2713) | 188.3 (143.7 to 234.3) | -42.2 (-58.1 to -23.8) |
| Gambia | 47 (37 to 58) | 5.2 (4.1 to 6.4) | -13.4 (-37 to 17.3) | 50 (40 to 62) | 5.8 (4.6 to 7.1) | -13.1 (-35.8 to 15.1) | 1167 (898 to 1488) | 118 (92 to 148.2) | -15 (-40.4 to 19.6) |
| Georgia | 830 (699 to 982) | 14.4 (12.1 to 17) | -27.9 (-40 to -13.6) | 827 (696 to 978) | 14.1 (11.9 to 16.6) | -27.7 (-39.6 to -13.6) | 19437 (16222 to 23091) | 355.5 (298.1 to 423.8) | -32 (-44.1 to -17.6) |
| Germany | 19071 (15003 to 24293) | 10 (7.7 to 12.9) | -39 (-52.7 to -21.4) | 13858 (12524 to 15007) | 6.9 (6.3 to 7.4) | -47.8 (-51.4 to -43.8) | 243443 (224765 to 261548) | 142 (132 to 152.6) | -49.3 (-53 to -45.3) |
| Ghana | 1136 (889 to 1431) | 7.8 (6.2 to 9.7) | -30.1 (-45.5 to -10.2) | 1185 (935 to 1488) | 8.6 (6.9 to 10.8) | -29.6 (-44.6 to -10) | 29454 (22600 to 37798) | 175.6 (137.7 to 221.1) | -34.5 (-49.5 to -14.2) |
| Greece | 2609 (2075 to 3280) | 11 (8.7 to 14) | -35.1 (-49.1 to -17.7) | 2049 (1850 to 2230) | 8 (7.4 to 8.7) | -44.4 (-48.5 to -40.1) | 35610 (32842 to 38259) | 169.8 (157.9 to 181.6) | -44.3 (-48.2 to -39.9) |
| Greenland | 8 (6 to 9) | 11.3 (9.3 to 13.4) | -40.2 (-52.5 to -27) | 7 (6 to 9) | 10.8 (8.8 to 12.9) | -43.1 (-54.7 to -30.5) | 181 (143 to 222) | 251.4 (202.1 to 306) | -46.4 (-58.5 to -32.9) |
| Grenada | 11 (10 to 12) | 10.4 (9.3 to 11.5) | -34.2 (-42.7 to -24.6) | 11 (10 to 12) | 10.4 (9.4 to 11.6) | -36.5 (-44.7 to -27.8) | 256 (227 to 288) | 225.9 (201.1 to 253.2) | -40.5 (-48.6 to -31.8) |
| Guam | 11 (9 to 14) | 6.1 (5.1 to 7.3) | -28.4 (-42.1 to -11.9) | 11 (9 to 13) | 5.7 (4.8 to 6.8) | -35.6 (-47.3 to -21.3) | 268 (221 to 321) | 144.1 (119.4 to 172.1) | -22.3 (-38 to -3.2) |
| Guatemala | 2999 (2369 to 3727) | 27.2 (21.6 to 33.7) | -8.5 (-28.3 to 15.8) | 2959 (2351 to 3670) | 28 (22.5 to 34.4) | -13.4 (-30.7 to 7.7) | 73267 (57182 to 92505) | 615.9 (483.6 to 770.7) | -14.1 (-33.3 to 10) |
| Guinea | 810 (637 to 1010) | 15 (11.9 to 18.6) | -2.7 (-25.4 to 24.1) | 847 (667 to 1049) | 16.2 (12.9 to 20) | -3.1 (-25.2 to 21.8) | 22062 (16954 to 27930) | 370.5 (289.5 to 465.7) | -3.9 (-27.4 to 25.3) |
| Guinea-Bissau | 124 (97 to 155) | 18 (14.3 to 22.2) | -27.1 (-44.9 to -5.6) | 125 (97 to 155) | 19.2 (15.4 to 23.6) | -26.2 (-43.7 to -4.8) | 3500 (2671 to 4451) | 436.3 (339.7 to 544.8) | -31 (-48.7 to -8.3) |
| Guyana | 53 (42 to 67) | 8.6 (6.8 to 10.7) | -39.5 (-52.9 to -22.3) | 53 (41 to 66) | 8.9 (7.1 to 11) | -40.8 (-53.7 to -24.8) | 1435 (1110 to 1829) | 213.4 (166 to 268.8) | -39.5 (-53.9 to -20.8) |
| Haiti | 1032 (656 to 1396) | 15 (9.7 to 20) | -33.4 (-49.9 to -10.7) | 1048 (674 to 1410) | 16 (10.5 to 21.2) | -33.2 (-49.4 to -11) | 29136 (18211 to 39856) | 374.5 (237.7 to 505.8) | -36.3 (-53 to -13.4) |
| Honduras | 918 (736 to 1188) | 15.5 (12.7 to 19.6) | 18.9 (-5.1 to 51.4) | 907 (739 to 1157) | 15.9 (13.2 to 20) | 17.1 (-5.4 to 48.8) | 22310 (17101 to 29701) | 350.3 (276.4 to 455.7) | 2.3 (-20.2 to 33) |
| Hungary | 1836 (1526 to 2191) | 9.6 (7.9 to 11.5) | -51.9 (-60.3 to -42.5) | 1675 (1392 to 1982) | 8.6 (7.1 to 10.2) | -57.2 (-64.8 to -49.1) | 33932 (27964 to 40790) | 189.5 (154.8 to 229.5) | -57.2 (-64.9 to -48.2) |
| Iceland | 40 (35 to 46) | 7.2 (6.3 to 8.3) | -56.3 (-62.8 to -48.9) | 27 (23 to 30) | 4.6 (4 to 5.1) | -64.6 (-68.9 to -60) | 493 (439 to 549) | 94.2 (84.3 to 104.8) | -65.7 (-69.8 to -61) |
| India | 82316 (70723 to 95425) | 7.1 (6.1 to 8.3) | -30.8 (-41.7 to -18.2) | 81771 (70139 to 94685) | 7.3 (6.3 to 8.4) | -32.5 (-43 to -21.1) | 2307059 (1966782 to 2681521) | 187 (159.7 to 217) | -31.9 (-42.7 to -19.9) |
| Indonesia | 12896 (10663 to 14998) | 6.4 (5.4 to 7.3) | -24.8 (-37.4 to -11.6) | 12965 (10817 to 14876) | 6.8 (5.7 to 7.7) | -24.5 (-37.4 to -11) | 333447 (274216 to 391771) | 147.1 (122.2 to 170.2) | -33.2 (-45.4 to -20.3) |
| Iran (Islamic Republic of) | 11736 (10919 to 12644) | 16.8 (15.5 to 18.2) | -29.8 (-38 to -17.6) | 10951 (10144 to 11752) | 16.2 (14.9 to 17.4) | -36 (-43.9 to -24.9) | 254282 (240136 to 272100) | 338.7 (318.5 to 363.1) | -40.1 (-47 to -29.1) |
| Iraq | 1360 (1034 to 1700) | 5.8 (4.5 to 7.1) | -13.9 (-35.9 to 13.8) | 1268 (975 to 1570) | 5.7 (4.6 to 6.9) | -18.3 (-38.8 to 6.4) | 36543 (27357 to 47256) | 137.1 (104.6 to 171.1) | -20.4 (-41.5 to 8.7) |
| Ireland | 656 (513 to 822) | 8.7 (6.8 to 10.9) | -39 (-52.6 to -23.4) | 442 (400 to 483) | 5.8 (5.2 to 6.3) | -55 (-59 to -50.9) | 8262 (7538 to 8971) | 113.3 (103.5 to 122.6) | -57.4 (-61.4 to -53.7) |
| Israel | 890 (699 to 1131) | 7.7 (6 to 9.8) | -30.1 (-45.4 to -10.6) | 716 (646 to 776) | 6 (5.5 to 6.5) | -44.4 (-49 to -39.8) | 13803 (12717 to 14861) | 125.2 (115.8 to 134.7) | -44.1 (-48.9 to -39.6) |
| Italy | 18473 (15261 to 21812) | 12.7 (10.4 to 15.1) | -41.7 (-51.8 to -30.9) | 12861 (11454 to 13683) | 8.3 (7.5 to 8.7) | -55.5 (-57.9 to -53.5) | 217416 (200265 to 228461) | 167.6 (157.4 to 175.2) | -56.7 (-58.7 to -54.9) |
| Jamaica | 291 (231 to 361) | 9.8 (7.7 to 12.1) | -29.9 (-44.7 to -11.7) | 287 (229 to 354) | 9.5 (7.6 to 11.8) | -33.7 (-47.5 to -17.1) | 6306 (4887 to 7977) | 212.5 (164.1 to 269.4) | -31 (-45.8 to -12.5) |
| Japan | 102235 (83884 to 120371) | 28.3 (23.7 to 33.3) | -53.8 (-60.8 to -46.4) | 57162 (48002 to 62076) | 14.1 (12.5 to 15) | -56.3 (-59.5 to -54.2) | 893040 (800034 to 951909) | 282.6 (260.8 to 296.6) | -61.1 (-63.1 to -59.7) |
| Jordan | 334 (277 to 402) | 5.3 (4.5 to 6.4) | -32.5 (-47.8 to -13.7) | 297 (247 to 358) | 5.1 (4.3 to 6.1) | -38.4 (-52.6 to -20.7) | 7979 (6582 to 9646) | 109.5 (91 to 132.1) | -42.4 (-56.7 to -25.1) |
| Kazakhstan | 2554 (2212 to 2929) | 14.6 (12.8 to 16.7) | -56.7 (-62.6 to -50.1) | 2404 (2090 to 2754) | 14.1 (12.3 to 16.1) | -58.4 (-64 to -52.3) | 63596 (54808 to 73430) | 346 (300.3 to 398.3) | -60.9 (-66.5 to -54.8) |
| Kenya | 1878 (1502 to 2271) | 8.6 (7 to 10.3) | -1 (-17 to 17.8) | 2026 (1662 to 2463) | 9.8 (8.1 to 11.8) | 5.5 (-11.2 to 25.3) | 56942 (46243 to 69991) | 227.8 (186.3 to 277.7) | 3.6 (-14.5 to 24.2) |
| Kiribati | 11 (9 to 15) | 15.9 (12.5 to 19.7) | -14.3 (-34.5 to 13.3) | 11 (9 to 14) | 16.6 (13.3 to 20.3) | -14.5 (-34.1 to 12) | 372 (282 to 477) | 441 (341.9 to 554.6) | -17.7 (-38.8 to 10.9) |
| Kuwait | 92 (77 to 111) | 3.9 (3.3 to 4.8) | -37.8 (-48.6 to -24.4) | 74 (62 to 88) | 3.4 (2.9 to 4.1) | -45 (-54.3 to -33.6) | 1805 (1507 to 2181) | 64.9 (54.1 to 77.7) | -50 (-59 to -39.5) |
| Kyrgyzstan | 802 (697 to 911) | 17 (14.8 to 19.2) | -47 (-54.1 to -39.4) | 769 (670 to 873) | 16.8 (14.7 to 19) | -47.2 (-54.2 to -39.8) | 22142 (19190 to 25335) | 433.6 (377 to 491.9) | -51.9 (-58.5 to -44.9) |
| Lao People's Democratic Republic | 332 (250 to 421) | 7.8 (5.9 to 9.7) | -51.3 (-62.8 to -36.5) | 335 (254 to 419) | 8.3 (6.4 to 10.2) | -50.7 (-61.7 to -36.3) | 9265 (6891 to 11899) | 191.1 (144.2 to 242) | -56.4 (-67.7 to -41.5) |
| Latvia | 533 (446 to 642) | 14 (11.8 to 16.9) | -47.4 (-56.4 to -36.5) | 456 (385 to 548) | 11.6 (9.7 to 13.9) | -54 (-61.6 to -44.9) | 9616 (8047 to 11637) | 278.3 (231.4 to 337.9) | -57.4 (-64.9 to -48.7) |
| Lebanon | 423 (341 to 550) | 8.1 (6.5 to 10.6) | -17.8 (-37.8 to 17.9) | 353 (287 to 464) | 6.8 (5.5 to 9) | -33.4 (-49.3 to -3.5) | 7830 (6250 to 10156) | 149.2 (118.9 to 193.6) | -36.1 (-52.7 to -8.9) |
| Lesotho | 144 (108 to 187) | 11.5 (8.7 to 14.7) | 16.1 (-12.9 to 53.9) | 148 (111 to 190) | 12.3 (9.4 to 15.6) | 15.6 (-12.5 to 52.6) | 4167 (3075 to 5469) | 300.6 (224.5 to 391) | 20.4 (-13.1 to 63.4) |
| Liberia | 208 (156 to 274) | 11.1 (8.5 to 14.2) | -25.9 (-43 to -1.1) | 218 (166 to 284) | 12.3 (9.5 to 15.6) | -25.1 (-41.9 to -1.1) | 5542 (3955 to 7402) | 253.9 (190.7 to 333.9) | -29.6 (-47.4 to -3.7) |
| Libya | 288 (220 to 372) | 5.7 (4.4 to 7.2) | -19.7 (-43.1 to 16.3) | 274 (210 to 351) | 5.6 (4.4 to 7.1) | -23.5 (-45.1 to 9.8) | 7230 (5439 to 9491) | 128.5 (98 to 166.4) | -25 (-48.4 to 10.7) |
| Lithuania | 767 (619 to 927) | 13.9 (11.3 to 17) | -47.4 (-57.8 to -35.6) | 664 (539 to 798) | 11.7 (9.5 to 14.1) | -52.5 (-61.9 to -42.2) | 14036 (11256 to 17138) | 281.1 (223.9 to 345.1) | -55.2 (-64.4 to -44.8) |
| Luxembourg | 67 (55 to 81) | 6.7 (5.4 to 8.1) | -51.8 (-60.6 to -40.7) | 50 (43 to 57) | 4.7 (4.1 to 5.4) | -61.9 (-67.3 to -56.2) | 910 (787 to 1035) | 94.1 (81.3 to 107.2) | -64.1 (-69.3 to -58.5) |
| Madagascar | 741 (548 to 967) | 6.6 (5 to 8.5) | -25.4 (-44.1 to -1.5) | 741 (550 to 958) | 7 (5.4 to 8.9) | -25.4 (-43.7 to -2.8) | 22939 (16645 to 30141) | 172.4 (128.3 to 223.7) | -27.7 (-47.2 to -4.5) |
| Malawi | 224 (178 to 272) | 3.3 (2.7 to 3.9) | -31.2 (-44.9 to -14.2) | 233 (187 to 281) | 3.6 (3 to 4.2) | -31.1 (-44.4 to -14.9) | 6076 (4754 to 7616) | 77.3 (61.4 to 94.3) | -32 (-46.8 to -13.4) |
| Malaysia | 1826 (1470 to 2259) | 7.2 (5.9 to 9) | -25.9 (-41 to -8.1) | 1695 (1373 to 2100) | 7 (5.7 to 8.6) | -31.4 (-45.2 to -15.8) | 39785 (31530 to 49536) | 146.2 (117.1 to 181.2) | -37.5 (-50.8 to -21.9) |
| Maldives | 11 (9 to 13) | 3.8 (3.1 to 4.5) | -59.3 (-68.3 to -45.9) | 10 (8 to 11) | 3.6 (3 to 4.3) | -63.8 (-71.5 to -51.9) | 221 (181 to 265) | 68.1 (56 to 81.2) | -70.3 (-77.1 to -60.1) |
| Mali | 1471 (1151 to 1872) | 17.1 (13.7 to 21.5) | -21.6 (-37.6 to 0.1) | 1499 (1184 to 1898) | 18.2 (14.8 to 22.7) | -21.2 (-36.5 to -0.1) | 41744 (31859 to 54052) | 435.2 (339.5 to 555.2) | -25 (-41.9 to -1.5) |
| Malta | 63 (53 to 73) | 6.8 (5.7 to 7.9) | -47.5 (-56.5 to -37) | 47 (42 to 53) | 4.9 (4.3 to 5.5) | -58.7 (-63.8 to -52.9) | 864 (764 to 970) | 100.7 (89.7 to 113.2) | -59.1 (-64.2 to -53.1) |
| Marshall Islands | 6 (4 to 7) | 16.3 (12.5 to 20.5) | -18.4 (-36.8 to 3.1) | 5 (4 to 7) | 17 (13.1 to 21.2) | -19.6 (-37.1 to 1.9) | 180 (135 to 233) | 429.2 (327.4 to 551.9) | -18.6 (-38.3 to 4.8) |
| Mauritania | 195 (148 to 251) | 10.2 (7.9 to 12.9) | -38.7 (-52.5 to -22.1) | 209 (160 to 266) | 11.3 (8.8 to 14.1) | -37.3 (-50.8 to -21.6) | 4643 (3336 to 6172) | 221.8 (165.4 to 289.2) | -45.3 (-59.1 to -28.3) |
| Mauritius | 130 (105 to 158) | 7.6 (6.2 to 9.2) | -48 (-57.7 to -37) | 121 (98 to 146) | 7.1 (5.9 to 8.6) | -52.2 (-60.8 to -42.4) | 2791 (2240 to 3422) | 159 (128.1 to 194) | -53.1 (-62.2 to -42.5) |
| Mexico | 11272 (9786 to 13049) | 9.7 (8.4 to 11.2) | -29.2 (-38.3 to -19) | 10095 (8688 to 11605) | 8.9 (7.6 to 10.2) | -38.7 (-46.3 to -29.4) | 245427 (211082 to 283298) | 203 (174.6 to 234.3) | -33.5 (-42.3 to -22.4) |
| Micronesia (Federated States of) | 12 (8 to 15) | 16.8 (12.6 to 21.1) | -14.9 (-39 to 15.9) | 11 (8 to 14) | 17.4 (13.3 to 21.6) | -16.6 (-39.5 to 12.4) | 349 (231 to 456) | 436.4 (305.2 to 560.4) | -19 (-45.5 to 12.6) |
| Monaco | 9 (7 to 11) | 9.9 (7.8 to 12) | -32 (-47.3 to -11) | 7 (5 to 8) | 6.6 (5.3 to 7.9) | -42.2 (-54.8 to -26) | 115 (92 to 137) | 135.5 (107.8 to 165.4) | -43.9 (-57.4 to -25.5) |
| Mongolia | 984 (755 to 1263) | 43.7 (34.3 to 55.1) | -33.2 (-48.6 to -13.3) | 972 (747 to 1240) | 46 (36.3 to 57.5) | -33.1 (-47.9 to -13.9) | 27681 (20976 to 35801) | 1059.2 (816.8 to 1351.4) | -36.8 (-52.5 to -16.2) |
| Montenegro | 81 (67 to 96) | 8.2 (6.9 to 9.8) | -9.6 (-26.4 to 11.3) | 73 (61 to 86) | 7.5 (6.3 to 8.8) | -14.4 (-30 to 4.8) | 1596 (1324 to 1899) | 165.8 (137.9 to 197.4) | -18.8 (-34.1 to -0.3) |
| Morocco | 1356 (1029 to 1640) | 4.6 (3.5 to 5.5) | -14.9 (-33.8 to 7.2) | 1367 (1046 to 1633) | 4.8 (3.8 to 5.7) | -15.8 (-34.2 to 4.8) | 32870 (24799 to 41001) | 102.7 (78.1 to 125.6) | -20.7 (-40.3 to 2.3) |
| Mozambique | 707 (547 to 897) | 6.9 (5.5 to 8.7) | 2.6 (-21.2 to 31.1) | 736 (574 to 928) | 7.6 (6.1 to 9.4) | 1.5 (-21.4 to 28.6) | 19343 (14542 to 25162) | 166.9 (128.8 to 211.6) | 2.9 (-23 to 36.3) |
| Myanmar | 3089 (2543 to 3801) | 6.9 (5.7 to 8.3) | -51.6 (-63.7 to -37.6) | 3129 (2595 to 3826) | 7.2 (6.1 to 8.7) | -51.6 (-63 to -37.3) | 81351 (65221 to 101746) | 166.7 (135.6 to 206.3) | -57.1 (-68.1 to -42.4) |
| Namibia | 46 (37 to 58) | 3.5 (2.8 to 4.3) | -15.5 (-34.3 to 10.1) | 49 (39 to 60) | 3.8 (3.1 to 4.6) | -16.1 (-34 to 7.7) | 1169 (886 to 1525) | 79.7 (62.4 to 100.8) | -20.6 (-40.4 to 9.4) |
| Nauru | 1 (1 to 1) | 17.9 (14.6 to 21.7) | -12 (-28.9 to 9) | 1 (1 to 1) | 17.9 (14.7 to 21.6) | -14.7 (-30.6 to 5) | 27 (21 to 34) | 451.7 (359.7 to 557.2) | -15.7 (-33.1 to 6.6) |
| Nepal | 1837 (1428 to 2343) | 8.4 (6.6 to 10.7) | -12 (-34.1 to 18.7) | 1881 (1467 to 2384) | 9 (7 to 11.3) | -11.3 (-33.7 to 18.7) | 49001 (37314 to 62733) | 208.6 (160.8 to 267.2) | -20.6 (-42.1 to 9.3) |
| Netherlands | 3521 (2770 to 4370) | 10.2 (8 to 12.7) | -32.3 (-46.6 to -15.2) | 2449 (2194 to 2669) | 6.9 (6.2 to 7.5) | -45 (-49.2 to -40.7) | 42572 (38994 to 45951) | 131.5 (121.2 to 141.8) | -48.9 (-52.6 to -44.9) |
| New Zealand | 532 (438 to 633) | 7 (5.8 to 8.3) | -36.6 (-47.1 to -23.5) | 367 (334 to 394) | 4.6 (4.3 to 5) | -48.5 (-52.1 to -44.5) | 7220 (6709 to 7711) | 101.2 (94.7 to 107.6) | -49.5 (-53.2 to -45.5) |
| Nicaragua | 671 (544 to 805) | 15.8 (13 to 18.7) | 0.1 (-17.7 to 19.9) | 612 (503 to 724) | 15 (12.5 to 17.6) | -8.4 (-24.1 to 8.4) | 14457 (11647 to 17702) | 316.5 (256.8 to 381.7) | -17.8 (-33.6 to 0) |
| Niger | 928 (697 to 1188) | 13.3 (10.1 to 16.7) | -16.9 (-33.4 to 6.9) | 966 (728 to 1235) | 14.7 (11.2 to 18.2) | -15.4 (-31.7 to 8.2) | 24911 (18515 to 32524) | 307.1 (231.9 to 391.5) | -21.8 (-38.7 to 2.6) |
| Nigeria | 2966 (2365 to 3661) | 3.9 (3.2 to 4.8) | -13.9 (-33.5 to 10.9) | 3183 (2530 to 3985) | 4.4 (3.6 to 5.4) | -12.4 (-30.7 to 13.8) | 75113 (57863 to 96063) | 85.7 (67.6 to 108.1) | -19 (-37.8 to 7.7) |
| Niue | 0 (0 to 0) | 11 (9 to 13.3) | -17.5 (-35.7 to 6) | 0 (0 to 0) | 10.4 (8.6 to 12.4) | -24.1 (-40.2 to -3.9) | 5 (4 to 6) | 241.3 (191 to 303.3) | -27.2 (-46 to -2.2) |
| North Macedonia | 518 (409 to 650) | 16.1 (12.9 to 20.2) | -28.3 (-43.6 to -9.9) | 483 (384 to 604) | 15.3 (12.3 to 19.1) | -32.8 (-46.5 to -16.2) | 11180 (8755 to 14076) | 345.8 (271.4 to 434.5) | -37.7 (-51.3 to -21) |
| Northern Mariana Islands | 6 (5 to 7) | 13 (11 to 15.1) | -30.7 (-44.9 to -14.9) | 5 (4 to 6) | 12 (10.3 to 13.9) | -36 (-48.7 to -22.1) | 143 (116 to 171) | 270.5 (224.4 to 322.8) | -37.5 (-52 to -21.6) |
| Norway | 642 (545 to 753) | 6.7 (5.7 to 7.8) | -48.5 (-56.1 to -40.1) | 453 (411 to 489) | 4.5 (4.1 to 4.8) | -58.2 (-60.7 to -55.5) | 8074 (7491 to 8650) | 89 (82.7 to 95.5) | -60.9 (-63.3 to -58.4) |
| Oman | 125 (109 to 145) | 8.6 (7.5 to 9.8) | -33.6 (-48.7 to -9.1) | 103 (90 to 120) | 8 (6.9 to 9.1) | -40.1 (-53.3 to -17.7) | 2836 (2436 to 3330) | 152.3 (132.5 to 175.9) | -49.5 (-61.6 to -30.3) |
| Pakistan | 7057 (5827 to 8596) | 6.5 (5.3 to 7.8) | -1.9 (-21.3 to 27.6) | 7105 (5879 to 8688) | 6.9 (5.7 to 8.3) | -3.6 (-21.9 to 24.4) | 204475 (166415 to 252396) | 162.7 (133.9 to 198.8) | -1.8 (-21.5 to 27.7) |
| Palau | 3 (2 to 3) | 12.2 (9.7 to 15.3) | -12 (-34.6 to 18.3) | 2 (2 to 3) | 11.1 (8.9 to 13.7) | -19.2 (-39.2 to 8.3) | 67 (52 to 88) | 293.3 (227.3 to 376.8) | -17.9 (-41.2 to 14.3) |
| Palestine | 168 (143 to 196) | 7.2 (6.1 to 8.3) | -35.2 (-51.1 to -12.4) | 156 (133 to 181) | 7.1 (6.1 to 8.2) | -37.9 (-53 to -16.7) | 4364 (3693 to 5143) | 160.6 (136.7 to 187.3) | -41 (-56 to -19.1) |
| Panama | 517 (402 to 660) | 12.5 (9.7 to 15.9) | -29.3 (-45.3 to -9.7) | 447 (352 to 567) | 10.8 (8.5 to 13.6) | -37.7 (-51.4 to -21.2) | 10142 (7800 to 13066) | 244 (187.6 to 314.1) | -40.5 (-54.4 to -23.2) |
| Papua New Guinea | 647 (455 to 862) | 13.3 (9.7 to 17.3) | -2.5 (-24.2 to 25.7) | 629 (447 to 833) | 14 (10.3 to 18) | -2.7 (-23.4 to 23.5) | 20091 (14080 to 27070) | 343 (244.6 to 453) | -3.6 (-27.2 to 26.8) |
| Paraguay | 490 (377 to 628) | 8.9 (6.8 to 11.4) | -13.1 (-33.9 to 14.8) | 472 (363 to 602) | 8.7 (6.8 to 11.1) | -19.1 (-37.8 to 5.6) | 11494 (8677 to 14824) | 199.5 (151.3 to 257.4) | -19 (-39.5 to 9.1) |
| Peru | 6272 (4714 to 8204) | 19.6 (14.7 to 25.6) | -22.9 (-43.9 to 4.6) | 5722 (4315 to 7441) | 17.8 (13.4 to 23.1) | -33.6 (-51.2 to -10) | 126185 (93458 to 167788) | 388.1 (288.4 to 515.2) | -36.8 (-55.1 to -12.6) |
| Philippines | 3389 (2781 to 4124) | 4.4 (3.6 to 5.3) | -37.9 (-50.6 to -21.4) | 3315 (2750 to 3949) | 4.5 (3.8 to 5.3) | -39.7 (-51.4 to -26.3) | 91989 (75668 to 110618) | 107.4 (88.8 to 128.5) | -39.8 (-52.1 to -25.6) |
| Poland | 6431 (5454 to 7607) | 9.3 (7.9 to 11.1) | -50.5 (-58.1 to -41.6) | 6776 (5717 to 7952) | 9.7 (8.1 to 11.3) | -52.1 (-59.7 to -43.6) | 143269 (119454 to 169570) | 218.9 (181.9 to 260) | -53.2 (-61.2 to -44.3) |
| Portugal | 3314 (2604 to 4190) | 14.6 (11.4 to 18.6) | -48.1 (-59.4 to -34) | 2950 (2657 to 3198) | 11.9 (10.9 to 12.8) | -57.9 (-60.9 to -54.8) | 54791 (50572 to 58616) | 262.5 (242.9 to 281.1) | -57.9 (-60.8 to -54.7) |
| Puerto Rico | 420 (328 to 533) | 5.8 (4.5 to 7.4) | -50 (-61 to -35.9) | 358 (281 to 448) | 4.7 (3.7 to 6) | -59.1 (-67.9 to -47.7) | 6463 (5021 to 8207) | 99.2 (76.7 to 128.4) | -57.9 (-67.7 to -45.3) |
| Qatar | 56 (42 to 73) | 9 (7 to 11.4) | -24.7 (-45.6 to 2.6) | 41 (31 to 54) | 8.3 (6.5 to 10.4) | -34.7 (-52.5 to -12) | 1264 (942 to 1689) | 137.3 (107 to 177) | -46.8 (-61.7 to -27) |
| Republic of Korea | 25074 (20656 to 29936) | 28.7 (23.7 to 34.2) | -53.4 (-61.4 to -44) | 12250 (10962 to 13503) | 14.1 (12.6 to 15.6) | -73.1 (-75.7 to -70) | 259057 (232823 to 285793) | 298.7 (270.2 to 328.4) | -78 (-80.2 to -75.5) |
| Republic of Moldova | 593 (509 to 680) | 10.4 (9 to 11.9) | -48.6 (-55.9 to -40.7) | 536 (463 to 614) | 9.3 (8 to 10.7) | -52.2 (-58.7 to -45.4) | 13916 (11889 to 16083) | 248.8 (213.7 to 288) | -54.3 (-60.9 to -47) |
| Romania | 4225 (3456 to 5146) | 11.7 (9.5 to 14.4) | -21 (-35.6 to -3.5) | 3840 (3163 to 4639) | 10.5 (8.6 to 12.7) | -29.1 (-42 to -13.5) | 85625 (69923 to 104624) | 254.6 (207.4 to 313.1) | -32.2 (-45.1 to -16.5) |
| Russian Federation | 37113 (32580 to 42443) | 16.1 (14.1 to 18.4) | -48.6 (-54.8 to -41.6) | 30920 (26998 to 35245) | 13.2 (11.5 to 15.1) | -55.2 (-60.4 to -49.1) | 725769 (632299 to 835086) | 320.5 (278.9 to 368.7) | -57.5 (-62.9 to -51.2) |
| Rwanda | 410 (316 to 515) | 6.9 (5.4 to 8.5) | -47.8 (-59.3 to -32.8) | 416 (321 to 521) | 7.4 (5.9 to 9) | -46.9 (-58.2 to -32.3) | 11785 (8848 to 15114) | 172.4 (132.5 to 216.6) | -52.1 (-64 to -37.2) |
| Saint Kitts and Nevis | 7 (6 to 8) | 10.7 (9 to 12.5) | -42.5 (-52.3 to -31) | 6 (5 to 7) | 10 (8.5 to 11.6) | -47.4 (-55.8 to -37.6) | 148 (116 to 178) | 215.2 (172.6 to 256.3) | -50.1 (-60.4 to -38.5) |
| Saint Lucia | 26 (22 to 30) | 12.2 (10.3 to 14.2) | -39.7 (-49.7 to -28.3) | 25 (21 to 30) | 12 (10.2 to 14.1) | -43.4 (-52.2 to -33.3) | 577 (483 to 684) | 266.5 (224.3 to 315.8) | -43.9 (-53.8 to -32.7) |
| Saint Vincent and the Grenadines | 15 (13 to 17) | 11.2 (9.7 to 12.9) | -30.3 (-40.7 to -18.9) | 15 (13 to 17) | 11.4 (10 to 13.1) | -32.3 (-42 to -21.5) | 344 (298 to 399) | 254.8 (221.3 to 294.8) | -32.6 (-43 to -21.2) |
| Samoa | 17 (14 to 22) | 12.2 (9.8 to 15.2) | -20.8 (-39.9 to 2.9) | 17 (14 to 22) | 12.4 (10 to 15.3) | -23 (-40.6 to -1.1) | 449 (344 to 579) | 293.6 (228.5 to 373.8) | -24.3 (-44.6 to 1.4) |
| San Marino | 17 (13 to 22) | 26.2 (20 to 34) | -25.1 (-44.1 to 0.6) | 13 (9 to 18) | 18.6 (12.5 to 25.9) | -35.2 (-57.1 to -6.1) | 218 (145 to 309) | 358.5 (233.4 to 518.5) | -36 (-59.7 to -2.5) |
| Sao Tome and Principe | 16 (13 to 20) | 16.6 (13.5 to 20.7) | 0.5 (-20.4 to 31.5) | 17 (13 to 21) | 18.2 (14.9 to 22.6) | -0.4 (-21 to 29.5) | 393 (308 to 501) | 368.2 (296.3 to 463.4) | -3 (-25.7 to 30.6) |
| Saudi Arabia | 756 (588 to 956) | 4.4 (3.6 to 5.3) | -31.1 (-49.3 to -1.8) | 623 (492 to 778) | 4.1 (3.4 to 5) | -46.1 (-60.6 to -22.1) | 19199 (14693 to 24983) | 86.2 (69.3 to 106.8) | -47 (-62.6 to -21.1) |
| Senegal | 880 (690 to 1087) | 12.7 (10 to 15.5) | -19.9 (-37.8 to 2.7) | 935 (734 to 1149) | 14 (11.2 to 17.1) | -19.2 (-36.4 to 2.8) | 22184 (17067 to 27868) | 288.7 (225.8 to 357.5) | -24.4 (-42.3 to 0) |
| Serbia | 1507 (1195 to 1876) | 9.7 (7.7 to 12.1) | -29 (-45.1 to -9.2) | 1375 (1100 to 1709) | 8.7 (7 to 10.8) | -36.6 (-50.4 to -18.9) | 29823 (23404 to 37571) | 200 (155.7 to 251.6) | -40.3 (-54.4 to -22.8) |
| Seychelles | 8 (7 to 9) | 7.2 (6.2 to 8.2) | -36.4 (-47 to -25.2) | 7 (6 to 8) | 6.9 (6 to 7.8) | -40.6 (-50.1 to -30.5) | 186 (159 to 217) | 161.2 (138.9 to 185.8) | -43.9 (-53.7 to -33.3) |
| Sierra Leone | 419 (320 to 542) | 12.4 (9.7 to 15.8) | -16.5 (-35 to 8) | 441 (337 to 567) | 13.7 (10.7 to 17.2) | -16.4 (-34.5 to 7.7) | 11019 (8231 to 14380) | 289.9 (220.9 to 375.8) | -19 (-38.9 to 7.7) |
| Singapore | 811 (649 to 1010) | 10.7 (8.6 to 13.3) | -60.1 (-68 to -50.1) | 408 (362 to 446) | 5.5 (4.9 to 6.1) | -72.9 (-75.4 to -70.4) | 8245 (7500 to 8935) | 106 (96.2 to 114.9) | -76.1 (-78.2 to -74) |
| Slovakia | 1116 (876 to 1386) | 12.2 (9.6 to 15.2) | -42.3 (-55 to -27.7) | 763 (605 to 946) | 8.3 (6.6 to 10.3) | -53.5 (-63.2 to -42) | 16376 (12684 to 20462) | 183.7 (141.7 to 229.9) | -56.4 (-66.7 to -44.8) |
| Slovenia | 507 (399 to 656) | 11.9 (9.3 to 15.6) | -45.6 (-62 to -24.1) | 384 (302 to 492) | 8.5 (6.7 to 11) | -57.6 (-69.9 to -41.4) | 7186 (5602 to 9374) | 179.3 (137.7 to 234) | -60.4 (-72.4 to -43.9) |
| Solomon Islands | 83 (62 to 104) | 23.7 (18.6 to 28.9) | -5.7 (-28 to 24.4) | 78 (57 to 97) | 23.9 (18.9 to 29) | -6.9 (-28.1 to 22.2) | 2752 (1999 to 3491) | 665 (494.1 to 827.2) | -8.3 (-32 to 22.3) |
| Somalia | 698 (503 to 947) | 9.9 (7.3 to 13.2) | -23.8 (-42.5 to 1.8) | 709 (516 to 947) | 10.6 (7.9 to 14) | -22.9 (-40.8 to 2.1) | 22626 (16192 to 30617) | 273.6 (198.3 to 367.4) | -23.4 (-42.6 to 4) |
| South Africa | 2345 (2176 to 2539) | 5.4 (5 to 5.8) | -33.1 (-39.5 to -26) | 2393 (2233 to 2565) | 5.7 (5.3 to 6.1) | -32.7 (-39.5 to -24.7) | 59895 (55711 to 64638) | 127.3 (118.5 to 137.3) | -39 (-45.3 to -31.5) |
| South Sudan | 254 (181 to 353) | 6.7 (4.9 to 9.1) | -28.8 (-45.8 to -5.1) | 262 (185 to 366) | 7.3 (5.3 to 9.9) | -28.3 (-45.7 to -4.7) | 7373 (5035 to 10758) | 169.5 (118.6 to 239.1) | -30.7 (-50.2 to -3.6) |
| Spain | 10949 (8598 to 13624) | 11.5 (9 to 14.4) | -39.7 (-52.6 to -23.8) | 7241 (6461 to 7873) | 7 (6.4 to 7.6) | -55.1 (-58 to -51.8) | 128298 (118337 to 138581) | 146.8 (136.4 to 158.1) | -56.3 (-59.3 to -53) |
| Sri Lanka | 1343 (1012 to 1778) | 5.4 (4.1 to 7.1) | -39.5 (-55.2 to -19.4) | 1203 (916 to 1585) | 5 (3.9 to 6.5) | -47.7 (-60.7 to -31) | 27514 (20582 to 36567) | 107.4 (81 to 141.5) | -48.3 (-62.5 to -29.5) |
| Sudan | 2756 (1866 to 3668) | 14.9 (10.3 to 19.4) | -10.5 (-31.4 to 16.7) | 2738 (1884 to 3619) | 15.6 (10.9 to 20.2) | -11.9 (-31.5 to 13.5) | 73280 (48466 to 100940) | 354 (238.4 to 476.2) | -17.5 (-38.4 to 10.1) |
| Suriname | 42 (35 to 51) | 7.1 (5.9 to 8.5) | -27.3 (-40.4 to -11) | 42 (35 to 51) | 7.3 (6.1 to 8.7) | -29.8 (-42.1 to -14.8) | 1032 (847 to 1251) | 168.2 (138.8 to 202.6) | -29.9 (-43 to -12.5) |
| Sweden | 1055 (884 to 1227) | 5 (4.2 to 5.9) | -55.3 (-62.3 to -47.8) | 820 (735 to 886) | 3.7 (3.3 to 3.9) | -61.9 (-64.3 to -59.5) | 14113 (13032 to 15063) | 73.5 (68.5 to 78.1) | -63 (-65.3 to -60.7) |
| Switzerland | 1149 (898 to 1439) | 6.6 (5.2 to 8.4) | -53.8 (-64 to -41.2) | 744 (658 to 814) | 4 (3.6 to 4.4) | -62.5 (-65.6 to -59.6) | 13353 (12197 to 14440) | 83.4 (76.3 to 89.9) | -64 (-66.9 to -61) |
| Syrian Arab Republic | 569 (420 to 762) | 5 (3.8 to 6.5) | -13.9 (-39.5 to 25.7) | 534 (397 to 708) | 5 (3.8 to 6.5) | -18.5 (-41.8 to 18.3) | 13664 (9854 to 18680) | 107.2 (79.3 to 143.6) | -25.8 (-49 to 9.5) |
| Taiwan (Province of China) | 6084 (4769 to 7908) | 15.6 (12.2 to 20.3) | -20.5 (-37.3 to 2.3) | 4248 (3335 to 5446) | 10.7 (8.4 to 13.7) | -38.8 (-51.7 to -21.8) | 87391 (67587 to 114667) | 227.6 (177 to 296.6) | -45.1 (-57.4 to -28.8) |
| Tajikistan | 1163 (943 to 1442) | 24 (19.7 to 29.1) | -23.4 (-37.9 to -5.9) | 1143 (928 to 1412) | 25.3 (20.8 to 30.4) | -20.3 (-34.7 to -3.3) | 33875 (27028 to 42229) | 590.2 (479.9 to 724.4) | -31.5 (-45 to -15.3) |
| Thailand | 5925 (4437 to 7816) | 5.9 (4.4 to 7.8) | -45.6 (-59.8 to -26.4) | 5228 (3925 to 6849) | 5.2 (3.9 to 6.9) | -53.1 (-65.1 to -37) | 129784 (95676 to 172065) | 129.5 (96 to 171.3) | -53.4 (-66.2 to -36.6) |
| The Republic of Côte d'Ivoire | 1279 (989 to 1607) | 13.3 (10.8 to 16.4) | -24.3 (-40.6 to -5.1) | 1319 (1033 to 1650) | 14.7 (12 to 17.8) | -23.7 (-39.5 to -5.1) | 35035 (26363 to 45100) | 307.7 (240.5 to 384.5) | -28.3 (-45.9 to -8.1) |
| Timor-Leste | 57 (41 to 71) | 7.3 (5.4 to 9) | -27.2 (-45.4 to -5.8) | 59 (43 to 73) | 7.8 (5.8 to 9.6) | -26.6 (-44.4 to -5.4) | 1457 (1020 to 1860) | 172.8 (123 to 218) | -33.5 (-51.3 to -11) |
| Togo | 435 (340 to 567) | 13 (10.5 to 16.4) | -18 (-34.7 to 5.3) | 446 (351 to 575) | 14.2 (11.5 to 17.7) | -17.5 (-34.1 to 5.3) | 11883 (8978 to 15739) | 304 (237.7 to 392.8) | -20.7 (-38.9 to 4.3) |
| Tokelau | 0 (0 to 0) | 9.7 (7.7 to 12.2) | -26.3 (-44.1 to -3.8) | 0 (0 to 0) | 9.9 (8 to 12.3) | -29.3 (-45.7 to -8.4) | 3 (2 to 4) | 226.7 (172.8 to 290.8) | -31.7 (-50.7 to -7.9) |
| Tonga | 10 (9 to 13) | 13.3 (10.9 to 16.1) | -13.7 (-33.6 to 12.8) | 11 (9 to 13) | 13.9 (11.5 to 16.5) | -15.9 (-34.5 to 9.9) | 257 (204 to 319) | 317.4 (253.5 to 390.8) | -16 (-37 to 12.1) |
| Trinidad and Tobago | 81 (62 to 105) | 4.5 (3.4 to 5.8) | -60.2 (-70.1 to -48.3) | 82 (62 to 105) | 4.5 (3.5 to 5.8) | -62.3 (-71.3 to -51) | 1760 (1318 to 2317) | 95.9 (72.1 to 125.9) | -61.9 (-71.7 to -49.7) |
| Tunisia | 683 (508 to 926) | 5.6 (4.2 to 7.5) | -16.7 (-41.2 to 18.9) | 622 (463 to 833) | 5.2 (3.9 to 6.9) | -26.1 (-47.2 to 3.9) | 14259 (10345 to 19475) | 111.5 (81.7 to 151.3) | -27.6 (-49.7 to 4.9) |
| Turkey | 10487 (8363 to 12871) | 11.9 (9.5 to 14.6) | -40.1 (-53.4 to -22.7) | 9323 (7434 to 11433) | 10.7 (8.6 to 13.2) | -47.3 (-58.9 to -32.4) | 227273 (180063 to 281301) | 250.5 (198.7 to 308.5) | -52.3 (-63.2 to -37.9) |
| Turkmenistan | 375 (298 to 471) | 9.3 (7.4 to 11.7) | -57.7 (-66.5 to -47.3) | 359 (286 to 450) | 9.2 (7.4 to 11.4) | -58.8 (-67.3 to -49) | 10376 (8143 to 13123) | 239 (189.2 to 301.7) | -59.2 (-67.7 to -49) |
| Tuvalu | 1 (1 to 2) | 13.3 (10.3 to 17) | -25 (-43.8 to 0.9) | 1 (1 to 2) | 13.7 (10.7 to 17.4) | -26.3 (-43.9 to -1.6) | 36 (27 to 47) | 337 (254.3 to 443.6) | -28.6 (-47.8 to -2.2) |
| Uganda | 1176 (927 to 1461) | 8.2 (6.6 to 9.8) | -16.3 (-34.3 to 6.5) | 1183 (940 to 1453) | 8.6 (7 to 10.4) | -17.5 (-34.2 to 4.2) | 34612 (26544 to 43979) | 209 (165.3 to 259.1) | -16.1 (-36.4 to 11.1) |
| Ukraine | 11947 (10029 to 14040) | 16.3 (13.7 to 19.2) | -46.2 (-55.1 to -36.7) | 9909 (8406 to 11665) | 13.5 (11.4 to 15.8) | -51.7 (-59.3 to -42.7) | 258830 (217543 to 306929) | 373.7 (314.2 to 444.8) | -51.5 (-59.7 to -41.7) |
| United Arab Emirates | 277 (200 to 374) | 8.8 (6.9 to 11) | -38.2 (-55.1 to -14.4) | 246 (177 to 330) | 9.2 (7.3 to 11.4) | -40.7 (-56.5 to -19.3) | 8339 (5945 to 11343) | 177.8 (134.5 to 225.9) | -42.2 (-59 to -18.2) |
| United Kingdom | 9480 (7941 to 11239) | 7.4 (6.2 to 8.8) | -45.2 (-53.8 to -34.8) | 7548 (6904 to 7926) | 5.6 (5.2 to 5.9) | -52.8 (-54.6 to -50.9) | 127995 (120384 to 133104) | 107.4 (102 to 111.5) | -55.4 (-56.7 to -53.8) |
| United Republic of Tanzania | 1760 (1406 to 2153) | 7.2 (5.8 to 8.6) | -26.4 (-41.8 to -8.7) | 1803 (1456 to 2180) | 7.7 (6.3 to 9.2) | -26.7 (-41.1 to -9.5) | 49708 (38335 to 62481) | 180.4 (143 to 221.1) | -28.2 (-44 to -8.7) |
| United States of America | 32149 (27765 to 37414) | 5.9 (5.1 to 6.9) | -29.1 (-39 to -18) | 19174 (17822 to 20050) | 3.4 (3.2 to 3.5) | -41.3 (-42.9 to -39.4) | 387782 (371464 to 401605) | 75.7 (72.8 to 78.3) | -39.9 (-41.6 to -37.9) |
| United States Virgin Islands | 19 (16 to 22) | 10.3 (8.6 to 12) | -7.1 (-25.5 to 16.1) | 18 (15 to 21) | 9.8 (8.3 to 11.3) | -13.7 (-29.9 to 7.2) | 391 (321 to 461) | 218.8 (177.9 to 262.2) | -14.4 (-32.8 to 11) |
| Uruguay | 610 (482 to 755) | 11.3 (8.9 to 14.2) | -36 (-49.7 to -19.6) | 596 (545 to 646) | 10.7 (9.8 to 11.5) | -40.3 (-45 to -35.4) | 11689 (10814 to 12552) | 234.6 (216.9 to 251.7) | -40.5 (-45.3 to -35.5) |
| Uzbekistan | 2941 (2447 to 3444) | 13.5 (11.4 to 15.6) | -39.7 (-48.9 to -30.1) | 2754 (2287 to 3218) | 13.4 (11.4 to 15.4) | -40 (-49 to -30.8) | 85249 (70603 to 100263) | 338.1 (283.8 to 393.7) | -44.2 (-53.4 to -34.8) |
| Vanuatu | 26 (19 to 34) | 15 (11 to 19.5) | -5.4 (-28.6 to 28) | 26 (19 to 34) | 15.8 (11.7 to 20.3) | -6.7 (-28.5 to 24.1) | 778 (565 to 1034) | 395.9 (289.1 to 520.9) | -4.7 (-30.7 to 31.8) |
| Venezuela (Bolivarian Republic of) | 3998 (3023 to 5142) | 13.9 (10.6 to 17.9) | -28.8 (-45.9 to -8.1) | 3560 (2716 to 4563) | 12.6 (9.6 to 16) | -37.4 (-52.2 to -20.2) | 84802 (63788 to 109996) | 286.1 (215.6 to 370) | -36.3 (-52.4 to -17.1) |
| Viet Nam | 9969 (7901 to 12127) | 10.6 (8.5 to 12.6) | -40.1 (-54.8 to -18.7) | 9060 (7269 to 10917) | 10 (8.1 to 11.8) | -45.8 (-58.4 to -26.9) | 241107 (188254 to 298608) | 240.1 (190.1 to 293.9) | -47.7 (-61.9 to -29) |
| Yemen | 2536 (1926 to 3358) | 19.2 (14.7 to 25.1) | -5.5 (-28.4 to 30.9) | 2541 (1941 to 3345) | 20.2 (15.5 to 26.3) | -5.6 (-27.5 to 28.8) | 70912 (52490 to 96510) | 470.7 (358.2 to 625) | -10.5 (-34.3 to 28.2) |
| Zambia | 568 (434 to 719) | 8.2 (6.5 to 10.1) | -27.6 (-43.8 to -8) | 567 (437 to 716) | 8.7 (6.9 to 10.6) | -28.5 (-44.3 to -8.8) | 17161 (12681 to 22251) | 212.3 (162.3 to 267.4) | -28.8 (-46.4 to -7.2) |
| Zimbabwe | 910 (703 to 1150) | 13.1 (10.2 to 16.4) | 5.3 (-19 to 35.3) | 904 (698 to 1143) | 13.7 (10.8 to 17.2) | 4.6 (-18.8 to 33.5) | 25914 (19516 to 33559) | 326.9 (251.6 to 415.2) | 8.9 (-18.4 to 41.2) |

DALYs=disability adjusted life years. UI=uncertainty interval.

Supplementary Table 5 The prevalence, YLLs and YLDs for stomach cancer for both sex in 2019, and percentage change of age-standardised rates (ASRs) by 204 countries, 1990-2019

|  | YLLs (95% Uncertainty Interval) | | | YLDs (95% Uncertainty Interval) | | |
| --- | --- | --- | --- | --- | --- | --- |
|  | Counts | ASR per 100,000 population (95%UI) | Percentage change in ASRs per 100,000 population(95%UI) | Numbers | ASR per 100,000 population (95%UI) | Percentage change in ASRs per 100,000 population (95%UI) |
| Afghanistan | 114549 (74387 to 156250) | 722.6 (499.8 to 931.3) | -12 (-34.2 to 15.5) | 870 (502 to 1294) | 6.1 (3.8 to 8.8) | -10.2 (-34.9 to 21.2) |
| Albania | 10035 (7408 to 13356) | 248.7 (184.8 to 330.8) | -35.1 (-52.4 to -14.1) | 121 (77 to 179) | 2.9 (1.9 to 4.3) | -21.6 (-44.1 to 8.2) |
| Algeria | 35193 (27928 to 43634) | 101.4 (81.5 to 125.1) | -48.2 (-61.9 to -32.7) | 397 (264 to 561) | 1.2 (0.8 to 1.7) | -39.2 (-55.6 to -18.7) |
| American Samoa | 179 (147 to 219) | 364.7 (302.2 to 442.1) | -20.7 (-36.9 to -0.2) | 2 (1 to 2) | 3.6 (2.5 to 5) | -16.6 (-35.3 to 6.7) |
| Andorra | 225 (170 to 294) | 162.5 (122.4 to 212.1) | -34.6 (-55.9 to -8.4) | 5 (3 to 7) | 3.5 (2.3 to 5.1) | -8.8 (-39.1 to 29.2) |
| Angola | 27332 (20874 to 35522) | 209.9 (164.9 to 267.9) | -41.9 (-56.6 to -21.7) | 232 (153 to 338) | 2 (1.3 to 2.8) | -37.1 (-55.8 to -12.3) |
| Antigua and Barbuda | 229 (194 to 268) | 223.7 (190.1 to 260.3) | -39.1 (-49 to -27.7) | 3 (2 to 4) | 2.7 (1.8 to 3.7) | -28.1 (-41.9 to -11.1) |
| Argentina | 110341 (103266 to 117654) | 210.3 (197.3 to 223.8) | -35.1 (-39.7 to -30.1) | 1299 (851 to 1880) | 2.4 (1.6 to 3.5) | -26.1 (-44.3 to -4) |
| Armenia | 12300 (10185 to 14555) | 301.3 (250.6 to 357.4) | -49.9 (-58.4 to -40.3) | 133 (91 to 180) | 3.2 (2.2 to 4.4) | -40 (-53.6 to -23.1) |
| Australia | 30800 (28376 to 33243) | 79.2 (73.2 to 85.1) | -47.7 (-51.4 to -43.9) | 887 (587 to 1269) | 2.2 (1.4 to 3.1) | -19.9 (-40.9 to 5.8) |
| Austria | 17903 (16580 to 19232) | 108 (100.5 to 115.5) | -70.6 (-72.5 to -68.6) | 430 (284 to 600) | 2.5 (1.6 to 3.5) | -52 (-62.6 to -39.6) |
| Azerbaijan | 49720 (41057 to 60818) | 497.5 (414.7 to 603.1) | -37.4 (-48.6 to -22.1) | 463 (304 to 649) | 5 (3.4 to 6.9) | -28.6 (-43.4 to -8) |
| Bahamas | 861 (691 to 1070) | 211.1 (170.3 to 260.5) | -37.5 (-50.8 to -21.5) | 9 (6 to 12) | 2.3 (1.5 to 3.2) | -31.5 (-48.1 to -11.3) |
| Bahrain | 1136 (890 to 1437) | 112.6 (90.4 to 138.2) | -61.8 (-70.1 to -50.5) | 13 (9 to 19) | 1.5 (1 to 2.1) | -48.7 (-61.6 to -30) |
| Bangladesh | 210836 (159395 to 285210) | 153.9 (116.7 to 206.5) | -46.8 (-60.1 to -26.8) | 2021 (1310 to 2958) | 1.5 (1 to 2.2) | -40.8 (-56.7 to -17) |
| Barbados | 1025 (839 to 1227) | 217 (178.1 to 261) | -38.7 (-50.4 to -25.9) | 13 (9 to 18) | 2.7 (1.8 to 3.7) | -29.3 (-45 to -9.9) |
| Belarus | 52171 (40765 to 67610) | 345.2 (270.4 to 448.4) | -60.8 (-69.3 to -49) | 717 (471 to 1028) | 4.7 (3.1 to 6.7) | -44.4 (-59.2 to -25.6) |
| Belgium | 21040 (19331 to 22585) | 98.8 (91.5 to 105.5) | -56 (-59.1 to -52.8) | 441 (288 to 629) | 2 (1.3 to 2.8) | -37.3 (-52.4 to -18.5) |
| Belize | 713 (614 to 820) | 235.6 (202.8 to 270.9) | -16.2 (-30.4 to -0.3) | 7 (5 to 10) | 2.5 (1.7 to 3.4) | -13 (-32.6 to 10.3) |
| Benin | 14526 (10970 to 19157) | 282.7 (220 to 363.1) | -25.6 (-42.2 to -5.8) | 131 (85 to 183) | 2.8 (1.8 to 3.8) | -21.8 (-40.8 to 2.7) |
| Bermuda | 126 (105 to 151) | 101.6 (84.8 to 122.6) | -62.7 (-69.5 to -54.4) | 2 (2 to 3) | 1.8 (1.2 to 2.6) | -41.3 (-53.9 to -24.3) |
| Bhutan | 1110 (776 to 1507) | 186.3 (132.7 to 250.9) | -24.4 (-45.8 to 10.1) | 11 (7 to 16) | 1.9 (1.2 to 2.8) | -14.2 (-39.4 to 28.8) |
| Bolivia (Plurinational State of) | 66855 (50733 to 85280) | 741.6 (567.3 to 935.4) | -33 (-48.8 to -11.7) | 646 (424 to 923) | 7.5 (5 to 10.7) | -24.5 (-42.9 to 1.7) |
| Bosnia and Herzegovina | 13194 (10351 to 16848) | 232 (181.6 to 295.4) | -30.2 (-45.5 to -9.9) | 152 (100 to 219) | 2.6 (1.7 to 3.8) | -18.7 (-39.2 to 8.3) |
| Botswana | 3151 (2232 to 4186) | 205 (150.3 to 264.8) | -25.9 (-49 to 3.9) | 29 (18 to 43) | 2 (1.3 to 3) | -20.5 (-45.7 to 14.2) |
| Brazil | 539304 (513840 to 562619) | 223.8 (212.4 to 233.6) | -48.3 (-50.8 to -45.5) | 5915 (4252 to 7603) | 2.5 (1.8 to 3.2) | -39.8 (-44 to -35.3) |
| Brunei Darussalam | 993 (857 to 1141) | 295.3 (258 to 332.6) | -60.1 (-66.8 to -52.5) | 14 (9 to 18) | 4.4 (3.1 to 5.9) | -47 (-58.3 to -34.5) |
| Bulgaria | 33309 (26426 to 41792) | 256.9 (202.1 to 321.9) | -44.2 (-56.3 to -29.3) | 394 (261 to 563) | 2.9 (1.9 to 4.1) | -36.2 (-52.7 to -16.4) |
| Burkina Faso | 31444 (25051 to 38736) | 325 (263 to 395.2) | -18.6 (-35.5 to 1.3) | 281 (183 to 393) | 3.2 (2.1 to 4.4) | -14.7 (-35.7 to 16.7) |
| Burundi | 11861 (8962 to 15604) | 219 (168.6 to 283.7) | -36.6 (-52.2 to -14.1) | 99 (63 to 144) | 2 (1.3 to 2.9) | -32.7 (-50.3 to -6.6) |
| Cabo Verde | 2130 (1796 to 2502) | 497 (423.6 to 576.8) | -30.1 (-41.6 to -16.2) | 22 (15 to 30) | 5.3 (3.7 to 7.2) | -21.6 (-37.3 to -3) |
| Cambodia | 26879 (21179 to 33689) | 211.7 (168 to 259.4) | -46.1 (-59.6 to -28.9) | 251 (171 to 351) | 2.1 (1.4 to 2.9) | -39.2 (-55.2 to -17.9) |
| Cameroon | 37929 (27748 to 51046) | 298.6 (226.7 to 390.7) | -21 (-41.3 to 6.1) | 339 (211 to 496) | 2.9 (1.9 to 4.2) | -16.5 (-39.7 to 14.7) |
| Canada | 56558 (52319 to 60726) | 87.9 (81.9 to 94.5) | -44.4 (-48.2 to -40.2) | 1660 (1081 to 2389) | 2.5 (1.6 to 3.6) | -13.3 (-34.7 to 15.5) |
| Central African Republic | 8657 (6293 to 11547) | 324 (241.2 to 425.1) | -28.1 (-45.6 to -5.8) | 67 (42 to 100) | 2.8 (1.7 to 4) | -26 (-47.1 to -0.2) |
| Chad | 20590 (15716 to 26267) | 347.8 (271.8 to 441.4) | -5 (-26 to 20.6) | 182 (118 to 258) | 3.3 (2.2 to 4.7) | -2.4 (-26.1 to 27.8) |
| Chile | 84760 (78941 to 90242) | 355.2 (330.5 to 378.2) | -51.2 (-54.7 to -47.5) | 1169 (762 to 1660) | 4.9 (3.2 to 6.9) | -36 (-51.4 to -17.9) |
| China | 9651544 (8011210 to 11463275) | 472.6 (394.6 to 558.6) | -47.3 (-57.3 to -34.9) | 173449 (121686 to 234275) | 8.6 (6 to 11.5) | -1.7 (-20.7 to 23.6) |
| Colombia | 152431 (117038 to 194320) | 290.4 (223 to 370.5) | -56.3 (-66.8 to -43.8) | 2026 (1303 to 2911) | 3.9 (2.5 to 5.5) | -42.2 (-56.6 to -22) |
| Comoros | 867 (640 to 1124) | 166.8 (125.4 to 214.4) | -30.3 (-50.1 to 35.5) | 8 (5 to 11) | 1.6 (1.1 to 2.3) | -27.1 (-48.7 to 32.7) |
| Congo | 6051 (4555 to 7903) | 201.2 (157.7 to 257.7) | -48.2 (-61.4 to -31.6) | 52 (33 to 76) | 1.9 (1.2 to 2.8) | -42.9 (-58.6 to -21.8) |
| Cook Islands | 36 (29 to 44) | 153.7 (123.4 to 191.5) | -40.7 (-55 to -20.9) | 0 (0 to 1) | 1.9 (1.3 to 2.7) | -27.7 (-45.6 to -2.3) |
| Costa Rica | 20984 (16300 to 26786) | 406.3 (315.9 to 517.9) | -49.7 (-61.2 to -35.2) | 304 (199 to 434) | 5.9 (3.9 to 8.5) | -35.9 (-51.9 to -15.6) |
| Croatia | 16146 (12769 to 20156) | 197.5 (155.4 to 247.4) | -60.3 (-69.2 to -49.5) | 277 (185 to 388) | 3.2 (2.1 to 4.6) | -43.8 (-58.1 to -26) |
| Cuba | 22795 (18302 to 28048) | 123.8 (99.3 to 152.4) | -27.1 (-41.6 to -9.9) | 324 (212 to 455) | 1.7 (1.1 to 2.4) | -11 (-31.4 to 13.4) |
| Cyprus | 2402 (2098 to 2756) | 126.6 (110.9 to 144.9) | -27.5 (-39.2 to -11.7) | 48 (33 to 65) | 2.5 (1.8 to 3.4) | 17.5 (-7.6 to 51.2) |
| Czechia | 25245 (20466 to 30675) | 129.4 (104.3 to 157.7) | -66 (-72.6 to -58.4) | 438 (293 to 606) | 2.2 (1.5 to 3) | -49.3 (-60.7 to -35.9) |
| Democratic People's Republic of Korea | 188540 (145828 to 239862) | 571.5 (444.9 to 724.4) | -29 (-47 to -4.5) | 1857 (1205 to 2656) | 5.7 (3.7 to 8.1) | -23 (-44.4 to 4.6) |
| Democratic Republic of the Congo | 79506 (59509 to 102776) | 193.7 (147.9 to 248.1) | -31.2 (-49.2 to -10) | 676 (430 to 969) | 1.8 (1.2 to 2.6) | -29 (-49.3 to -3.7) |
| Denmark | 9414 (8572 to 10231) | 89.4 (81.5 to 96.9) | -57.1 (-60.9 to -53.3) | 196 (129 to 282) | 1.8 (1.2 to 2.6) | -37.6 (-52.5 to -16.5) |
| Djibouti | 1341 (918 to 1947) | 189.3 (139 to 262.7) | -21.4 (-43 to 12) | 11 (7 to 17) | 1.8 (1.1 to 2.7) | -19 (-42.1 to 15.9) |
| Dominica | 384 (311 to 471) | 437.3 (352.1 to 539.1) | -31.2 (-45.8 to -13.4) | 4 (3 to 6) | 4.5 (3 to 6.3) | -28.6 (-45.8 to -6.9) |
| Dominican Republic | 19851 (14660 to 26618) | 204.8 (151.9 to 273.7) | 14 (-16.8 to 54.2) | 200 (126 to 301) | 2.1 (1.3 to 3.2) | 21.4 (-13.4 to 65.9) |
| Ecuador | 68574 (53697 to 88293) | 446.3 (350.4 to 573.6) | -35.8 (-49.3 to -18.2) | 768 (503 to 1077) | 5.2 (3.4 to 7.3) | -22.7 (-40.8 to 0.3) |
| Egypt | 73590 (54264 to 98559) | 110.2 (82.9 to 145.9) | -17.3 (-38.7 to 11.9) | 745 (461 to 1098) | 1.2 (0.8 to 1.8) | -10 (-34.9 to 20.7) |
| El Salvador | 21468 (16236 to 27986) | 363.8 (273.9 to 474.2) | -9.8 (-32.5 to 19) | 251 (163 to 366) | 4.2 (2.7 to 6.2) | 10.6 (-19.6 to 47.9) |
| Equatorial Guinea | 771 (520 to 1148) | 142.3 (100.5 to 201.8) | -61.3 (-74.1 to -41) | 7 (4 to 11) | 1.4 (0.9 to 2.2) | -53.9 (-68.8 to -30.8) |
| Eritrea | 8715 (6366 to 11864) | 262 (195.6 to 343.3) | -19.6 (-40.2 to 8.5) | 70 (43 to 103) | 2.3 (1.5 to 3.5) | -14.5 (-39.4 to 18.3) |
| Estonia | 6076 (4734 to 7745) | 264 (204.9 to 341.3) | -60.8 (-69.8 to -49.3) | 104 (68 to 147) | 4.4 (2.9 to 6.3) | -36.5 (-52.8 to -15.1) |
| Eswatini | 1386 (991 to 1873) | 215.1 (158.2 to 285.2) | -16.5 (-40.6 to 18.9) | 12 (7 to 18) | 2 (1.3 to 2.9) | -16.6 (-41.8 to 20.7) |
| Ethiopia | 73324 (58597 to 94764) | 153.9 (125 to 194.6) | -61 (-69.3 to -49.1) | 641 (434 to 897) | 1.5 (1 to 2.1) | -55.2 (-64.8 to -42.5) |
| Fiji | 1310 (1011 to 1678) | 166.5 (130.8 to 211.4) | -19.6 (-40.2 to 10.2) | 12 (8 to 17) | 1.7 (1.1 to 2.4) | -16.1 (-40 to 14.8) |
| Finland | 10617 (9752 to 11572) | 97.9 (90.4 to 106.8) | -65.7 (-68.6 to -62.6) | 241 (160 to 343) | 2.1 (1.4 to 3) | -49.5 (-61.8 to -33.8) |
| France | 120650 (110057 to 130114) | 98.9 (91 to 106.5) | -51.2 (-54.7 to -47.4) | 2590 (1711 to 3668) | 2 (1.3 to 2.9) | -26.9 (-46.2 to -1.5) |
| Gabon | 2129 (1596 to 2692) | 186.5 (142.1 to 231.8) | -42.2 (-58.2 to -23.9) | 19 (12 to 28) | 1.8 (1.1 to 2.6) | -37.4 (-55.6 to -15.3) |
| Gambia | 1156 (889 to 1474) | 116.8 (90.9 to 146.4) | -15.1 (-40.4 to 19.6) | 11 (7 to 16) | 1.2 (0.8 to 1.7) | -12.7 (-39.2 to 23.6) |
| Georgia | 19241 (16079 to 22819) | 352.1 (295.2 to 420.1) | -32 (-44.1 to -17.6) | 196 (135 to 267) | 3.4 (2.4 to 4.7) | -28.6 (-43.9 to -10.7) |
| Germany | 238267 (220416 to 255797) | 139.2 (129.4 to 149.7) | -49.6 (-53.2 to -45.7) | 5176 (3430 to 7259) | 2.8 (1.9 to 4) | -32.6 (-50.2 to -11.1) |
| Ghana | 29180 (22392 to 37473) | 173.8 (136 to 218.5) | -34.5 (-49.5 to -14.3) | 274 (175 to 397) | 1.8 (1.2 to 2.6) | -30.2 (-47.9 to -5.5) |
| Greece | 34926 (32271 to 37492) | 166.8 (155.2 to 178.3) | -44.5 (-48.3 to -40.2) | 684 (447 to 982) | 3.1 (2 to 4.4) | -28.7 (-46.6 to -6.3) |
| Greenland | 179 (142 to 220) | 248.7 (199.5 to 302.9) | -46.5 (-58.6 to -32.9) | 2 (1 to 3) | 2.7 (1.8 to 3.7) | -37.4 (-53.4 to -18.7) |
| Grenada | 253 (225 to 284) | 223.5 (199.3 to 250.2) | -40.6 (-48.7 to -31.9) | 3 (2 to 4) | 2.5 (1.7 to 3.3) | -32.3 (-45.3 to -16.4) |
| Guam | 265 (219 to 318) | 142.5 (118.2 to 170.3) | -22.3 (-37.9 to -3.2) | 3 (2 to 4) | 1.6 (1 to 2.1) | -22.7 (-40.5 to -1.8) |
| Guatemala | 72562 (56590 to 91512) | 609.7 (477.5 to 764.7) | -14.1 (-33.3 to 10) | 705 (464 to 1004) | 6.2 (4 to 8.8) | -5.7 (-28.9 to 24.4) |
| Guinea | 21871 (16818 to 27689) | 367.1 (287 to 461.2) | -3.9 (-27.4 to 25.2) | 191 (122 to 276) | 3.4 (2.2 to 4.9) | -2.8 (-29.8 to 31) |
| Guinea-Bissau | 3471 (2646 to 4407) | 432.3 (336.4 to 539.6) | -31 (-48.7 to -8.3) | 29 (19 to 42) | 4.1 (2.7 to 5.7) | -27.3 (-47.3 to -1.6) |
| Guyana | 1422 (1100 to 1814) | 211.3 (164.3 to 265.9) | -39.5 (-54 to -20.8) | 13 (8 to 19) | 2.1 (1.3 to 2.9) | -36.7 (-53.6 to -14.5) |
| Haiti | 28890 (18000 to 39601) | 371 (236.3 to 502.6) | -36.3 (-53 to -13.4) | 246 (144 to 365) | 3.4 (2 to 5.1) | -32.6 (-51 to -6.3) |
| Honduras | 22090 (16943 to 29438) | 346.7 (273.3 to 451.1) | 2.2 (-20.4 to 33) | 220 (143 to 320) | 3.6 (2.4 to 5.2) | 16.3 (-12 to 53.9) |
| Hungary | 33481 (27506 to 40309) | 187.1 (153 to 226.9) | -57.3 (-65 to -48.3) | 451 (307 to 619) | 2.4 (1.6 to 3.3) | -46.7 (-58.3 to -33.5) |
| Iceland | 481 (428 to 536) | 92 (82.6 to 102.4) | -65.9 (-70 to -61.3) | 11 (8 to 15) | 2.1 (1.5 to 2.9) | -50.8 (-60.7 to -39.4) |
| India | 2286769 (1943748 to 2660232) | 185.2 (157.8 to 215.5) | -32 (-42.8 to -19.9) | 20290 (13992 to 27412) | 1.7 (1.2 to 2.3) | -28.1 (-40 to -14.8) |
| Indonesia | 330254 (271589 to 387883) | 145.6 (120.8 to 168.2) | -33.3 (-45.5 to -20.3) | 3194 (2178 to 4294) | 1.5 (1 to 2) | -24.6 (-38.3 to -9.6) |
| Iran (Islamic Republic of) | 251428 (237305 to 269306) | 334.7 (314.6 to 358.5) | -40.2 (-47.1 to -29.2) | 2854 (2054 to 3675) | 4 (2.8 to 5.1) | -26.2 (-35.3 to -12.2) |
| Iraq | 36185 (27115 to 46787) | 135.7 (103.3 to 169.2) | -20.5 (-41.5 to 8.5) | 358 (225 to 519) | 1.5 (0.9 to 2.1) | -10.2 (-34.9 to 24.3) |
| Ireland | 8075 (7379 to 8756) | 110.8 (101.4 to 119.8) | -57.8 (-61.7 to -54.1) | 187 (123 to 268) | 2.5 (1.7 to 3.6) | -28.1 (-46.2 to -3.7) |
| Israel | 13564 (12525 to 14538) | 123.1 (114.1 to 131.8) | -44.4 (-49 to -40) | 239 (156 to 345) | 2.1 (1.4 to 3.1) | -21 (-41.5 to 4.9) |
| Italy | 212369 (196022 to 223067) | 163.9 (153.8 to 171) | -57 (-59 to -55.3) | 5046 (3536 to 6781) | 3.7 (2.5 to 5) | -32 (-44 to -18.3) |
| Jamaica | 6235 (4832 to 7885) | 210.1 (162.2 to 266) | -31 (-45.8 to -12.7) | 71 (47 to 102) | 2.4 (1.6 to 3.4) | -26.9 (-45.4 to -2.7) |
| Japan | 862417 (771689 to 916779) | 273.5 (252.9 to 286.6) | -61.3 (-63.3 to -59.9) | 30623 (21439 to 40464) | 9.1 (6.4 to 12.1) | -52.5 (-59.5 to -44.4) |
| Jordan | 7890 (6515 to 9541) | 108.2 (90 to 130.5) | -42.6 (-56.7 to -25.3) | 89 (59 to 125) | 1.4 (0.9 to 1.9) | -28.5 (-46.8 to -3.3) |
| Kazakhstan | 62971 (54241 to 72625) | 342.5 (297.1 to 394.2) | -61 (-66.6 to -54.8) | 625 (434 to 846) | 3.5 (2.5 to 4.8) | -54.9 (-63.8 to -44.5) |
| Kenya | 56481 (45803 to 69438) | 225.8 (184.7 to 275.1) | 3.7 (-14.5 to 24.4) | 461 (310 to 639) | 2 (1.4 to 2.8) | -0.6 (-17.5 to 19.4) |
| Kiribati | 369 (279 to 473) | 437.2 (338.6 to 549.9) | -17.7 (-38.9 to 10.8) | 3 (2 to 4) | 3.7 (2.5 to 5.1) | -13.5 (-38.1 to 18.9) |
| Kuwait | 1780 (1486 to 2149) | 63.8 (53.2 to 76.3) | -50.2 (-59.2 to -39.7) | 26 (18 to 35) | 1 (0.7 to 1.4) | -33.4 (-48.4 to -13.2) |
| Kyrgyzstan | 21943 (19069 to 25100) | 429.5 (373.8 to 486.7) | -51.9 (-58.5 to -45) | 199 (138 to 271) | 4.1 (2.8 to 5.6) | -46 (-56 to -34.4) |
| Lao People's Democratic Republic | 9183 (6842 to 11784) | 189.3 (142.8 to 239.5) | -56.4 (-67.8 to -41.6) | 83 (53 to 119) | 1.9 (1.2 to 2.6) | -50 (-63 to -31.2) |
| Latvia | 9482 (7936 to 11462) | 274.6 (228.5 to 334.2) | -57.5 (-65.1 to -48.9) | 134 (91 to 184) | 3.7 (2.5 to 5) | -42.3 (-54.6 to -27.3) |
| Lebanon | 7719 (6167 to 10025) | 147.1 (117.3 to 191.2) | -36.4 (-52.9 to -9.2) | 111 (73 to 158) | 2.1 (1.4 to 3) | -9 (-34.4 to 32) |
| Lesotho | 4132 (3048 to 5420) | 298 (222.5 to 386.9) | 20.4 (-13 to 63.4) | 35 (22 to 52) | 2.7 (1.7 to 3.9) | 15.8 (-18.1 to 59.2) |
| Liberia | 5493 (3919 to 7323) | 251.4 (188.8 to 330) | -29.7 (-47.4 to -3.8) | 49 (30 to 72) | 2.5 (1.6 to 3.6) | -25.4 (-46.2 to 5) |
| Libya | 7156 (5373 to 9408) | 127.1 (96.7 to 164.5) | -25.1 (-48.4 to 10.6) | 74 (47 to 107) | 1.4 (0.9 to 2) | -17.7 (-43.5 to 23.3) |
| Lithuania | 13846 (11098 to 16892) | 277.5 (220.8 to 340) | -55.3 (-64.5 to -45.1) | 190 (131 to 264) | 3.6 (2.4 to 5) | -43.5 (-56.5 to -26.8) |
| Luxembourg | 892 (770 to 1010) | 92.1 (79.8 to 104.8) | -64.4 (-69.5 to -58.9) | 19 (13 to 26) | 1.9 (1.3 to 2.7) | -44 (-56.7 to -27.8) |
| Madagascar | 22751 (16515 to 29858) | 170.9 (127 to 221.7) | -27.8 (-47.2 to -4.5) | 188 (119 to 276) | 1.6 (1 to 2.3) | -24.5 (-45.1 to 4.9) |
| Malawi | 6021 (4711 to 7533) | 76.6 (60.9 to 93.2) | -32 (-46.8 to -13.5) | 56 (38 to 80) | 0.8 (0.5 to 1.1) | -29.8 (-45.5 to -9) |
| Malaysia | 39324 (31154 to 48910) | 144.4 (115.7 to 179.3) | -37.7 (-50.9 to -22.1) | 461 (302 to 655) | 1.8 (1.2 to 2.5) | -23 (-42.9 to 1.2) |
| Maldives | 218 (179 to 261) | 67.1 (55.1 to 80) | -70.4 (-77.2 to -60.3) | 3 (2 to 4) | 1 (0.7 to 1.3) | -56 (-67.5 to -38.2) |
| Mali | 41391 (31583 to 53496) | 431.2 (336.6 to 550.4) | -25 (-41.9 to -1.6) | 353 (236 to 514) | 4 (2.7 to 5.6) | -20.7 (-39.3 to 5.2) |
| Malta | 847 (748 to 948) | 98.8 (87.9 to 110.7) | -59.4 (-64.4 to -53.5) | 17 (12 to 23) | 1.9 (1.3 to 2.6) | -39.9 (-52.8 to -22.8) |
| Marshall Islands | 179 (134 to 231) | 425.4 (324.6 to 547.1) | -18.7 (-38.3 to 4.7) | 1 (1 to 2) | 3.8 (2.4 to 5.4) | -17 (-37.1 to 11.8) |
| Mauritania | 4598 (3303 to 6129) | 219.5 (163.9 to 286.9) | -45.4 (-59.2 to -28.3) | 46 (29 to 65) | 2.3 (1.5 to 3.2) | -38.6 (-54.5 to -18) |
| Mauritius | 2758 (2217 to 3380) | 157.1 (126.7 to 191.8) | -53.2 (-62.3 to -42.6) | 32 (22 to 45) | 1.9 (1.2 to 2.6) | -44.5 (-58.2 to -27.5) |
| Mexico | 242547 (208229 to 280102) | 200.5 (172.3 to 231.7) | -33.6 (-42.5 to -22.4) | 2880 (2014 to 3836) | 2.4 (1.7 to 3.2) | -21.6 (-32.5 to -9.6) |
| Micronesia (Federated States of) | 346 (229 to 452) | 432.4 (302 to 555.4) | -19 (-45.6 to 12.6) | 3 (2 to 4) | 4 (2.4 to 5.7) | -13.8 (-40.6 to 21.5) |
| Monaco | 112 (90 to 134) | 132.6 (105.4 to 161.7) | -44.2 (-57.6 to -25.7) | 3 (2 to 4) | 2.9 (1.9 to 4) | -25.8 (-45.6 to -0.6) |
| Mongolia | 27448 (20794 to 35556) | 1049.4 (808.7 to 1337.1) | -36.8 (-52.5 to -16.3) | 233 (153 to 339) | 9.9 (6.5 to 14) | -32.6 (-50 to -9.6) |
| Montenegro | 1576 (1308 to 1877) | 163.7 (136.2 to 194.9) | -18.9 (-34.1 to -0.3) | 20 (14 to 28) | 2.1 (1.4 to 2.8) | -7.7 (-28.2 to 20.1) |
| Morocco | 32530 (24557 to 40561) | 101.6 (77.3 to 124.4) | -20.8 (-40.4 to 2.3) | 340 (221 to 482) | 1.1 (0.7 to 1.6) | -14.1 (-35.5 to 14.8) |
| Mozambique | 19172 (14427 to 24941) | 165.3 (127.6 to 209.6) | 2.9 (-23.2 to 36.3) | 171 (109 to 248) | 1.6 (1 to 2.3) | 2.8 (-25.3 to 39.6) |
| Myanmar | 80586 (64659 to 100973) | 165 (134.3 to 204.2) | -57.1 (-68.1 to -42.4) | 765 (513 to 1073) | 1.7 (1.1 to 2.3) | -50.3 (-64.3 to -32.7) |
| Namibia | 1157 (876 to 1510) | 78.9 (61.7 to 100.1) | -20.6 (-40.6 to 9.4) | 12 (7 to 16) | 0.8 (0.5 to 1.2) | -14.8 (-36 to 15.3) |
| 1Nauru | 27 (21 to 34) | 447.5 (356.7 to 552.1) | -15.7 (-33.1 to 6.6) | 0 (0 to 0) | 4.2 (2.8 to 6) | -11 (-31.2 to 14.7) |
| Nepal | 48552 (36964 to 62221) | 206.6 (158.9 to 264.7) | -20.7 (-42.1 to 9.1) | 449 (295 to 638) | 2 (1.3 to 2.8) | -11.7 (-35.7 to 24) |
| Netherlands | 41592 (38196 to 44878) | 128.5 (118.7 to 138.4) | -49.2 (-52.9 to -45.4) | 979 (655 to 1389) | 2.9 (2 to 4.2) | -24.4 (-42.8 to -0.1) |
| New Zealand | 7070 (6588 to 7556) | 99.2 (92.9 to 105.7) | -49.8 (-53.5 to -45.9) | 150 (102 to 201) | 2 (1.4 to 2.7) | -28.3 (-43 to -11.2) |
| Nicaragua | 14291 (11518 to 17549) | 312.7 (253.9 to 377.5) | -18 (-33.9 to -0.3) | 166 (113 to 230) | 3.8 (2.6 to 5.2) | 2.9 (-20.9 to 30.8) |
| Niger | 24691 (18379 to 32199) | 304.1 (229.2 to 387.6) | -21.8 (-38.8 to 2.6) | 221 (143 to 320) | 3 (1.9 to 4.2) | -17.3 (-38.6 to 12.6) |
| Nigeria | 74390 (57243 to 95223) | 84.8 (66.7 to 107.1) | -19.1 (-37.8 to 7.7) | 723 (469 to 1042) | 0.9 (0.6 to 1.3) | -14.2 (-34.1 to 11.6) |
| Niue | 5 (4 to 6) | 238.6 (189.3 to 300) | -27.3 (-46.1 to -2.5) | 0 (0 to 0) | 2.7 (1.8 to 3.7) | -14.8 (-37 to 15.1) |
| North Macedonia | 11055 (8652 to 13920) | 342 (268.2 to 430) | -37.8 (-51.4 to -21.2) | 125 (81 to 179) | 3.9 (2.5 to 5.5) | -25.8 (-43.8 to -2.2) |
| Northern Mariana Islands | 141 (115 to 169) | 267.3 (221.4 to 318.9) | -37.6 (-52.1 to -21.7) | 2 (1 to 2) | 3.2 (2.2 to 4.4) | -27.1 (-44.3 to -5.2) |
| Norway | 7893 (7325 to 8473) | 87.1 (81 to 93.2) | -61.2 (-63.6 to -58.7) | 181 (127 to 244) | 1.9 (1.4 to 2.6) | -41.3 (-51.1 to -30.4) |
| Oman | 2802 (2402 to 3291) | 150.3 (130.8 to 173.5) | -49.7 (-61.8 to -30.5) | 34 (23 to 45) | 2.1 (1.5 to 2.8) | -31 (-49.8 to -1.7) |
| Pakistan | 202713 (164134 to 250299) | 161.2 (132.7 to 197.5) | -1.8 (-21.7 to 27.8) | 1762 (1190 to 2453) | 1.5 (1 to 2.1) | -1.4 (-22.5 to 31.9) |
| Palau | 67 (51 to 87) | 290.2 (224.8 to 372.9) | -18 (-41.3 to 14.2) | 1 (0 to 1) | 3.1 (2 to 4.4) | -7.8 (-35.2 to 33.4) |
| Palestine | 4321 (3658 to 5097) | 158.8 (135.2 to 185.3) | -41 (-56.2 to -19.2) | 43 (30 to 58) | 1.7 (1.2 to 2.3) | -33.4 (-52.2 to -6.7) |
| Panama | 10009 (7682 to 12902) | 240.8 (185 to 310.4) | -40.7 (-54.5 to -23.3) | 133 (87 to 191) | 3.2 (2.1 to 4.6) | -24.1 (-44.1 to 0.5) |
| Papua New Guinea | 19931 (13967 to 26882) | 339.9 (242.4 to 449) | -3.6 (-27.3 to 26.8) | 160 (99 to 241) | 3.1 (1.9 to 4.5) | -2.1 (-27.9 to 35) |
| Paraguay | 11371 (8582 to 14659) | 197.3 (149.5 to 253.3) | -19.1 (-39.6 to 9.1) | 122 (76 to 180) | 2.2 (1.4 to 3.2) | -9.9 (-34.6 to 22.6) |
| Peru | 124653 (92409 to 166059) | 383.3 (284.7 to 509.6) | -37 (-55.2 to -12.8) | 1532 (982 to 2270) | 4.8 (3.1 to 7.1) | -17.7 (-42.3 to 14.6) |
| Philippines | 91117 (74711 to 109754) | 106.4 (87.7 to 127.3) | -39.8 (-52.1 to -25.4) | 872 (586 to 1227) | 1.1 (0.7 to 1.5) | -35.5 (-49 to -18.4) |
| Poland | 141771 (117789 to 168395) | 216.7 (179.8 to 257.9) | -53.2 (-61.3 to -44.2) | 1498 (1038 to 2036) | 2.2 (1.5 to 3) | -47.7 (-56.4 to -37.7) |
| Portugal | 53958 (49837 to 57581) | 258.5 (240 to 276) | -58.1 (-60.9 to -55.1) | 833 (543 to 1169) | 3.9 (2.5 to 5.6) | -40.1 (-55.5 to -20.4) |
| Puerto Rico | 6353 (4927 to 8084) | 97.6 (75.4 to 126.5) | -58.1 (-67.9 to -45.5) | 109 (72 to 159) | 1.6 (1 to 2.3) | -43 (-57 to -23.9) |
| Qatar | 1248 (931 to 1668) | 135.2 (105.3 to 174.4) | -47.1 (-61.9 to -27.2) | 16 (10 to 23) | 2.1 (1.4 to 3) | -20.9 (-44.9 to 12.9) |
| Republic of Korea | 250946 (226275 to 276187) | 289.4 (262.5 to 317.6) | -78.5 (-80.6 to -76) | 8111 (5656 to 10998) | 9.3 (6.5 to 12.6) | -39.8 (-51.7 to -25.6) |
| Republic of Moldova | 13766 (11760 to 15917) | 246.1 (211.5 to 284.8) | -54.4 (-61 to -47.1) | 150 (105 to 203) | 2.7 (1.9 to 3.6) | -45.5 (-56.6 to -32.9) |
| Romania | 84586 (69028 to 103316) | 251.7 (204.9 to 309.1) | -32.3 (-45.2 to -16.7) | 1039 (709 to 1452) | 2.9 (2 to 4.1) | -16.2 (-36.6 to 5.7) |
| Russian Federation | 716147 (623533 to 823629) | 316.3 (274.2 to 363.9) | -57.6 (-63 to -51.3) | 9622 (6896 to 12660) | 4.2 (3 to 5.6) | -42.9 (-50.2 to -34.2) |
| Rwanda | 11683 (8764 to 14984) | 170.7 (131.2 to 214.6) | -52.2 (-64 to -37.3) | 102 (67 to 144) | 1.6 (1.1 to 2.3) | -47 (-60.8 to -28.4) |
| Saint Kitts and Nevis | 146 (115 to 177) | 212.7 (170.2 to 253.1) | -50.2 (-60.5 to -38.6) | 2 (1 to 2) | 2.6 (1.8 to 3.5) | -39.8 (-53.9 to -23.7) |
| Saint Lucia | 571 (479 to 675) | 263.6 (221.7 to 312.2) | -44 (-53.8 to -32.8) | 6 (4 to 8) | 2.9 (2 to 4) | -36.9 (-50.7 to -20.3) |
| Saint Vincent and the Grenadines | 341 (295 to 395) | 252.1 (219.2 to 292) | -32.6 (-43 to -21.3) | 4 (2 to 5) | 2.7 (1.9 to 3.6) | -28.1 (-42.7 to -10.1) |
| Samoa | 445 (341 to 573) | 290.7 (226 to 370.2) | -24.3 (-44.7 to 1.2) | 4 (3 to 6) | 2.9 (1.9 to 4) | -19.4 (-41.6 to 7.3) |
| San Marino | 213 (142 to 303) | 351.3 (227.9 to 508.2) | -36.3 (-60.1 to -2.7) | 5 (3 to 7) | 7.2 (4.7 to 10.5) | -17.7 (-39.8 to 14.4) |
| Sao Tome and Principe | 389 (306 to 496) | 364.5 (293.1 to 459.1) | -3 (-25.7 to 30.6) | 4 (2 to 5) | 3.7 (2.4 to 5.2) | 0.3 (-25.1 to 35.9) |
| Saudi Arabia | 18987 (14516 to 24711) | 85.1 (68.4 to 105.5) | -47.2 (-62.7 to -21.3) | 212 (142 to 303) | 1.1 (0.8 to 1.6) | -24.4 (-46.5 to 11) |
| Senegal | 21980 (16904 to 27564) | 285.8 (223.5 to 353.9) | -24.4 (-42.4 to -0.1) | 204 (135 to 291) | 2.8 (1.9 to 4) | -19.7 (-41.4 to 8.9) |
| Serbia | 29446 (23088 to 37017) | 197.5 (153.8 to 248.4) | -40.5 (-54.6 to -22.9) | 378 (245 to 545) | 2.5 (1.6 to 3.6) | -23.8 (-43.5 to 2.1) |
| Seychelles | 184 (157 to 215) | 159.4 (137.1 to 183.9) | -44 (-53.8 to -33.3) | 2 (1 to 3) | 1.8 (1.3 to 2.4) | -33.6 (-47.5 to -18) |
| Sierra Leone | 10921 (8147 to 14258) | 287.1 (218.3 to 371.8) | -19 (-39 to 7.7) | 98 (63 to 141) | 2.8 (1.8 to 4) | -16.5 (-38.7 to 12.8) |
| Singapore | 7979 (7265 to 8603) | 102.5 (92.7 to 110.4) | -76.5 (-78.5 to -74.5) | 265 (180 to 370) | 3.5 (2.3 to 4.8) | -51.5 (-62.6 to -38.2) |
| Slovakia | 16068 (12454 to 20065) | 180.3 (139.2 to 226) | -56.7 (-67 to -45.1) | 308 (202 to 436) | 3.4 (2.2 to 4.8) | -34.9 (-51.8 to -14) |
| Slovenia | 7050 (5510 to 9193) | 176 (135.6 to 229.6) | -60.7 (-72.7 to -44.3) | 136 (88 to 194) | 3.3 (2.2 to 4.9) | -36.1 (-56.8 to -7) |
| Solomon Islands | 2731 (1986 to 3463) | 659.4 (489.7 to 820.1) | -8.4 (-32.1 to 22.2) | 21 (13 to 30) | 5.6 (3.7 to 8) | -4.9 (-30.2 to 28.9) |
| Somalia | 22452 (16078 to 30383) | 271.2 (196.6 to 363.8) | -23.4 (-42.5 to 4.1) | 174 (106 to 260) | 2.4 (1.5 to 3.5) | -23 (-45.7 to 8.1) |
| South Africa | 59317 (55134 to 64085) | 126 (117.3 to 135.9) | -39 (-45.4 to -31.5) | 578 (411 to 750) | 1.3 (0.9 to 1.7) | -32.3 (-39.8 to -23.6) |
| South Sudan | 7310 (4987 to 10651) | 167.9 (117.6 to 236.6) | -30.7 (-50.2 to -3.5) | 62 (39 to 95) | 1.6 (1 to 2.4) | -27.9 (-48.4 to 1.8) |
| Spain | 125169 (115519 to 135123) | 143.4 (133.5 to 154.5) | -56.7 (-59.7 to -53.5) | 3129 (2041 to 4432) | 3.5 (2.3 to 5) | -29.4 (-47.2 to -6.6) |
| Sri Lanka | 27164 (20348 to 36103) | 106 (80 to 139.7) | -48.4 (-62.7 to -29.6) | 350 (217 to 527) | 1.4 (0.9 to 2.1) | -33.9 (-52.9 to -8) |
| Sudan | 72618 (47943 to 100052) | 350.6 (235.8 to 470.6) | -17.5 (-38.4 to 10.1) | 662 (388 to 985) | 3.4 (2.1 to 5.1) | -10.8 (-36.4 to 25.3) |
| Suriname | 1022 (839 to 1238) | 166.5 (137.4 to 200.5) | -29.9 (-43.1 to -12.5) | 10 (7 to 14) | 1.7 (1.2 to 2.4) | -25.5 (-41.6 to -5.2) |
| Sweden | 13823 (12765 to 14754) | 72.1 (67.3 to 76.6) | -63.2 (-65.5 to -60.9) | 290 (201 to 397) | 1.4 (1 to 2) | -49.4 (-59 to -38.3) |
| Switzerland | 13016 (11896 to 14048) | 81.3 (74.8 to 87.5) | -64.3 (-67.1 to -61.4) | 337 (228 to 483) | 2 (1.4 to 2.9) | -47.7 (-61 to -29.6) |
| Syrian Arab Republic | 13517 (9767 to 18476) | 105.9 (78.4 to 141.9) | -25.9 (-49.1 to 9.4) | 147 (91 to 217) | 1.2 (0.8 to 1.8) | -12.4 (-40.4 to 33.2) |
| Taiwan (Province of China) | 85658 (66233 to 112688) | 223.2 (173.5 to 291.5) | -45.5 (-57.8 to -29.4) | 1733 (1149 to 2456) | 4.5 (3 to 6.3) | -8.8 (-31.1 to 22.9) |
| Tajikistan | 33590 (26799 to 41919) | 584.6 (476 to 718.2) | -31.6 (-45.1 to -15.3) | 284 (190 to 404) | 5.5 (3.7 to 7.6) | -25.1 (-41.8 to -3.8) |
| Thailand | 128204 (94481 to 170076) | 127.9 (94.8 to 169.2) | -53.5 (-66.3 to -36.8) | 1580 (980 to 2325) | 1.6 (1 to 2.3) | -39.3 (-57.6 to -14.9) |
| The Republic of Côte d'Ivoire | 34730 (26066 to 44823) | 304.7 (238 to 382.2) | -28.3 (-45.9 to -8.2) | 305 (192 to 437) | 3 (1.9 to 4.2) | -23.6 (-42.8 to -0.3) |
| Timor-Leste | 1443 (1010 to 1843) | 171 (121.9 to 215.7) | -33.6 (-51.4 to -11) | 14 (9 to 20) | 1.7 (1.1 to 2.5) | -26.7 (-48.3 to -2) |
| Togo | 11779 (8889 to 15601) | 301 (235.7 to 389.7) | -20.7 (-38.9 to 4.2) | 104 (66 to 151) | 2.9 (1.9 to 4.2) | -17.7 (-39.4 to 12.9) |
| Tokelau | 3 (2 to 4) | 224.4 (170.8 to 287.9) | -31.8 (-50.8 to -8) | 0 (0 to 0) | 2.3 (1.5 to 3.4) | -23.8 (-44.7 to 3.9) |
| Tonga | 255 (202 to 317) | 314.3 (251 to 386.9) | -16.1 (-37.1 to 12.2) | 2 (2 to 3) | 3.1 (2 to 4.3) | -12.2 (-35.2 to 21) |
| Trinidad and Tobago | 1740 (1304 to 2290) | 94.7 (71.2 to 124.2) | -61.9 (-71.8 to -49.8) | 20 (13 to 30) | 1.1 (0.7 to 1.6) | -57.1 (-69.2 to -42.3) |
| Tunisia | 14082 (10230 to 19195) | 110.1 (80.7 to 149.3) | -27.7 (-49.8 to 4.6) | 177 (106 to 265) | 1.4 (0.9 to 2.1) | -11.3 (-39.7 to 28.6) |
| Turkey | 224602 (177969 to 277622) | 247.5 (196.5 to 304.3) | -52.5 (-63.3 to -38.2) | 2671 (1806 to 3780) | 3 (2 to 4.2) | -35.9 (-53.1 to -11.7) |
| Turkmenistan | 10282 (8073 to 13018) | 236.7 (187 to 297.8) | -59.3 (-67.7 to -49) | 95 (62 to 137) | 2.3 (1.5 to 3.3) | -55.6 (-67.4 to -41.8) |
| Tuvalu | 35 (26 to 47) | 333.9 (251.8 to 439.9) | -28.7 (-47.9 to -2.3) | 0 (0 to 0) | 3.1 (2 to 4.6) | -23.8 (-44.9 to 6.3) |
| Uganda | 34320 (26263 to 43598) | 207 (163.5 to 256.2) | -16.1 (-36.4 to 11.2) | 292 (193 to 407) | 1.9 (1.3 to 2.7) | -14.4 (-35.5 to 16.2) |
| Ukraine | 255778 (215078 to 303703) | 369.4 (309.5 to 440.6) | -51.6 (-59.8 to -41.8) | 3052 (2100 to 4118) | 4.2 (2.9 to 5.7) | -42.2 (-53.3 to -29) |
| United Arab Emirates | 8265 (5894 to 11270) | 175.8 (132.9 to 222.8) | -42.3 (-59 to -18.3) | 74 (45 to 112) | 2 (1.3 to 2.9) | -36 (-56.8 to -8.3) |
| United Kingdom | 125500 (118015 to 130499) | 105.4 (100.1 to 109.4) | -55.6 (-56.9 to -54.1) | 2495 (1752 to 3373) | 2 (1.4 to 2.7) | -39.2 (-49.6 to -26.7) |
| United Republic of Tanzania | 49273 (37991 to 61899) | 178.7 (141.5 to 219.2) | -28.2 (-44 to -8.7) | 435 (289 to 610) | 1.7 (1.1 to 2.4) | -25.3 (-43.1 to -2.3) |
| United States of America | 378118 (362536 to 391307) | 73.9 (71.2 to 76.4) | -40.2 (-41.9 to -38.1) | 9664 (6678 to 12671) | 1.8 (1.3 to 2.4) | -21.3 (-32.9 to -8.5) |
| United States Virgin Islands | 386 (318 to 457) | 216.3 (175.9 to 258.5) | -14.5 (-32.9 to 10.9) | 5 (3 to 6) | 2.5 (1.6 to 3.5) | -5.2 (-28.3 to 24.4) |
| Uruguay | 11543 (10676 to 12390) | 231.8 (214.5 to 248.4) | -40.6 (-45.4 to -35.6) | 146 (96 to 210) | 2.8 (1.8 to 4.1) | -32.1 (-48.6 to -10.3) |
| Uzbekistan | 84506 (69848 to 99483) | 334.8 (281.6 to 390) | -44.2 (-53.4 to -34.8) | 742 (507 to 1046) | 3.2 (2.2 to 4.6) | -39 (-52 to -24.7) |
| Vanuatu | 771 (560 to 1025) | 392.4 (285.9 to 516.5) | -4.7 (-30.7 to 31.8) | 6 (4 to 9) | 3.5 (2.2 to 5.1) | -4.5 (-31.1 to 34.4) |
| Venezuela (Bolivarian Republic of) | 83796 (63004 to 108713) | 282.6 (213.1 to 365.1) | -36.4 (-52.4 to -17.3) | 1006 (642 to 1475) | 3.5 (2.2 to 5.1) | -23.4 (-43.9 to 4.5) |
| Viet Nam | 238562 (186293 to 295736) | 237.5 (188.1 to 291) | -47.8 (-62 to -29.3) | 2545 (1694 to 3518) | 2.6 (1.7 to 3.6) | -36.3 (-54 to -11.9) |
| Yemen | 70305 (52168 to 95671) | 466.3 (354.9 to 618.1) | -10.5 (-34.4 to 28.1) | 607 (371 to 907) | 4.4 (2.8 to 6.5) | -5.4 (-34 to 37.8) |
| Zambia | 17019 (12563 to 22041) | 210.3 (160.6 to 264.8) | -28.8 (-46.5 to -7.1) | 142 (90 to 207) | 2 (1.3 to 2.8) | -26 (-46.5 to -0.7) |
| Zimbabwe | 25695 (19338 to 33283) | 323.9 (248.7 to 411.3) | 9 (-18.4 to 41.2) | 219 (139 to 320) | 3 (1.9 to 4.3) | 5 (-23.9 to 40) |

YLLs=years of life lost, YLDs=years lived with disability, UI=uncertainty interval.

Supplementary Table 6 SDI quintile in 2019

| SDI quintile | Lower bound | Upper bound |
| --- | --- | --- |
| Low SDI | 0 | 0.454743 |
| Low-middle SDI | 0.454743 | 0.607679 |
| Middle SDI | 0.607679 | 0.689504 |
| High-middle SDI | 0.689504 | 0.805129 |
| High SDI | 0.805129 | 1 |

SDI=Socio-demographic Index.
